# Supplementary material for: Subcellular glycan-mannose receptor binding kinetics correlate with myeloid cell function
Source: Nat Commun. 2025 Dec 26;17:886. doi: 10.1038/s41467-025-67602-x (PMC12830989; doi:10.1038/s41467-025-67602-x)
Supplement: Supplementary file 1 — Supplmentary Information [file 41467_2025_67602_MOESM1_ESM.pdf]

SUPPLEMENTARY INFORMATION

Subcellular glycan-mannose receptor binding kinetics correlate with myeloid cell function

Kas Steuten[1], Johannes J.A. Bakker[1], Ward Doelman[1], Diana Torres-García[1], Amit Cherian[1], Christian Kurts[2], Roger Riera[3], Lorenzo Albertazzi[3], Sander I. van Kasteren[1,4,5]*

1) Department of Chemical Biology and Immunology, Leiden Institute of Chemistry, Leiden University, Leiden, the Netherlands

2) Institute of Experimental Immunology, University Hospital of Bonn, Rheinische Friedrich Wilhelm University, 53127 Bonn, Germany

3) Department of Biomedical Engineering and Institute for Complex Molecular Systems, Eindhoven University of Technology, Eindhoven, the Netherlands

4) Institute of Chemical Immunology, Leiden University, Leiden, The Netherlands

5) Institute of Chemical Neuroscience (iCNS), Leiden University, Leiden, The Netherlands

*Correspondence: s.i.van.kasteren@chem.leidenuniv.nl

SUPPLEMENTARY INFORMATION 1

SUPPLEMENTARY FIGURES 2

SUPPLEMENTARY METHODS 22

LC-MS ANALYSIS 26

SUPPLEMENTARY REFERENCES 35

SUPPLEMENTARY SOFTWARE MANUAL 36

SUPPLEMENTARY FIGURES

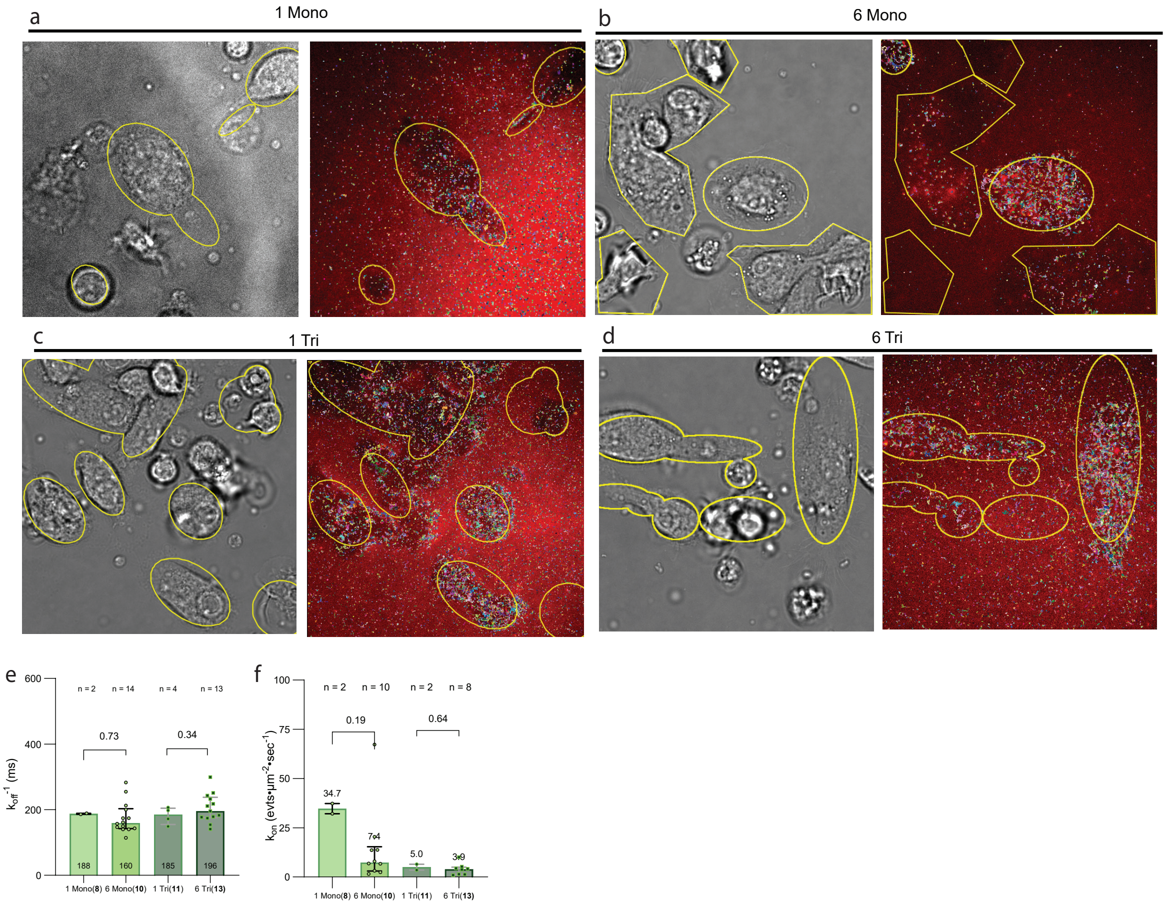

Supplementary Figure 1 | Manual analysis of glycan binding on dendritic cells is complicated by intrinsic cell heterogeneity and results in averaging artefacts. A) BMDC were incubated with 5 nM of fluorescent mannoside **8** in a live cell imaging chamber and a brightfield image was captured followed by a 2000 frame Glyco-PAINT recording. Closely-linked spots in subsequent frames were reconstructed into tracks representing binding events using the ImageJ TrackMate plugin and background filtering was performed by manual cell definition using ROI drawing (yellow areas). **B-D)**. identical analysis for glycans **10**, **11** and **13,** respectively **E)** Quantification of the number of events per area which is a relative measure of k_{on}. **F)** The average k_{off}^{-1} or τ for these events determined by fitting a one-phase exponential decay function over the binding event duration distribution histograms. n, depicts the number of recordings that were analyzed in total and were acquired over at least 3 biological replicates. However, not all recordings passed quality selection as detailed in methods section. Significance was assessed using two-way ANOVA followed by a Tukey post-hoc test. Tracks are colored randomly. Bar graphs and error bars represent mean ± interquartile range.

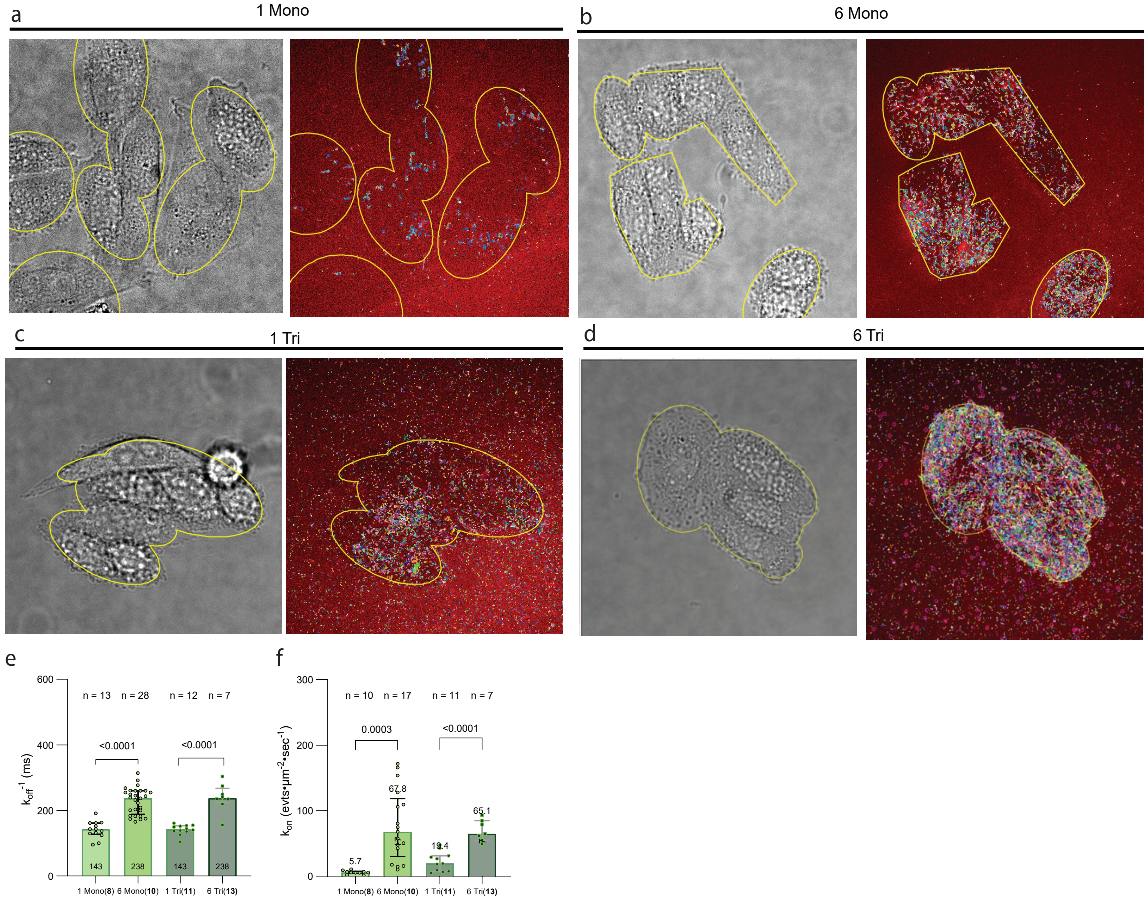

Supplementary Figure 2 | Manual analysis of glycan binding to CHO-MR using original Glyco-PAINT method. A) CHO-MR cells were incubated with 1 nM of fluorescent mannoside **8** in a live cell imaging chamber and a brightfield image was captured followed by a 2000 frame Glyco-PAINT recording. Closely linked spots were reconstructed into tracks representing binding events using ImageJ TrackMate plugin and background filtering was performed by manual cell definition using ROI drawing (yellow areas). **B-D)** identical analysis for glycans **10**, **11** and **13,** respectively. **E)** The average k_{off}^{-1} or τ for all tracks within the ROIs as determined by fitting a one-phase exponential decay function over the binding event duration distribution histograms. **E)** Quantification of the number of events per area which is a relative measure of k_{on}. n, depicts the number of recordings that were analyzed in total and were acquired over at least 3 biological replicates. However, not all recordings passed quality selection as detailed in methods section. Significance was assessed using two-way ANOVA followed by a Tukey post-hoc test. Tracks are colored randomly. Datapoints represent individually selected ROIs. Bar graphs and error bars represent mean ± interquartile range.

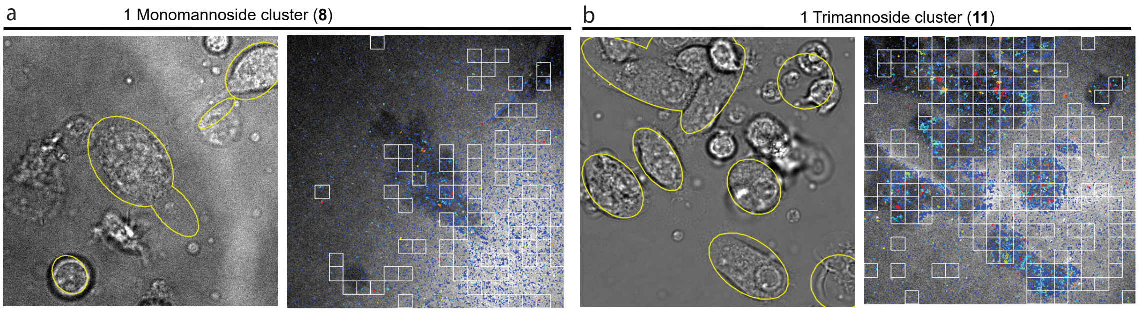

Supplementary Figure 3 | Glyco-PAINT-APP analysis of glycan binding to dendritic cells. A-B) Additional to main **Figure 2.** Brightfield and square-based detection of binding events for representative recordings of glycans **8** and **11**. Analysis and selection parameters are identical as in **Figure 2**.

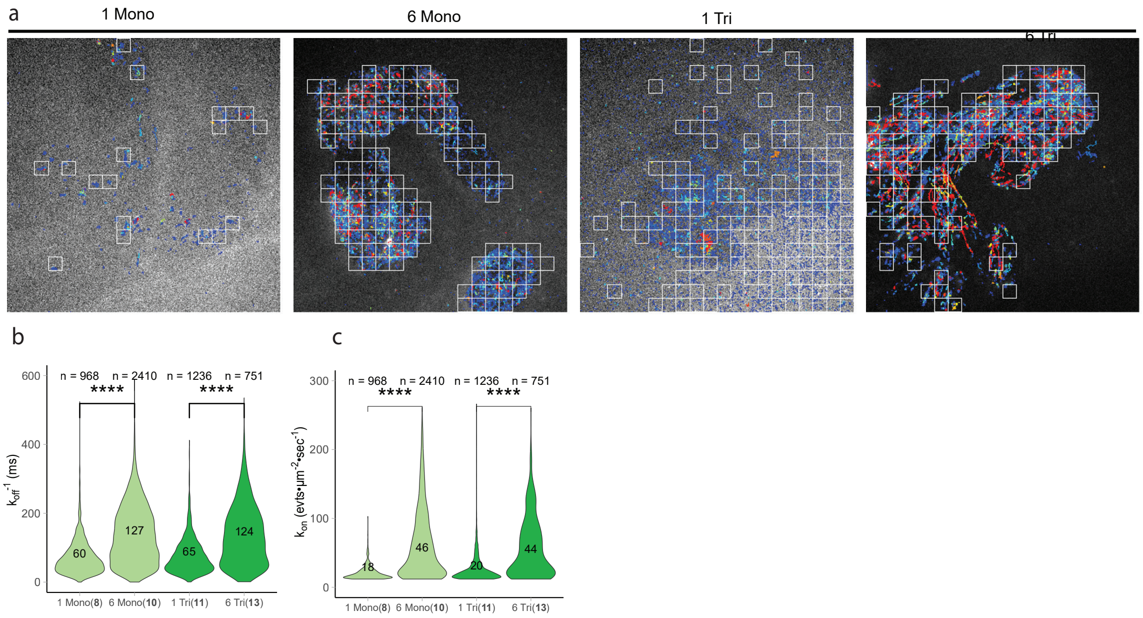

Supplementary Figure 4 | Glyco-PAINT-APP analysis of glycan binding to CHO-MR. A) Square-based detection of **8, 10, 11** and **13** binding to CHO-MR for identical recordings as in shown in **Supplementary Figure 2** using ROI analysis. **B)** The number of events per area which is a relative measure of k_{on}. **C)** average k_{off}^{-1} or τ for these events determined by fitting a one-phase exponential decay function over the binding event duration distribution histograms. n, depicts the number of squares that were analyzed per violin. Recordings were analyzed using a 20x20 grid and filtered with Density_Ratio > 2, R_Squared ≥ 0.9, Nr_Tracks/Square > 20. Significance was assessed using two-way ANOVA followed by a Tukey post-hoc test.

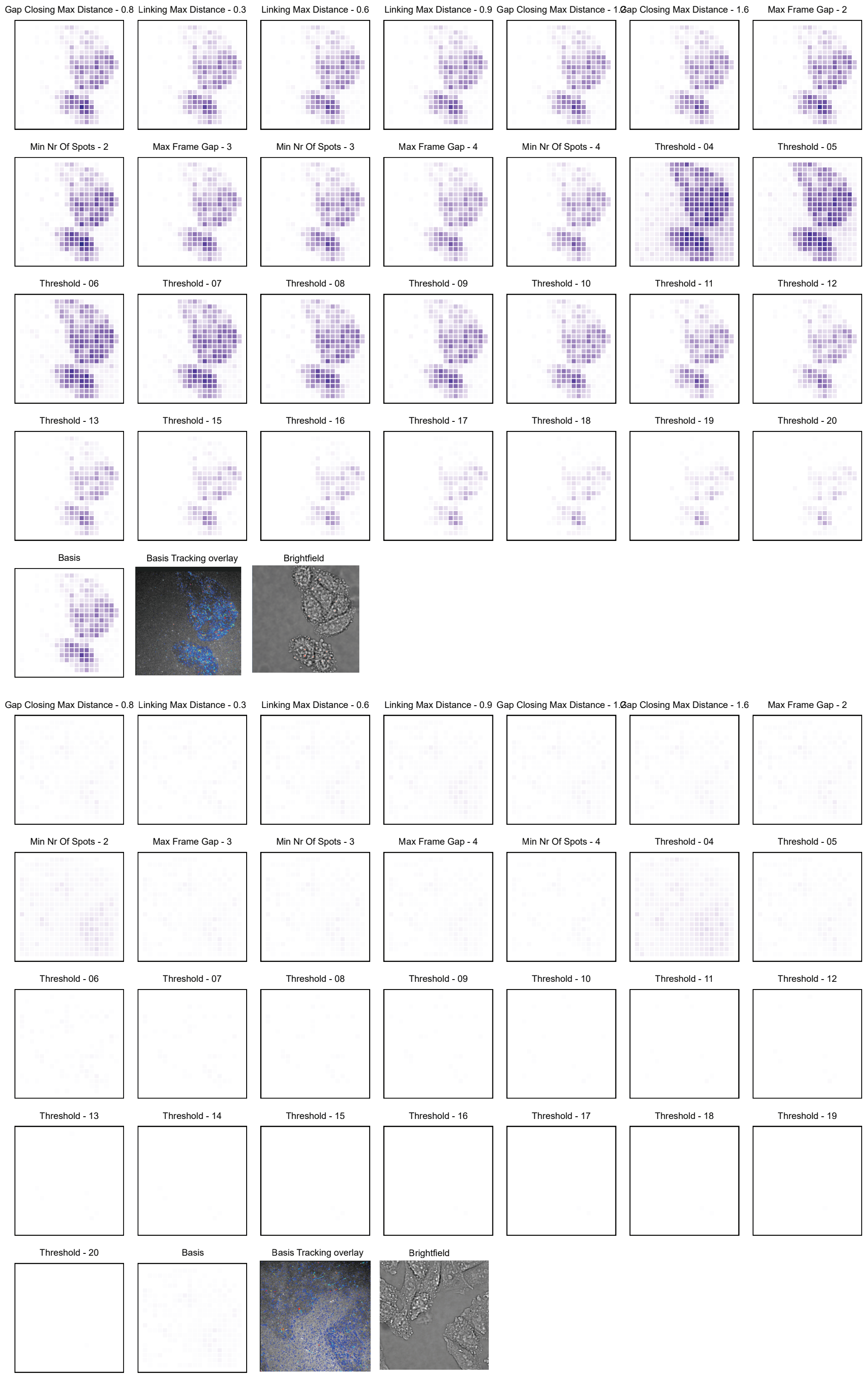

Supplementary Figure 5 | Tracking and spot detection parameter sweep for TrackMate analysis of Glyco-PAINT recordings. A) A single recording of glycan **13** binding to CHO-MR or CHO **B)** was processed with the indicated parameters for spot detection and track reconstruction. Heatmap images of detected tracks (rel. k_{on}) per parameter setting are depicted. Recordings were analyzed using

indicated deviating parameter from basis scenario, a 20x20 grid and filtered with Density_Ratio > 2, R_Squared ≥ 0.9 and Nr_Tracks/Square > 20.

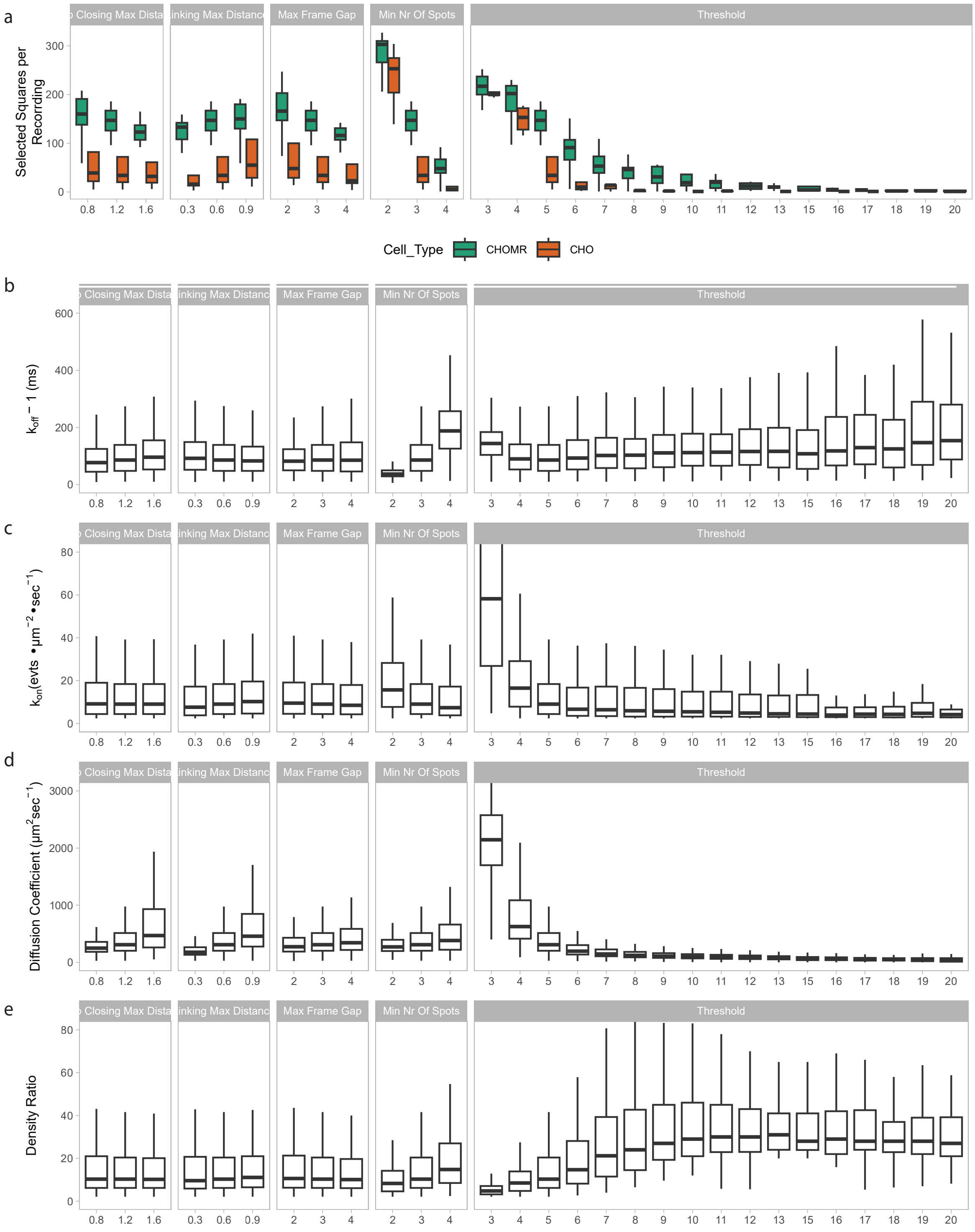

Supplementary Figure 6 | Quantification of tracking and spot detection parameter sweep for TrackMate analysis of Glyco-PAINT recordings. A) For each parameter setting as in **Supplementary Figure 5** the resulting number of squares that passed selection in a 20x20 grid, filtered with Density_Ratio > 2, R_Squared ≥ 0.9 and Nr_Tracks/Square > 20 is shown as boxplots comparing pooled data for glycans **8**, **10**, **11** and **13** binding to CHO versus CHO-MR. **B-E)** Same as in **A)** for kinetic parameters derived from CHO-MR binding only. Boxplots show median (line) and interquartile range (box); whiskers extend to ±1.5×IQR. Outliers beyond whiskers are omitted.

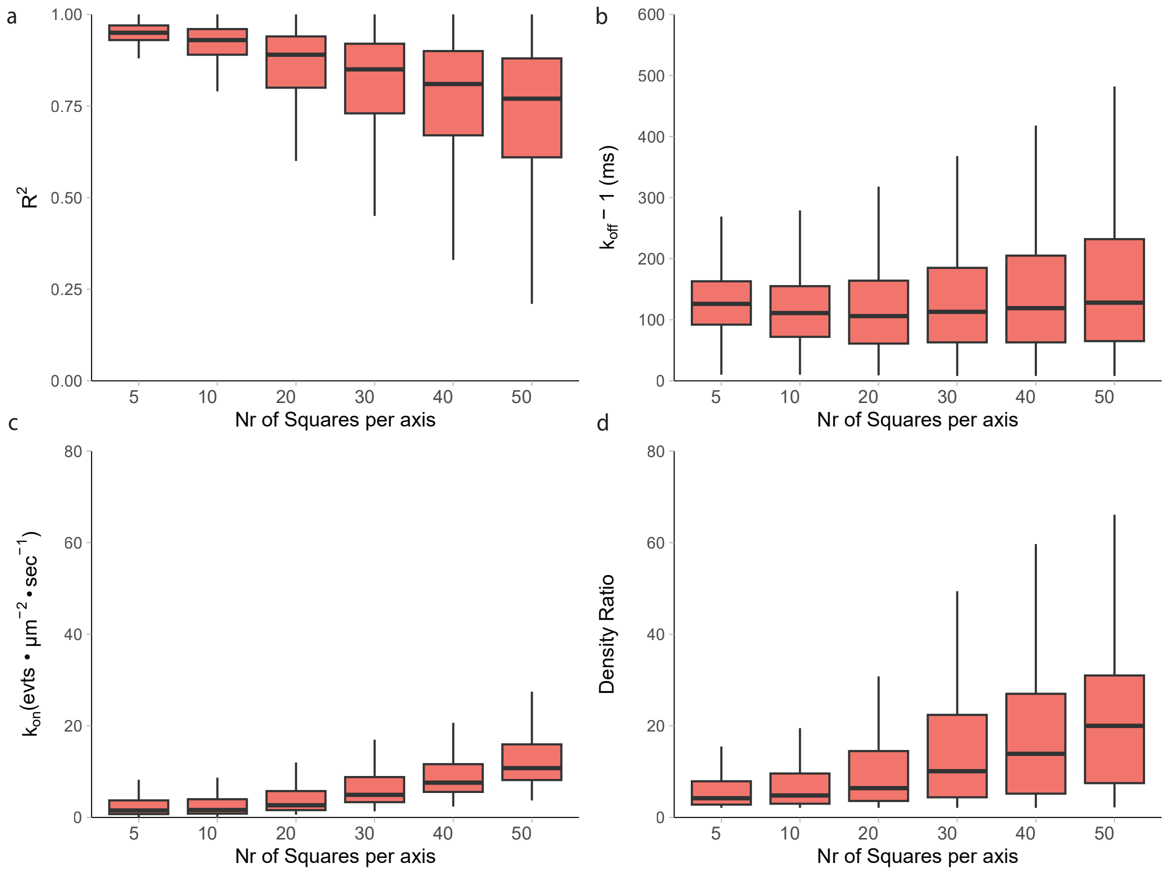

Supplementary Figure 7 | Quantification of the effects of grid size on Glyco-PAINT-APP parameters. Analysis of the effect of different grid sizes on statistical **A)** and kinetic **B-D)** parameters for all glycans binding to BMDC. Boxplots show median (line) and interquartile range (box); whiskers extend to ±1.5×IQR. Outliers beyond whiskers are omitted.

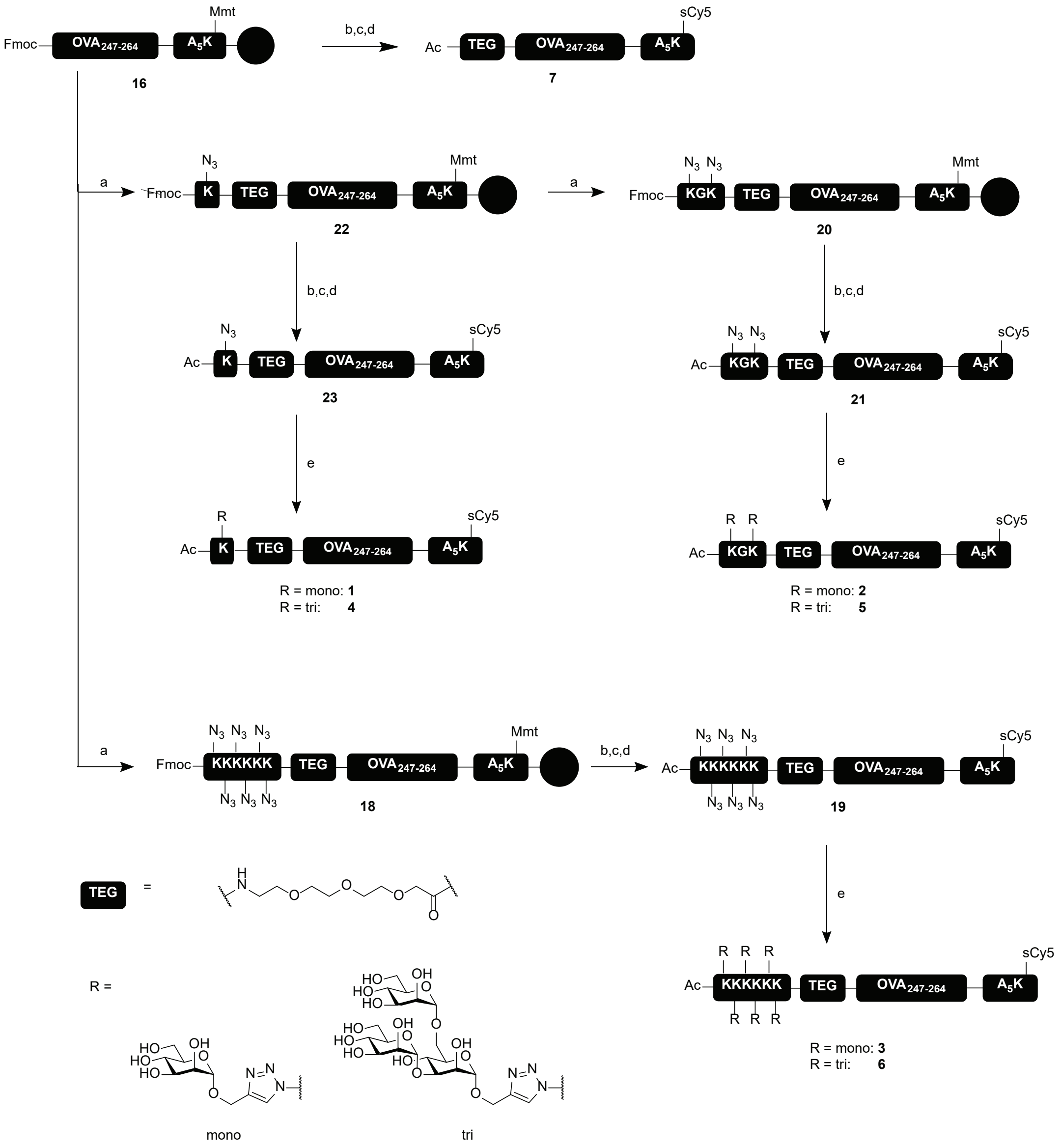

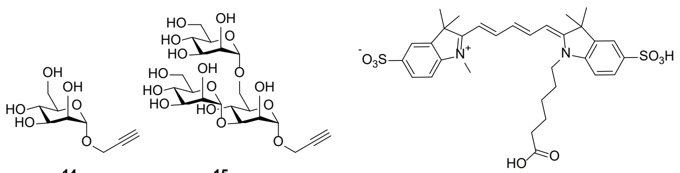

14 15 sCy5-COOH

Supplementary Figure 8 | Synthetic scheme for glycopeptides 1-7. Reagents and conditions: a) SPPS (HCTU, Fmoc-TEG-OH, Fmoc-Lys(N_3)-OH) b) i) 20 % (v/v) piperidine/DMF ii) Ac_2O, DiPEA, DMF c) i) AcOH, TFE, DCM ii) Et_3N, DMF iii) **sCy5-COOH**, HCTU, DiPEA, DMF d) i) TFA, TIS, H_2O ii) RP-HPLC e) i) **14**

or **15**, $CuSO_4$, NaAsc, THPTA, DMSO, 40°C ii) SEC. Synthetic procedures have been described for propargyl mannosides **14** and **15** by Riera et al.[67] and Hogervorst and Li et al.[31] respectively.

Cell Type	Adjuvant	Probe	Tau	Density	TTD	LTD	STD	DC	Speed	Max Speed
CHOMR	None	1 Mono	69	4.7	17.7	1.55	0.1	231.1	2.1	3.2
CHOMR	None	2 Mono	93	7.5	28.5	1.52	0.1	440.6	2.3	4.5
CHOMR	None	6 Mono	118	9.7	35.5	1.43	0.1	457.5	2.1	4.5
CHOMR	None	1 Tri	80	5.7	21.4	1.55	0.1	255.7	1.7	3.2
CHOMR	None	2 Tri	120	11.9	43	1.4	0.1	420	1.9	3.2
CHOMR	None	6 Tri	108	8.1	34.1	1.8	0.1	473.8	2.3	4.5
CHOMR	None	Control	75	2.9	16.8	1.3	0.1	173.8	1.6	3.2
CHOMR	CytD	1 Mono	65	3.8	9.9	1	0.1	102.7	1.6	3.2
CHOMR	CytD	2 Mono	85	5.2	15.9	1.02	0.1	297.8	2.2	4.5
CHOMR	CytD	6 Mono	114	10.6	32.6	1.15	0.1	671.3	2.6	4.5
CHOMR	CytD	1 Tri	143	14.3	51.6	1.3	0.1	576.8	2.6	4.5
CHOMR	CytD	2 Tri	108	7.8	32.3	1.4	0.1	505.9	2.3	4.5
CHOMR	CytD	6 Tri	91	5.5	15.4	0.9	0.1	357.6	2.4	4.5
CHOMR	CytD	Control	55	4	11.8	1.15	0.1	202.9	2.3	4.5
BMDC	None	1 Mono	87	2.3	22.5	1.15	0.1	369.6	2.2	4.5
BMDC	None	2 Mono	98	2.7	33.5	1.7	0.1	370.9	2.5	4.5
BMDC	None	6 Mono	77	2.8	22.6	1.65	0.1	310.1	2.1	4.5
BMDC	None	1 Tri	89	2.5	24.4	1.27	0.1	266	1.6	3.2
BMDC	None	2 Tri	72	1.7	17.5	1.65	0.1	250.7	1.9	3.2
BMDC	None	6 Tri	70	2.3	18.3	1.55	0.1	282.7	2.1	4.5
BMDC	None	Control	93	4.3	24.4	1.18	0.1	411.3	2.4	4.5
BMDC	CytD	1 Mono	98	4.9	25.8	1	0.1	386.4	2.1	3.2
BMDC	CytD	2 Mono	83	2.9	20.4	1.23	0.1	483.1	2.7	4.5
BMDC	CytD	6 Mono	116	6.9	36.9	1	0.1	463.2	2.3	4.5
BMDC	CytD	1 Tri	124	7.2	42.1	1.15	0.1	511.9	2.3	4.5
BMDC	CytD	2 Tri	111	5.8	34.2	1.12	0.1	573.6	2.7	4.5
BMDC	CytD	6 Tri	76	2.7	16.6	1.25	0.1	263.4	2.1	4.5
BMDC	CytD	Control	105	5.5	30.6	1.1	0.1	352.6	2.1	3.9
BMDCMRko	None	1 Mono	176	10	94.6	1.95	0.1	603.9	1.8	4.5
BMDCMRko	None	2 Mono	158	7.4	63.7	1.7	0.1	339.1	1.6	3.2
BMDCMRko	None	6 Mono	180	10.7	101.7	1.9	0.1	573.6	1.9	4.5
BMDCMRko	None	1 Tri	169	7.3	65.6	1.75	0.1	375	1.6	3.2
BMDCMRko	None	2 Tri	143	6.9	57.5	1.8	0.1	457.2	1.9	4.5
BMDCMRko	None	6 Tri	116	4.2	31.4	1.7	0.1	297.7	1.6	3.2
BMDCMRko	None	Control	163	9.8	84.9	1.82	0.1	445.3	1.6	3.2

Table S1 | Median values of Glyco-PAINT-APP derived kinetics for probe 1-7 binding to BMDC, BMDC MR$^{-/-}$ and CHO-MR. Square selection criteria as in **Figure 4A-B**. Cells were treated with 10 µM Cytochalasin D for 30 min in indicated tables. TTD, total track duration LTD, long track duration. STD, short track duration. DC, diffusion coefficient.

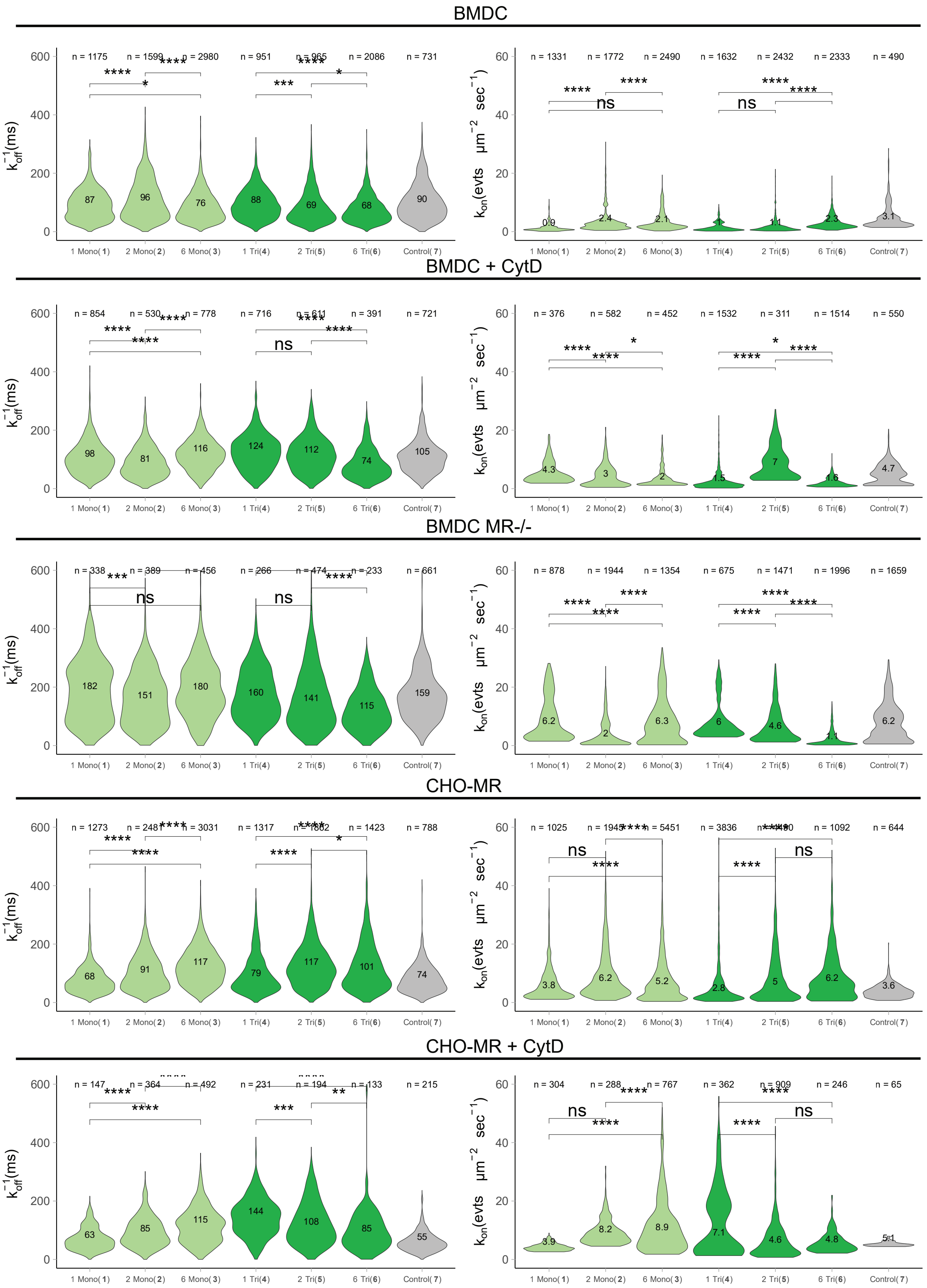

Supplementary Figure 9 | Violin distribution of Glyco-PAINT-APP derived k_{on} and k_{off}^{-1} for glycan SLP 1-7 binding to BMDC, BMDC MR$^{-/-}$ and CHO-MR. Square selection criteria as in main **Figure 4.** n, indicates the number of squares that were selected per violin. Significance across probes of similar

glycan structure was assessed using two-way ANOVA followed by a Tukey post-hoc test. Values in violin represent medians.

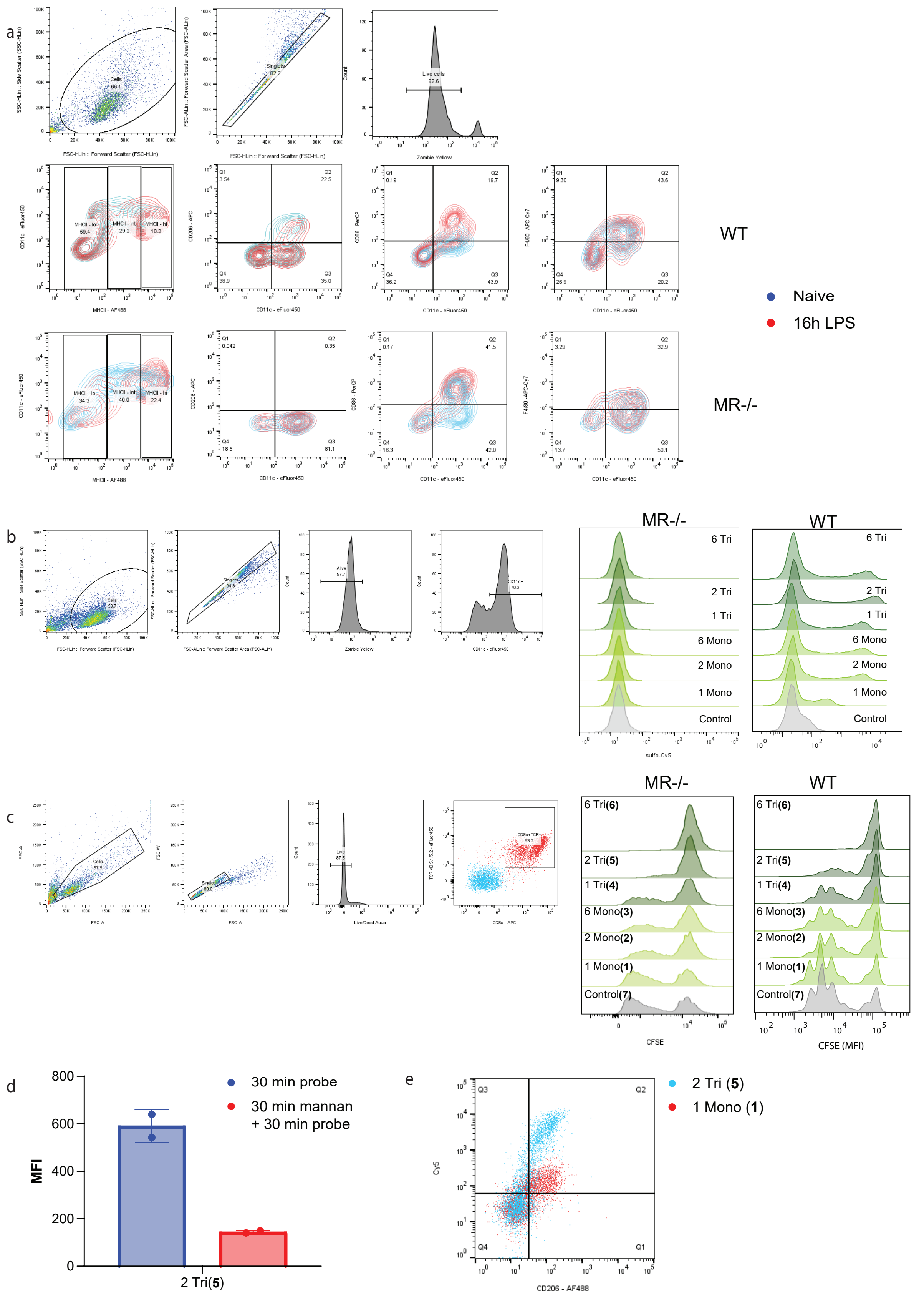

Supplementary Figure 10 | Flow cytometry assays for uptake and cross-presentation of glycan SLP 1-7 A) Characterization of BMDC WT and MR$^{-/-}$ cell surface marker expression and functional response to 100 ng/mL LPS stimulation for 16h by flow cytometry. Shown is the gating strategy and expression

profiles of CD11c, MHCII, CD206, CD86 and F4-80. **B)** Gating strategy for glycan uptake experiments in BMDC WT and $MR^{-/-}$. **C)** Same as in **B)** for OT-I T cell proliferation experiments. **D)** Endocytosis of **5** is blocked by pre-incubation of BMDC with 2 mg/mL mannan for 30 min. **E)** Mannosylated antigens **1** and **5** are taken up by $CD11c^{+}CD206^{+}$ BMDC.

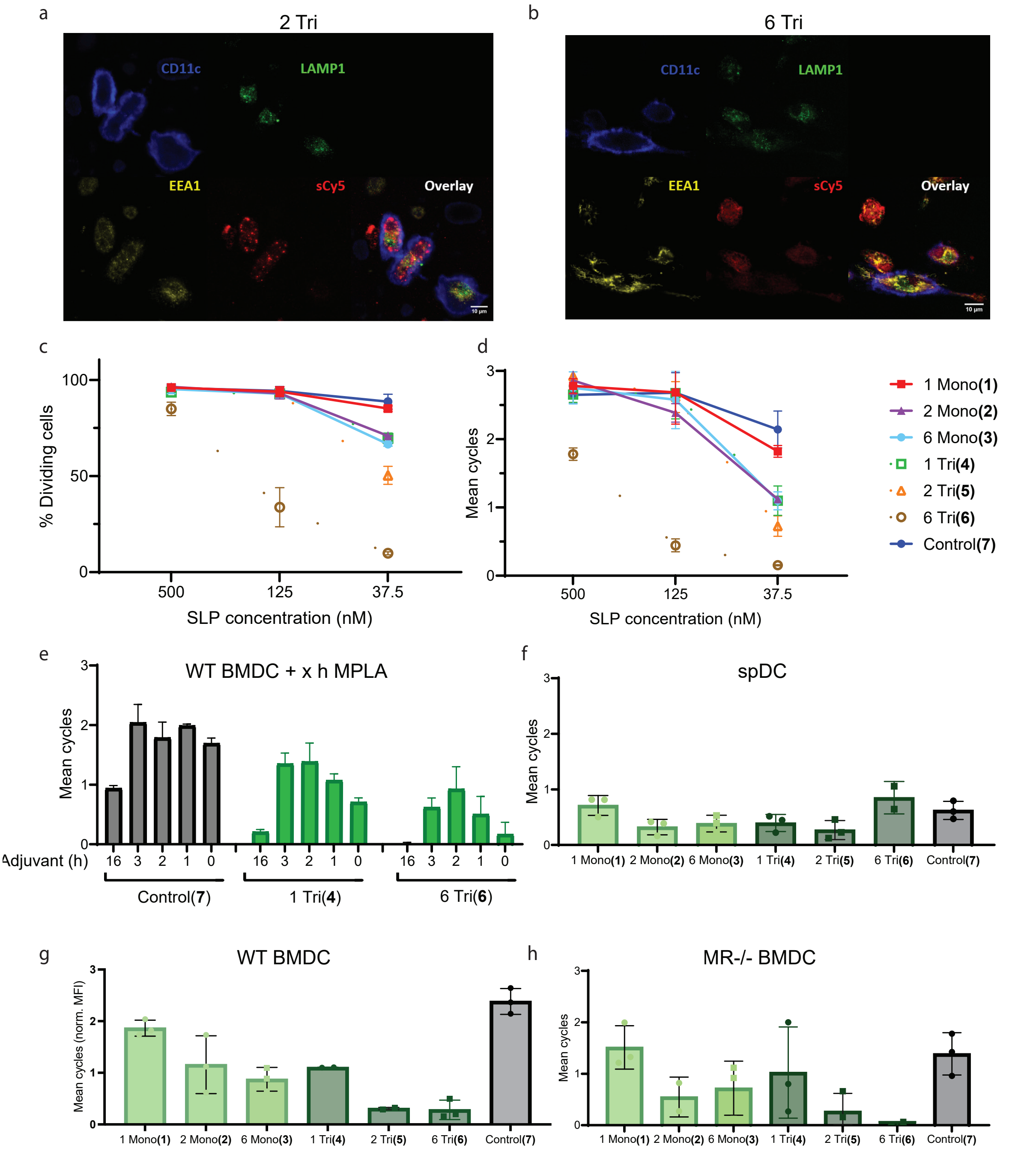

Supplementary Figure 11 | Uptake and cross-presentation of 1-7. A, B) Confocal imaging of glycan SLP uptake by BMDC. Cells were pulsed for 1h with 250 nM **12** or **13** after being fixed and permeabilized and then stained for CD11c, EEA1 and LAMP1 using fluorophore labeled antibodies. **C-D)** Cross-presentation by BMDC pulsed with different concentrations of antigen **1-7**. Amounts of cross-presentation were determined by flow cytometric measurement of CFSE-dilution by proliferating cognate T cells and quantified using mean cycle or percentage dividing cells of DMSO. **E)** Pretreatment of BMDC with 1 µg/mL MPLA for indicated time before 2h pulse with antigen **1-7** and 3d coculture with OT-I cells. **F)** Cross-presentation of antigens **1-7** by magnetically purified splenic DC. **G)** Cross-presentation of immature BMDC pulsed with SLPs **1-7**. **H)** Cross-presentation of BMDC MR$^{-/-}$ pulsed with SLPs **1-7**.

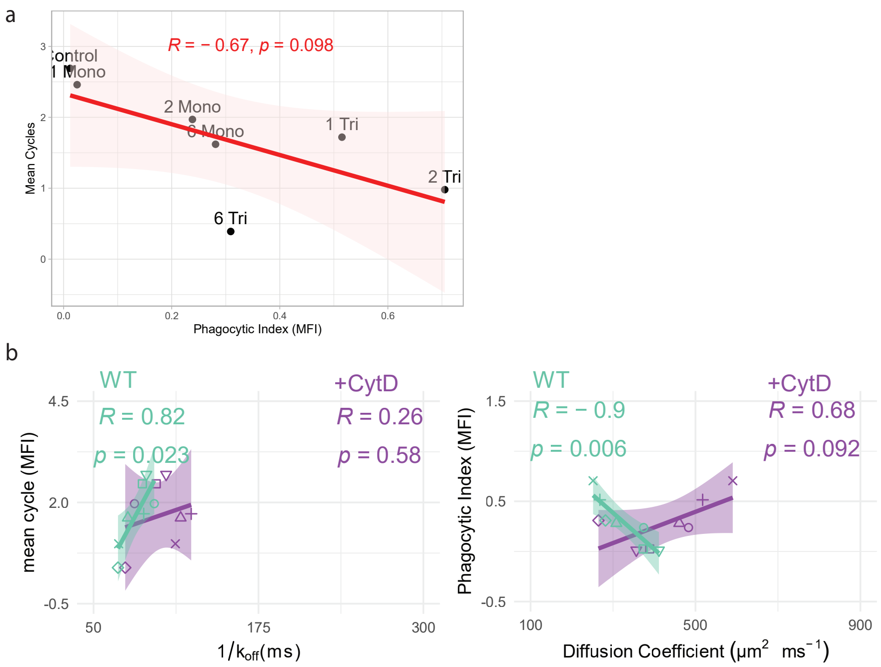

Supplementary Figure 12 | Correlation between phagocytic index, T cell proliferation and Glyco-PAINT-APP kinetics 1-7 by BMDC. A) Glycan uptake and T cell proliferation (mean cycle) correlation. **B)** Correlation analysis of Glycan SLP functionality (T cell proliferation and uptake) with k_{off}^{-1} and diffusion coefficients as determined by Glyco-PAINT-APP for untreated or CytD treated BMDC. Pearson's R and two-tailed t test P value are displayed in the correlation plots and the shaded area represents the 95% confidence interval of the regression line.

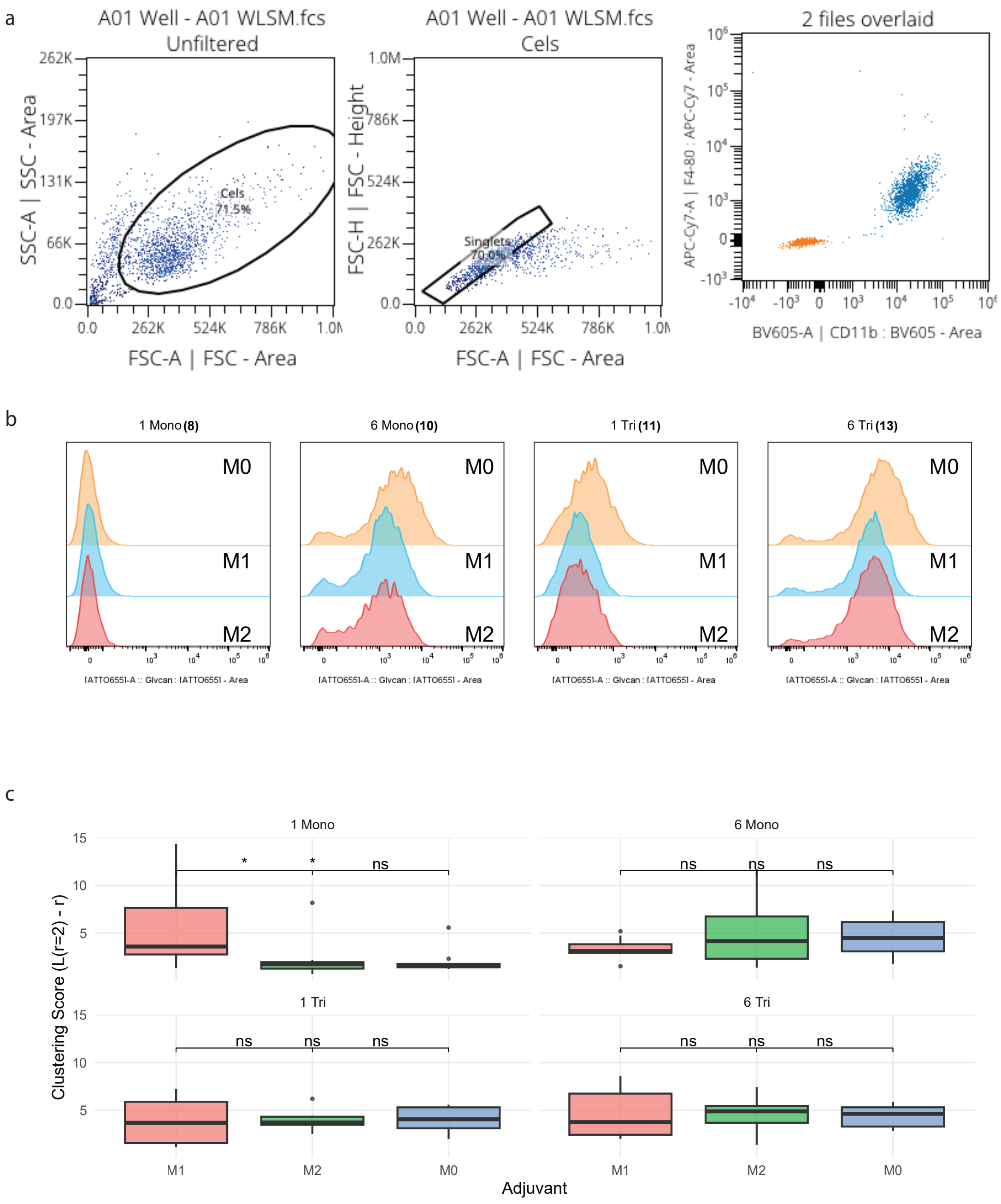

Supplementary Figure 13 | Glycan uptake and clustering of binding events by macrophages. A) Gating strategy for flow cytometric quantification of glycan **8**, **10**, **11** or **13** uptake by BMDM. **B)** Flow cytometry histograms of relative uptake of glycans by BMDM treated with M0-2 polarization stimuli. **C)** Clustering analysis of Glyco-PAINT-APP binding events on polarizing macrophages. Clustering score was determined using Ripley-K clustering algorithm with r (radius) of 1 µm. See experimental methods for further details.

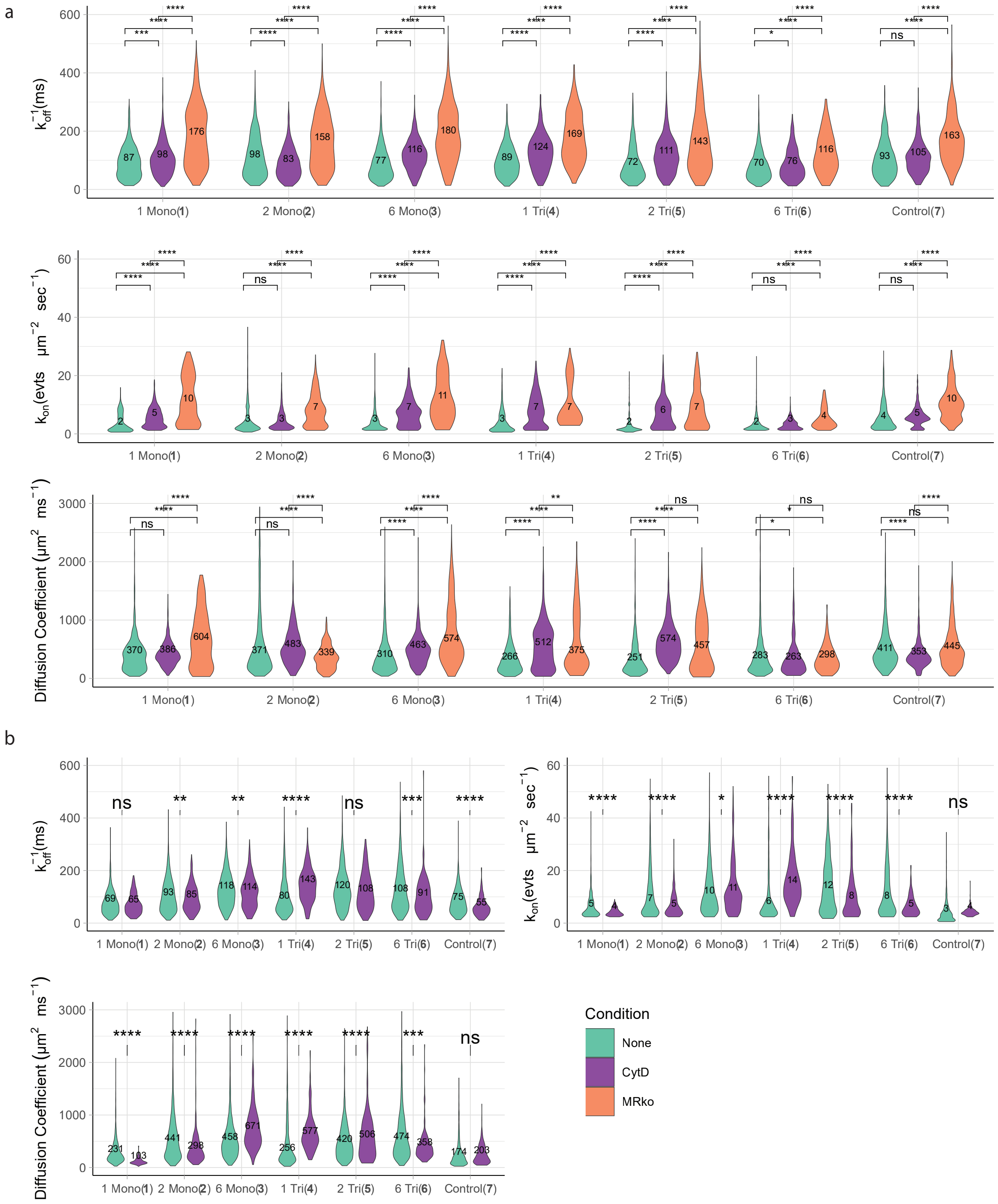

Supplementary Figure 14 | Violin distribution of Glyco-PAINT-APP derived k_{on} and k_{off}^{-1} for glycan SLP 1-7 binding to BMDC, BMDC MR$^{-/-}$ and CHO-MR treated with CytD. Square selection criteria as in main **Figure 4.** n, indicates the number of squares that were selected per violin. Significance across cell types or CytD treatment was assessed using two-way ANOVA followed by a Tukey post-hoc test. Numerical values in violin plots represent medians.

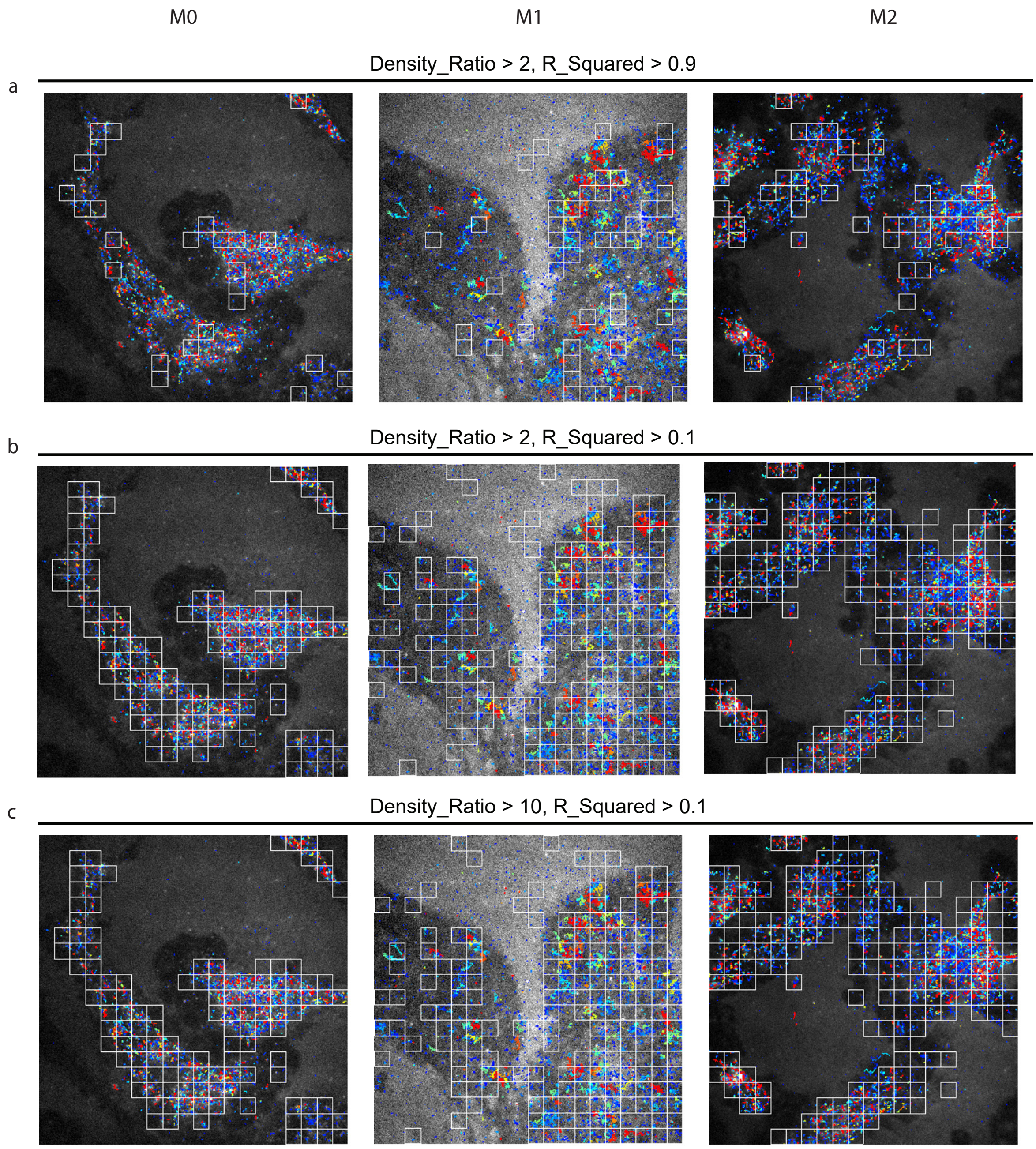

Supplementary Figure 15 | Square selection criteria for macrophages. Identical recordings of glycan **8** binding to M0, M1 and M2 macrophages with varying Density_Ratio and R_Squared selection criteria set to R_Squared > 0.9 (standard) in **A)**, R_Squared > 0.1 in **B)** or Density_Ratio > 10 in **C)**.

SUPPLEMENTARY METHODS

General procedure for automated SPPS

Peptides were synthesized using automated Fmoc-SPPS on a Liberty Blue[tm] automated microwave peptide synthesizer (CEM corporation). Synthesis was performed 100 µmol scale on Tentagel S RAM resin (loading 0.20-0.25 mmol/g, Rapp Polymere GmbH, Germany). Resin was first swollen for 5 minutes in DMF prior to amino acid coupling. Activation was achieved using DIC/Oxyma coupling as recommended by the manufacturer. The following amino acids were used: Fmoc-Ala-OH, Fmoc-Arg(Pbf)-OH, Fmoc-Asn(Trt)-OH, Fmoc-Asp(OtBu)-OH, Fmoc-Gln(tBu)-OH, Fmoc-Glu(OtBu), Fmoc-Gly-OH, Fmoc-Ile-OH, Fmoc-Leu-OH, Fmoc-Lys(Boc)-OH, Fmoc-Lys(Mmt)-OH, Fmoc-Phe-OH, Fmoc-Ser(tBu)-OH, Fmoc-Val-OH. All amino acids were obtained from Novabiochem except Fmoc-Lys(Mmt)-OH which was obtained from CEM corporation. Standard coupling was achieved using 5 equivalents amino acid as a 0.2 M amino acid/DMF solution, 5 equivalents DIC as a 0.5 M of DIC/DMF solution and 5 equivalents Oxyma as a 1 M Oxyma/DMF solution (also containing 0.2 M DiPEA), at 90°C for 2 minutes. Standard Fmoc deprotection was achieved by 20% (v/v) piperidine in DMF at 90°C for 90 seconds, repeated once. To analyze the quality of the peptide, a small amount of resin (~1mg) was treated with 200 µL of a TFA cocktail (95:2.5:2.5, TFA/H_2O/TIS) for 2 hours, after which the TFA was filtered into 800 µL of ice cold Et_2O. After five minutes the formed precipitate was collected by centrifugation and the supernatant discarded. The pellet was dissolved in 200 µL 1:1:1 H_2O/MeCN/tBuOH and subjected to LC-MS analysis. Peptides were characterized using electrospray ionization mass spectrometry (ESI-MS) on a Thermo Finnigan LCQ Advantage Max LC-MS instrument with a Surveyor PDA plus UV detector on an analytical C18 column (Phenomenex, 3 µm, 110 Å, 50 mm × 4.6 mm) in combination with buffers A (H_2O), B (MeCN), and C (1% aq TFA). Quality of crude was evaluated with a linear gradient of 10-90% B with a constant 10% C over 10 minutes.

General procedures for manual SPPS

Manual elongation of peptides was carried out in a fritted syringe at either 25 or 5 µmol scale. Fmoc deprotection was achieved using 20 % (v/v) piperidine in DMF in two steps, reacting 3 and 7 minutes, respectively. Fmoc-Gly-OH was coupled using 5 equivalents of amino acid together with 5 equivalents of HCTU (as a 0.5 M solution) and 10 equivalents DiPEA for 45 minutes. Fmoc-Lys(N_3)-OH was coupled using 2 equivalents together with 2 equivalents HCTU (as a 0.2 M in DMF solution) and 4 equivalents of DiPEA for 90 minutes. Fmoc-TEG-OH was coupled using 4 equivalents, together with 4 equivalents HCTU and 8 equivalents DiPEA in 1 mL of DMF for 45 minutes. Analysis of the quality of the resin-bound peptide was carried out as above.

General procedure for *N*-terminal acetylation

The *N*-terminal Fmoc was cleaved by treating the resin twice with a 20% (v/v) solution of piperidine in DMF, for 3 and 7 minutes. This was followed by the addition of a solution containing 10 % (v/v) Ac_2O and 5 % (v/v) DiPEA in DMF (1 mL for 25 µmol resin bound peptide). This resin was acetylated for 15 minutes under gentle agitation, followed by draining the acetylation solution and thorough washing of the resin with DMF.

General procedure for fluorophore labeling

Fluorophore labeling of peptides was carried out on a 5 µmol scale. The resin was first swelled in DCM, followed by selective Mmt deprotection. The Mmt group protecting the C-terminal lysine residue, the resin was treated with a mildly acidic mixture consisting of 10 % (v/v) AcOH and 20 % (v/v) TFE in DCM (1 mL) for one hour[73]. After this time has elapsed, the resin was washed three times with DCM and three times with DMF. To remove residual acetic acid, the resin was then treated with a 10 % TEA in DMF mixture (2 x 10 min) followed by an additional three washes with DMF. One equivalent of fluorophore was dissolved in a 50 mM solution of HCTU together with 2 equivalents of DiPEA. This

mixture was added to the drained resin and allowed to react under gentle agitation overnight protected from light. After overnight coupling the solution was drained and the resin was thoroughly washed with DMF.

General procedure for peptide global deprotection and purification

Peptide cleavage was carried out using a standard TFA cleavage cocktail (TFA:H_2O:TIS 95:2.5:2.5) using 1 mL per 25 µmol of resin bound peptide. Before initiating cleavage, the resin was thoroughly washed with DCM and drained. The TFA cocktail was added and mixed with the resin under gentle agitation for one hour, after which it was drained into a centrifuge tube containing ice cold diethyl ether (10:1 ratio Et_2O:TFA). When deprotecting fluorophore modified peptides, the cleavage reaction was protected from light as much as possible and a 20:1 ratio of Et_2O to TFA was used. The diethylether/TFA mixture was chilled for a minimum of 10 minutes to increase peptide recovery, and the precipitated peptide was recovered by centrifugation. The supernatant was discarded and the pellet washed with a small amount of Et_2O, followed again by centrifugation. This pellet was dissolved in a mixture of H_2O:MeCN:tBuOH and subjected to RP-HPLC purification on a Gilson GX281 semipreparative HPLC. This machine was equipped with a Gemini-NX C18 column (5 µm, 110 A, 250 x 10.0 mm) using a flow of 5 mL/min and buffers A = 0.1% TFA in H_2O and B = MeCN. Peak detection was done using a UV-Vis detector set to 225 nm or 610 nm for fluorescently labeled peptides. Quality of purified peptides was determined using an electrospray ionization mass spectrometry (ESI-MS) on a Thermo Finnigan LCQ Advantage Max LC-MS instrument with a Surveyor PDA plus UV detector on an analytical C18 column (Phenomenex, 3 µm, 110 Å, 50 mm × 4.6 mm) in combination with buffers A (H_2O), B (MeCN), and C (1% aq TFA). Quality of the peptides was evaluated with a linear gradient of 10-50% B with a constant 10% C over 9 minutes or a linear gradient of 10-90% B with a constant 10% C over 9 minutes.

Fmoc-Lys(N_3)-Lys(N_3)-Lys(N_3)-Lys(N_3)-Lys(N_3)-Lys(N_3)-TEG-Asp(OtBu)-Glu(OtBu)-Val-Ser(tBu)-Gly-Leu-Glu(OtBu)-Gln(Trt)-Leu-Glu(OtBu)-Ser(tBu)-Ile-Ile-Asn(Trt)-Phe-Glu(OtBu)-Lys(Boc)-Leu-Ala-Ala-Ala-Ala-Ala-Lys(Mmt)-RAM-Tentagel S (18)

Resin bound peptide **16** (25 µmol) was elongated manually with Fmoc-TEG-OH and Fmoc-Lys(N_3)-OH. **LC-MS** RT = 7.6 min (C18, 10-90% B over 9 minutes) **LRMS** calcd $[M+2H]^{2+}$ = 1941.52, $[M+3H]^{3+}$ = 1294.68 observed M/z = 1941.53, 1294.80

Ac-Lys(N_3)-Lys(N_3)-Lys(N_3)-Lys(N_3)-Lys(N_3)-Lys(N_3)-TEG-Asp-Glu-Val-Ser-Gly-Leu-Glu-Gln-Leu-Glu-Ser-Ile-Ile-Asn-Phe-Glu-Lys-Leu-Ala-Ala-Ala-Ala-Ala-Lys(sCy5)-NH_2 (19)

Resin bound peptide **18** (5 µmol) was N-terminally acetylated according to the standard conditions. This was followed by chemoselective deprotection of the Mmt group and coupling of sCy5-OH (1.0 eq, 3.2 mg, 5 µmol) as described in the general methods. Global deprotection followed by RP-HPLC purification yielded compound **19** as a blue solid (1.60 mg, 0.37 µmol, 7.4%). **LC-MS** RT = 6.0 min (C18, 10-90% B over 9 minutes) **LRMS** calcd $[M+3H]^{3+}$ = 1443.06 observed M/z = 1443.00

Fmoc-Lys(N_3)-Gly-Lys(N_3)-TEG-Asp(OtBu)-Glu(OtBu)-Val-Ser(tBu)-Gly-Leu-Glu(OtBu)-Gln(Trt)-Leu-Glu(OtBu)-Ser(tBu)-Ile-Ile-Asn(Trt)-Phe-Glu(OtBu)-Lys(Boc)-Leu-Ala-Ala-Ala-Ala-Ala-Lys(Mmt)-RAM-Tentagel S (20)

Resin bound peptide **22** (25 µmol) was elongated manually with Fmoc-TEG-OH, Fmoc-Lys(N_3)-OH and Fmoc-Gly-OH. **LC-MS** RT = 6.5 min (C18, 10-90% B over 9 minutes) **LRMS** calcd $[M+2H]^{2+}$ = 1661.86, $[M+3H]^{3+}$ = 1108.24 observed M/z = 1662.00, 1108.67

Ac-Lys(N_3)-Gly-Lys(N_3)-TEG-Asp-Glu-Val-Ser-Gly-Leu-Glu-Gln-Leu-Glu-Ser-Ile-Ile-Asn-Phe-Glu-Lys-Leu-Ala-Ala-Ala-Ala-Ala-Lys(sCy5)-NH_2 (21)

Resin bound peptide **20** (5 µmol) was N-terminally acetylated according to the standard conditions. This was followed by chemoselective deprotection of the Mmt group and coupling of sCy5-OH (1.0 eq, 3.2 mg, 5 µmol) as described in the general methods. Global deprotection followed by RP-HPLC purification yielded compound **21** as a blue solid (1.24 mg, 0.33 µmol, 6.6%). **LC-MS** RT = 5.0 min (C18, 10-90% B over 9 minutes) **LRMS** calcd $[M+2H]^{2+}$ = 1883.93, $[M+3H]^{3+}$ = 1256.28 observed M/z = 1884.42, 1256.67

Ac-Lys(N_3)-TEG-Asp(OtBu)-Glu(OtBu)-Val-Ser(tBu)-Gly-Leu-Glu(OtBu)-Gln(Trt)-Leu-Glu(OtBu)-Ser(tBu)-Ile-Ile-Asn(Trt)-Phe-Glu(OtBu)-Lys(Boc)-Leu-Ala-Ala-Ala-Ala-Ala-Lys(Mmt)-RAM-Tentagel S (22)

Resin bound peptide **16** (25 µmol) was elongated manually with Fmoc-TEG-OH, Fmoc-Lys(N_3)-OH and Fmoc-Gly-OH, followed by acetylation under standard conditions. **LC-MS** RT = 4.9 min (C18, 10-90% B over 9 minutes) **LRMS** calcd $[M+2H]^{2+}$ = 1465.77, $[M+3H]^{3+}$ = 977.51 observed M/z = 1466.33, 977.83

Ac-Lys(N_3)-TEG-Asp-Glu-Val-Ser-Gly-Leu-Glu-Gln-Leu-Glu-Ser-Ile-Ile-Asn-Phe-Glu-Lys-Leu-Ala-Ala-Ala-Ala-Ala-Lys(sCy5)-NH_2 (23)

Resin bound peptide **22** (5 µmol) was chemoselectively deprotected to remove the Mmt group, followed by coupling of sCy5-OH (1.0 eq, 3.2 mg, 5 µmol) as described in the general methods. Global deprotection followed by RP-HPLC purification yielded compound **23** as a blue solid (0.88 mg, 0.25 µmol, 4.9%). **LC-MS** RT = 4.7 min (C18, 10-90% B over 9 minutes) **LRMS** calcd $[M+2H]^{2+}$ = 1777.87, $[M+3H]^{3+}$ = 1185.58 observed M/z = 1778.67, 1186.08

Ac-TEG-Asp-Glu-Val-Ser-Gly-Leu-Glu-Gln-Leu-Glu-Ser-Ile-Ile-Asn-Phe-Glu-Lys-Leu-Ala-Ala-Ala-Ala-Ala-Lys(sCy5)-NH_2 (7)

Resin bound peptide **16** (25 µmol) was elongated manually with Fmoc-TEG-OH followed by N-terminal acetylation according to the standard conditions. Next, chemoselective deprotection of the Mmt group and coupling of sCy5-OH (1.0 eq, 3.2 mg, 5 µmol) as described in the general methods yielded the fully protected, resin bound peptide. Global deprotection followed by RP-HPLC purification yielded compound **7** as a blue solid (1.44 mg, 0.42 µmol, 8.5%). **LC-MS** RT = 4.6 min (C18, 10-90% B over 9 minutes) **LRMS** calcd $[M+2H]^{2+}$ = 1701.32, $[M+3H]^{3+}$ = 1134.54 observed M/z = 1701.58, 1134.75

CuAAC glycopeptide conjugation

General procedures for CuAAC modification of peptides

Peptides were dissolved in degassed DMSO and glycans in either degassed DMSO or degassed MilliQ water. These were mixed in a 1.5 mL Eppendorf tube, followed by addition of a copper click mix. This click mix typically consistent of 1 part 0.1 M $CuSO_4$ (in MQ), 2 parts 0.1 M sodium ascorbate (in MQ) and 3 parts 0.1 M THPTA (in DMSO), giving a solution with a total Cu(I) concentration of around 15 mM. Of this solution, enough was added to be 0.15 equivalents compared to the peptide. The reaction was heated in a 40°C shaker block, and every 24 hours 1 equivalent of 0.1 M sodium ascorbate was added. Periodically, a 0.5 µL sample was diluted into 39.5 µL of 1:1:1 H_2O:MeCN:tBuOH and analyzed by LC-MS to evaluate reaction progression. After full conversion of the peptide towards the desired conjugate was observed, the reaction mixture was subjected to size exclusion chromatography over Toyopearl HW-40 size exclusion resin using 150 mM NH_4OAc or 150 mM NH_4HCO_3 (containing 20% MeCN) as the buffer. Fractions showing absorbance at 610 nm were combined and lyophilized.

Ac-Lys(Man)-Lys(Man)-Lys(Man)-Lys(Man)-Lys(Man)-Lys(Man)-TEG-Asp-Glu-Val-Ser-Gly-Leu-Glu-Gln-Leu-Glu-Ser-Ile-Ile-Asn-Phe-Glu-Lys-Leu-Ala-Ala-Ala-Ala-Ala-Lys(sCy5)-NH_2 (3)

Compound **19** (100 nmol, 50 mM in DMSO) was mixed with propargyl mannoside **14** (12 eq., 6 µL of 200 mM solution in DMSO). 1 µL of the standard click mix was added and the reaction carried out at 40°C. After SEC purification and lyophilization compound **3** was obtained as a blue powder (0.48 mg, 85 nmol, 85%). **LC-MS** RT = 4.3 min (C18, 10-50% B over 9 minutes) **LRMS** calcd $[M+3H]^{3+}$ = 1879.21, $[M+4H]^{4+}$ = 1409.66 observed M/z = 1879.33, 1409.83

Ac-Lys(triMan)-Lys(triMan)-Lys(triMan)-Lys(triMan)-Lys(triMan)-Lys(triMan)-TEG-Asp-Glu-Val-Ser-Gly-Leu-Glu-Gln-Leu-Glu-Ser-Ile-Ile-Asn-Phe-Glu-Lys-Leu-Ala-Ala-Ala-Ala-Ala-Lys(sCy5)-NH_2 (6)

Compound **19** (100 nmol, 50 mM in DMSO) was mixed with propargyl mannoside **15** (12 eq., 6 µL of 200 mM solution in DMSO). 1 µL of the standard click mix was added and the reaction carried out at 40°C. After SEC purification and lyophilization compound **6** was obtained as a blue powder (0.41 mg, 54 nmol, 54%). **LC-MS** RT = 4.2 min (C18, 10-50% B over 9 minutes) **LRMS** calcd $[M+4H]^{4+}$ = 1896.07, $[M+5H]^{5+}$ = 1517.06 observed M/z = 1896.08, 1517.17

Ac-Lys(Man)-Gly-Lys(Man)-TEG-Asp-Glu-Val-Ser-Gly-Leu-Glu-Gln-Leu-Glu-Ser-Ile-Ile-Asn-Phe-Glu-Lys-Leu-Ala-Ala-Ala-Ala-Ala-Lys(sCy5)-NH_2 (2)

Compound **21** (100 nmol, 25 mM in DMSO) was mixed with propargyl mannoside **14** (4 eq., 2 µL of 200 mM solution in DMSO). 1 µL of the standard click mix was added and the reaction carried out at 40°C. After SEC purification and lyophilization compound **2** was obtained as a blue powder (0.25 mg, 60 nmol, 60%). **LC-MS** RT = 6.8 min (C18, 10-50% B over 9 minutes) **LRMS** calcd $[M+3H]^{3+}$ = 1402.00 observed M/z = 1402.08

Ac-Lys(triMan)-Gly-Lys(triMan)-TEG-Asp-Glu-Val-Ser-Gly-Leu-Glu-Gln-Leu-Glu-Ser-Ile-Ile-Asn-Phe-Glu-Lys-Leu-Ala-Ala-Ala-Ala-Ala-Lys(sCy5)-NH_2 (5)

Compound **21** (100 nmol, 25 mM in DMSO) was mixed with propargyl mannoside **15** (4 eq., 2 µL of 200 mM solution in DMSO). 1 µL of the standard click mix was added and the reaction carried out at 40°C. After SEC purification and lyophilization compound **5** was obtained as a blue powder (0.29 mg, 60 nmol, 60%). **LC-MS** RT = 6.6 min (C18, 10-50% B over 9 minutes) **LRMS** calcd $[M+3H]^{3+}$ = 1618.07 observed M/z = 1618.08

Ac-Lys(Man)-TEG-Asp-Glu-Val-Ser-Gly-Leu-Glu-Gln-Leu-Glu-Ser-Ile-Ile-Asn-Phe-Glu-Lys-Leu-Ala-Ala-Ala-Ala-Ala-Lys(sCy5)-NH2 (1)

Compound **23** (100 nmol, 25 mM in DMSO) was mixed with propargyl mannoside **14** (2 eq., 1 µL of 200 mM solution in DMSO). 1 µL of the standard click mix was added and the reaction carried out at 40°C. After SEC purification and lyophilization compound **1** was obtained as a blue powder (0.30 mg, 79 nmol, 79%). **LC-MS** RT = 4.4 min (C18, 10-90% B over 9 minutes) **LRMS** calcd $[M+2H]^{2+}$ = 1886.91, $[M+3H]^{3+}$ = 1258.27 observed M/z = 1887.33, 1258.75

Ac-Lys(triMan)-TEG-Asp-Glu-Val-Ser-Gly-Leu-Glu-Gln-Leu-Glu-Ser-Ile-Ile-Asn-Phe-Glu-Lys-Leu-Ala-Ala-Ala-Ala-Ala-Lys(sCy5)-NH_2 (4)

Compound **23** (100 nmol, 25 mM in DMSO) was mixed with propargyl mannoside **15** (2 eq., 1 µL of 200 mM solution in DMSO). 1 µL of the standard click mix was added and the reaction carried out at 40°C. After SEC purification and lyophilization compound **4** was obtained as a blue powder (0.26 mg, 63 nmol, 63%). **LC-MS** RT = 4.4 min (C18, 10-90% B over 9 minutes) **LRMS** calcd $[M+3H]^{3+}$ = 1366.31 observed M/z = 1366.75

LC-MS analysis

Ac-Lys(N_3)-Lys(N_3)-Lys(N_3)-Lys(N_3)-Lys(N_3)-Lys(N_3)-TEG-Asp-Glu-Val-Ser-Gly-Leu-Glu-Gln-Leu-Glu-Ser-Ile-Ile-Asn-Phe-Glu-Lys-Leu-Ala-Ala-Ala-Ala-Ala-Lys(sCy5)-NH_2 (19)

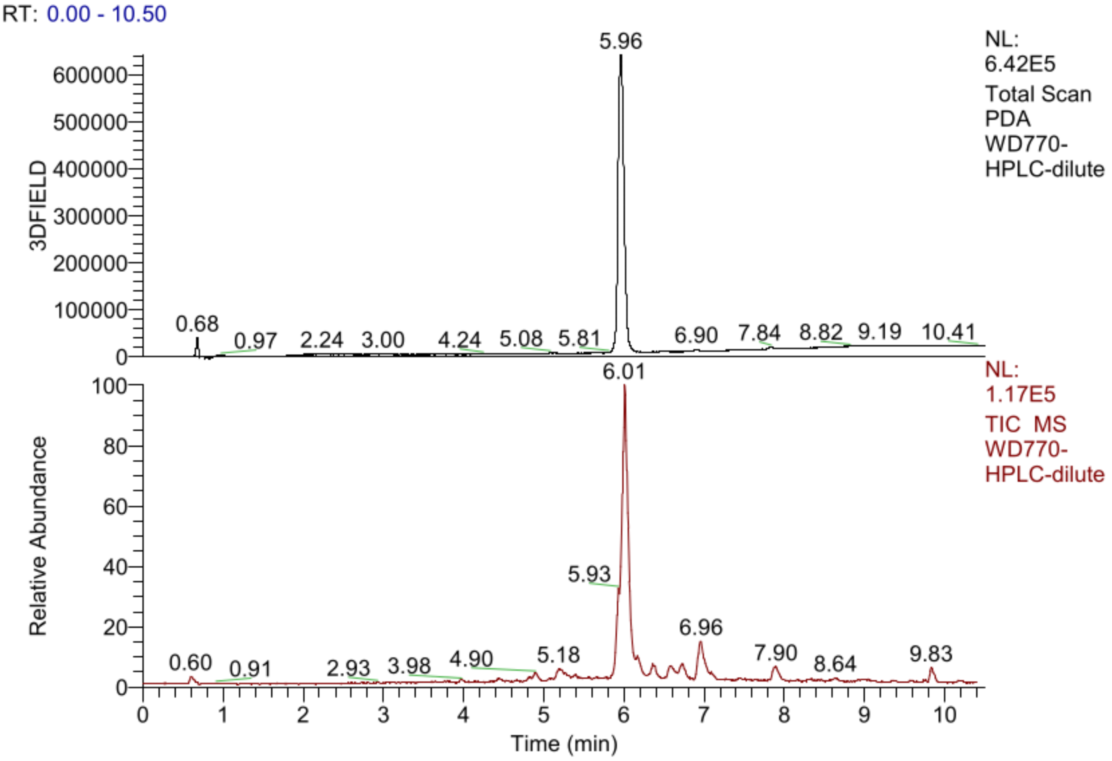

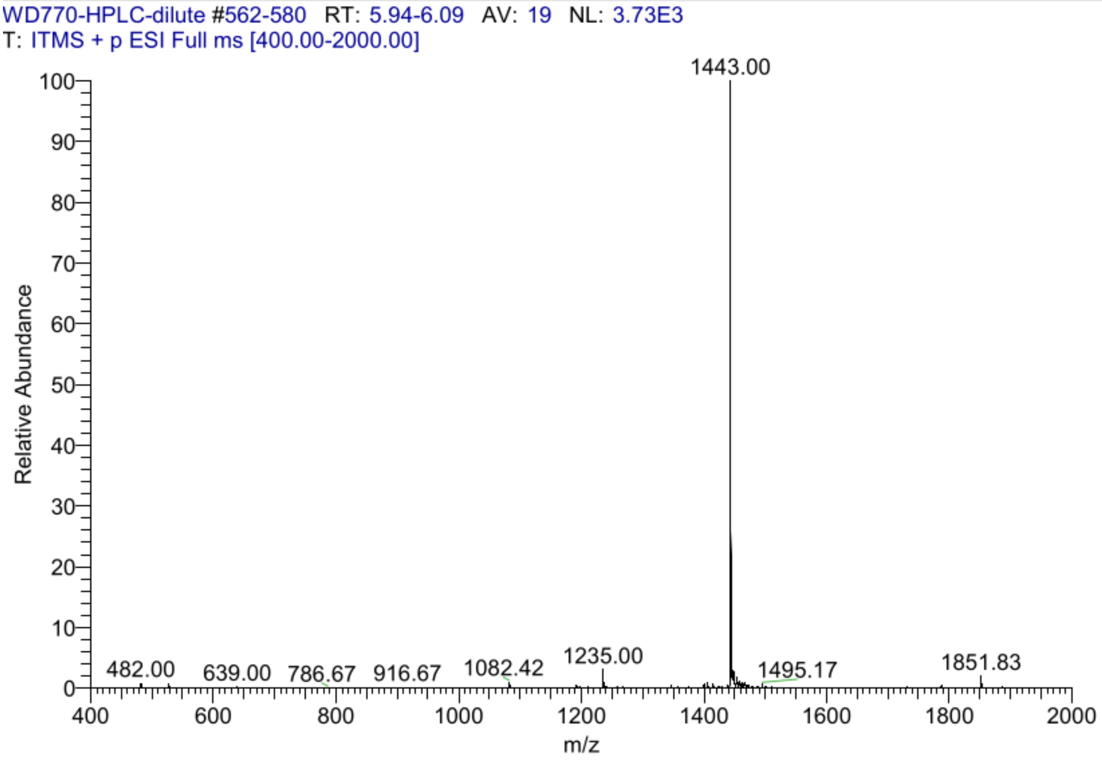

Ac-Lys(N_3)-Gly-Lys(N_3)-TEG-Asp-Glu-Val-Ser-Gly-Leu-Glu-Gln-Leu-Glu-Ser-Ile-Ile-Asn-Phe-Glu-Lys-Leu-Ala-Ala-Ala-Ala-Ala-Lys(sCy5)-NH_2 (21)

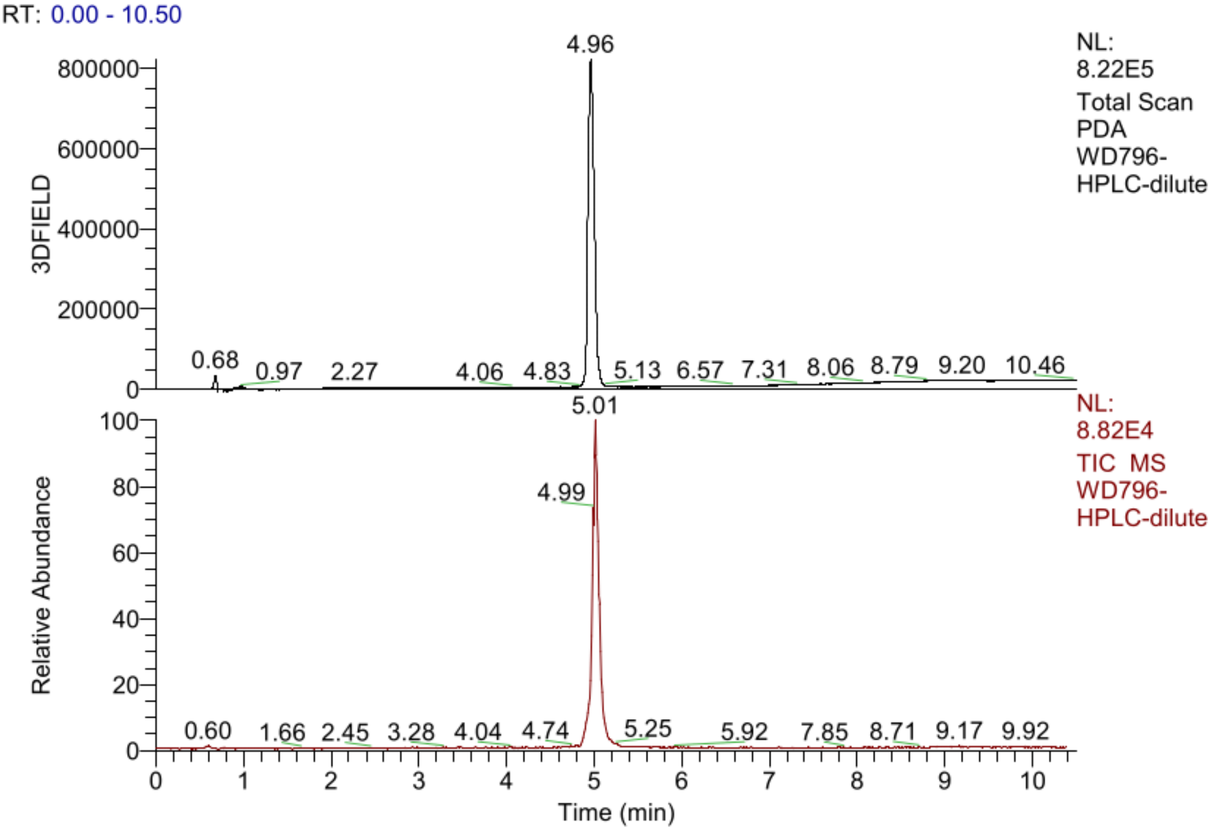

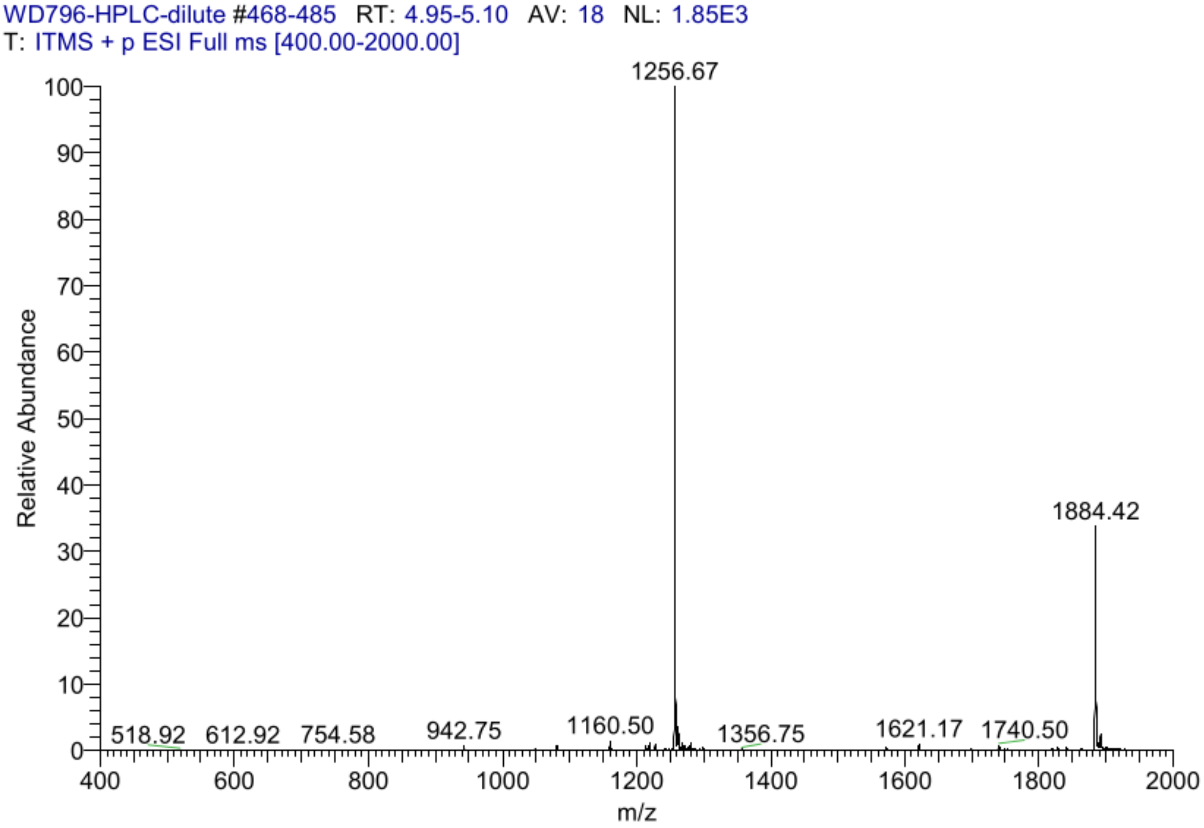

Ac-Lys(N_3)-TEG-Asp-Glu-Val-Ser-Gly-Leu-Glu-Gln-Leu-Glu-Ser-Ile-Ile-Asn-Phe-Glu-Lys-Leu-Ala-Ala-Ala-Ala-Ala-Lys(sCy5)-NH_2 (23)

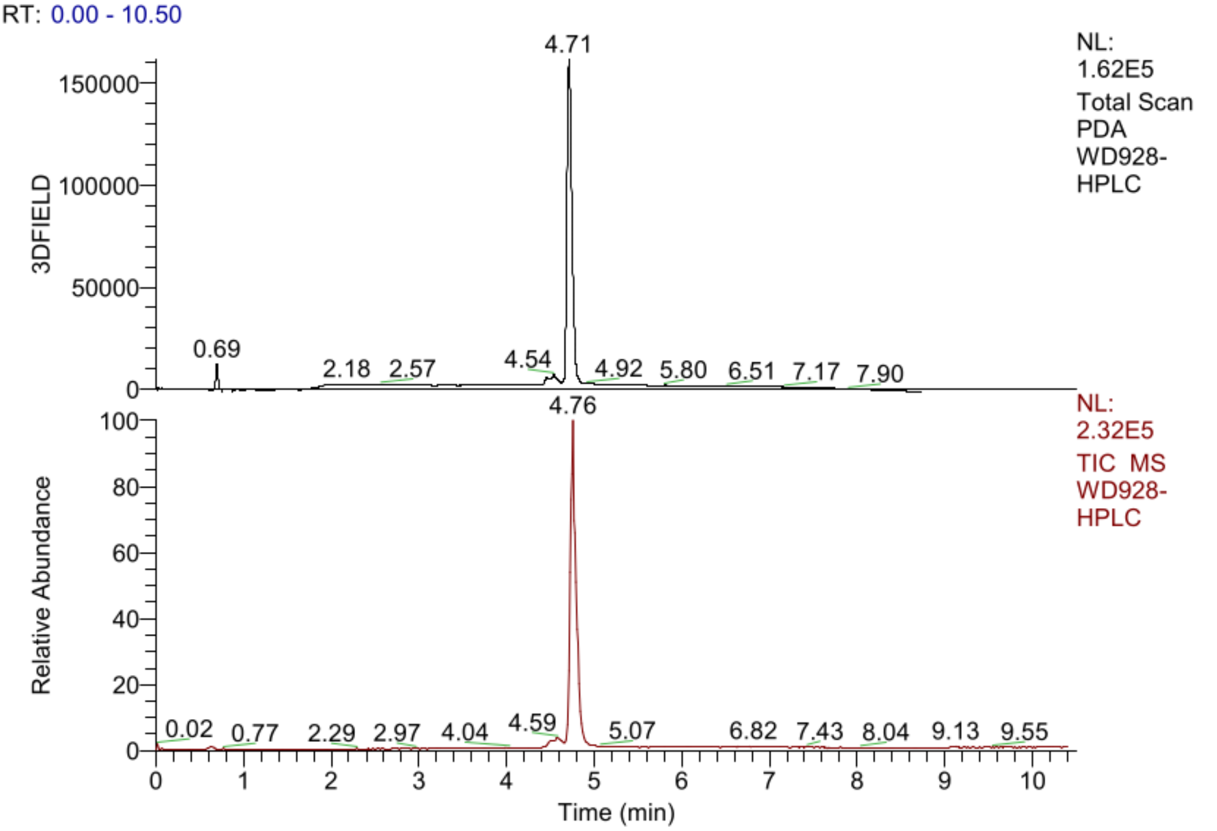

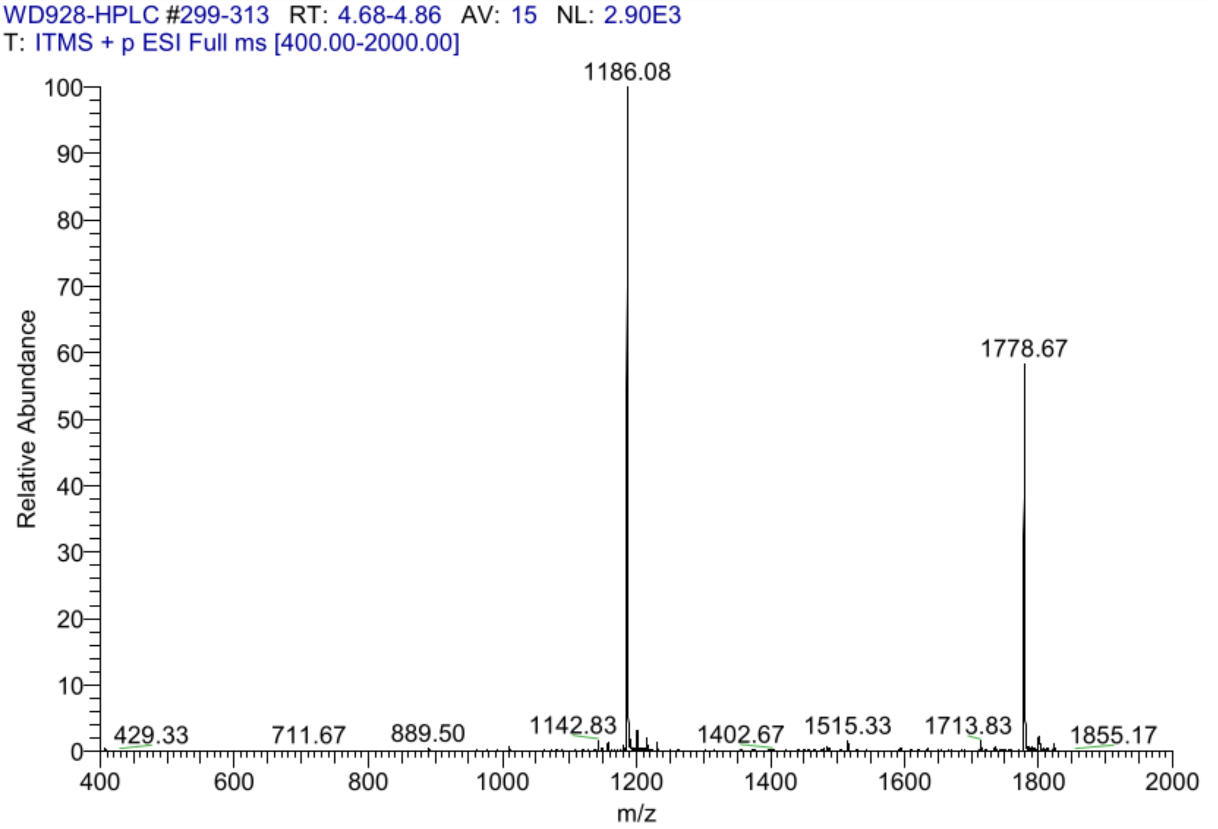

Ac-Lys(Man)-Lys(Man)-Lys(Man)-Lys(Man)-Lys(Man)-Lys(Man)-TEG-Asp-Glu-Val-Ser-Gly-Leu-Glu-Gln-Leu-Glu-Ser-Ile-Ile-Asn-Phe-Glu-Lys-Leu-Ala-Ala-Ala-Ala-Ala-Lys(sCy5)-NH_2 (3)

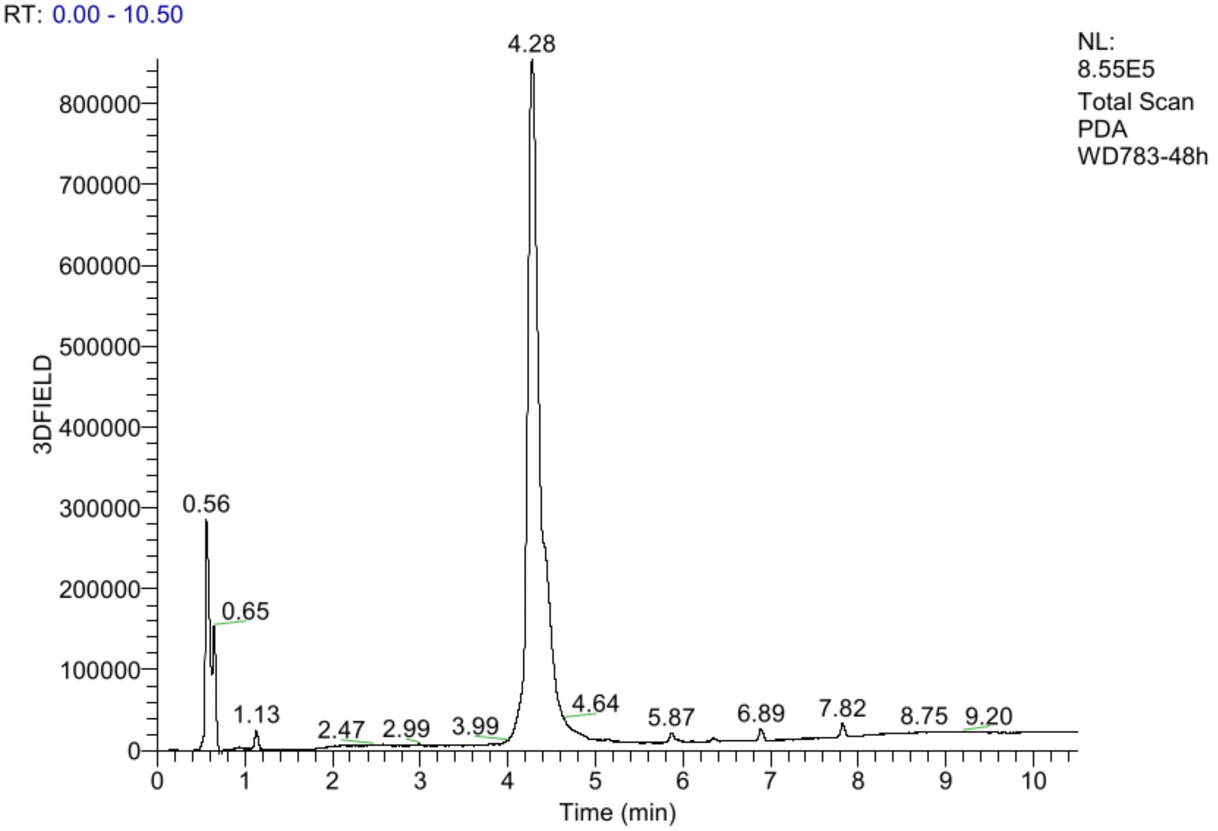

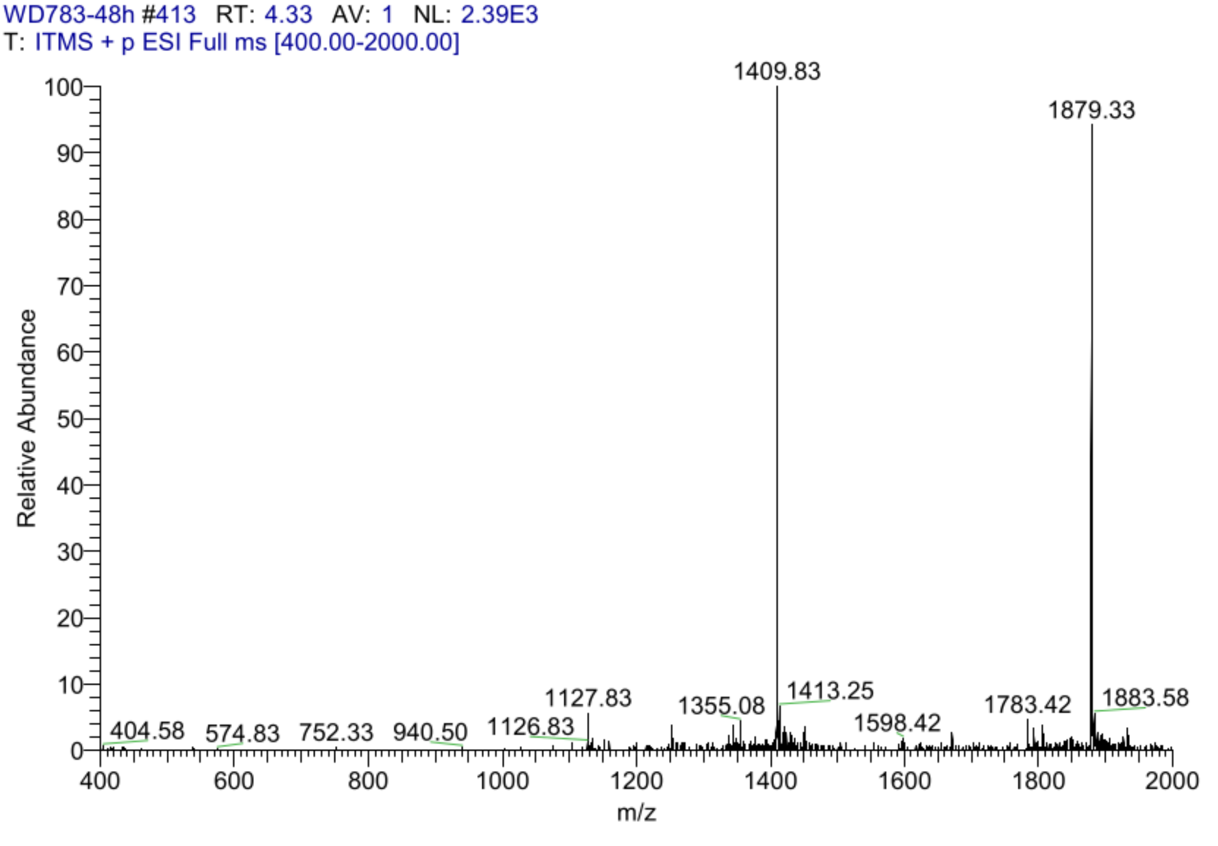

Ac-Lys(triMan)-Lys(triMan)-Lys(triMan)-Lys(triMan)-Lys(triMan)-Lys(triMan)-TEG-Asp-Glu-Val-Ser-Gly-Leu-Glu-Gln-Leu-Glu-Ser-Ile-Ile-Asn-Phe-Glu-Lys-Leu-Ala-Ala-Ala-Ala-Ala-Lys(sCy5)-NH$_2$ (6)

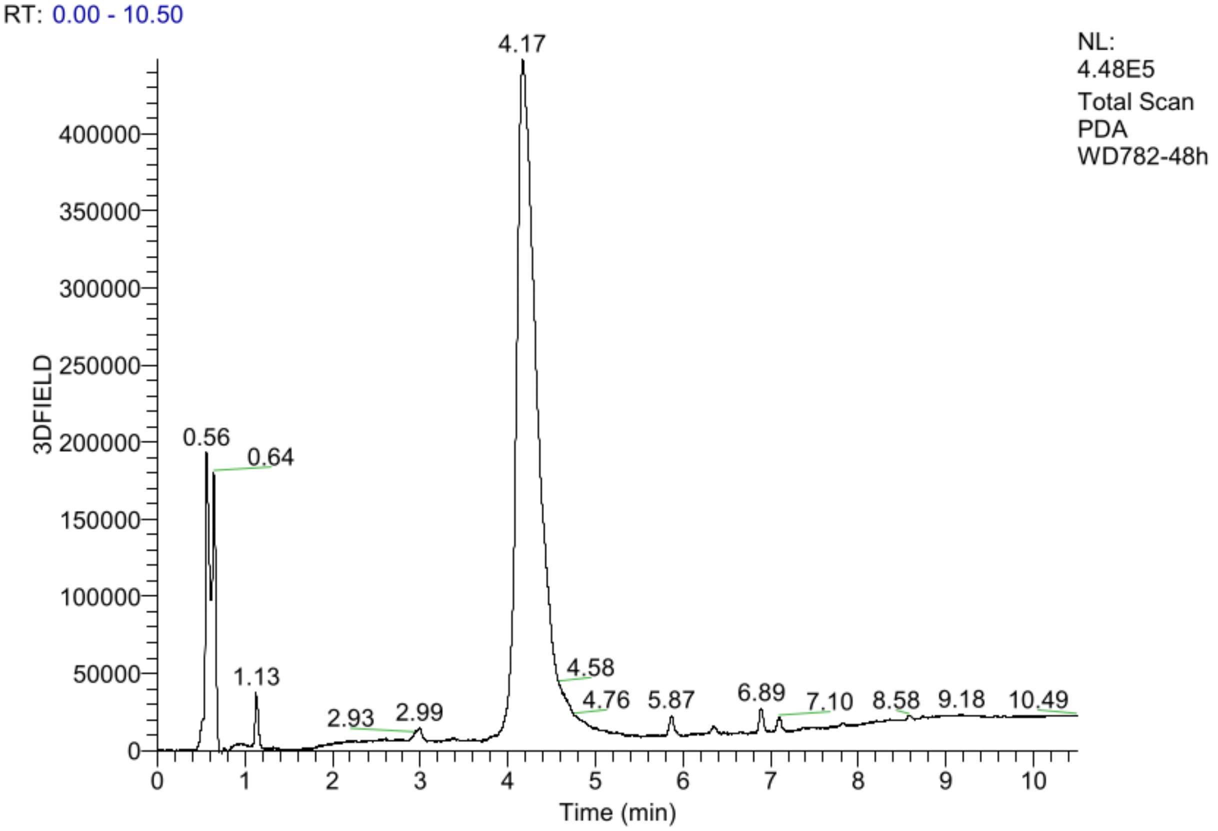

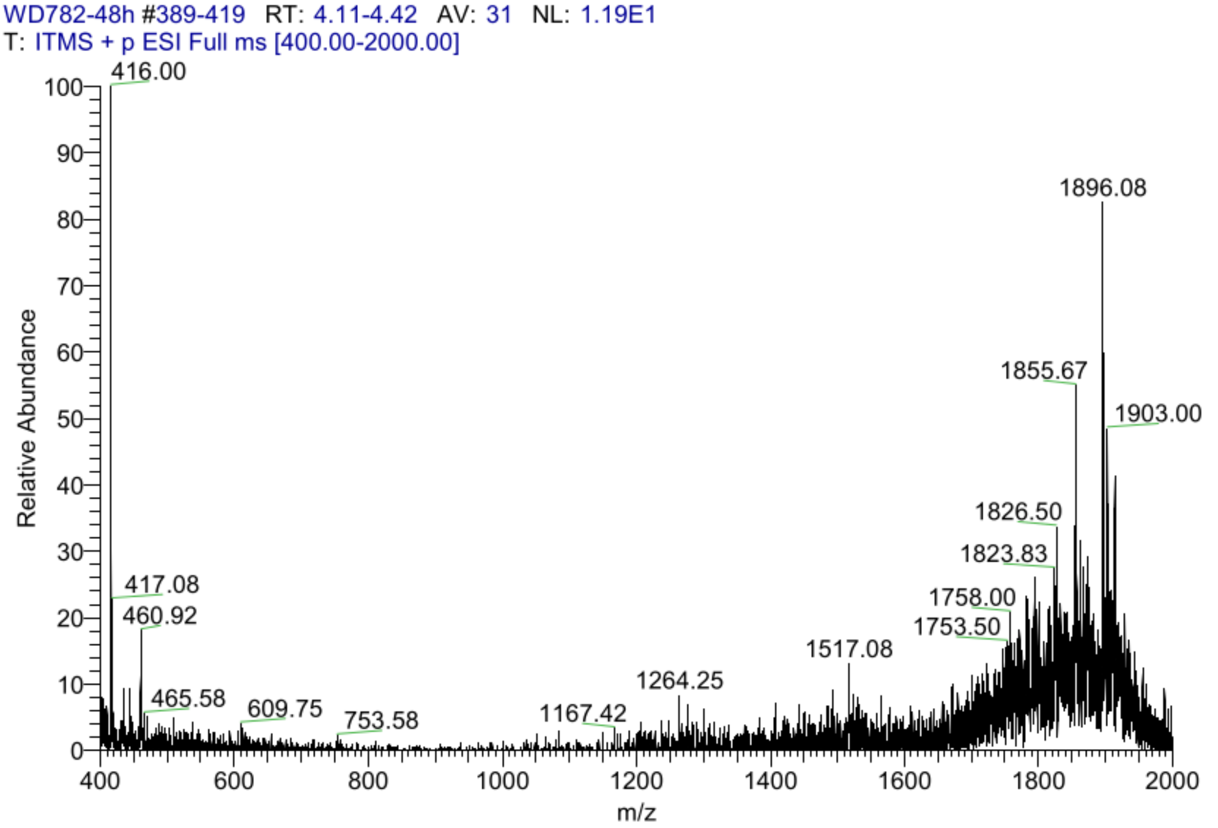

Ac-Lys(Man)-Gly-Lys(Man)-TEG-Asp-Glu-Val-Ser-Gly-Leu-Glu-Gln-Leu-Glu-Ser-Ile-Ile-Asn-Phe-Glu-Lys-Leu-Ala-Ala-Ala-Ala-Ala-Lys(sCy5)-NH_2 (2)

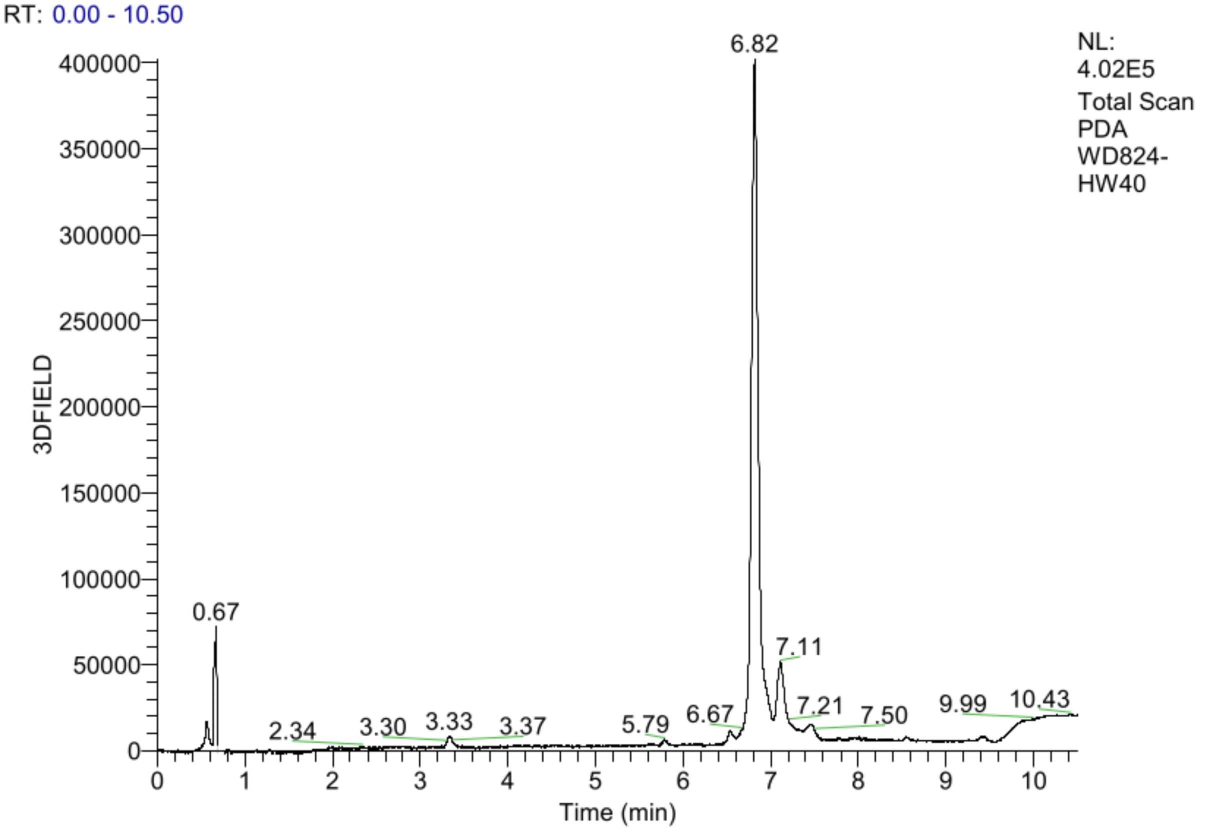

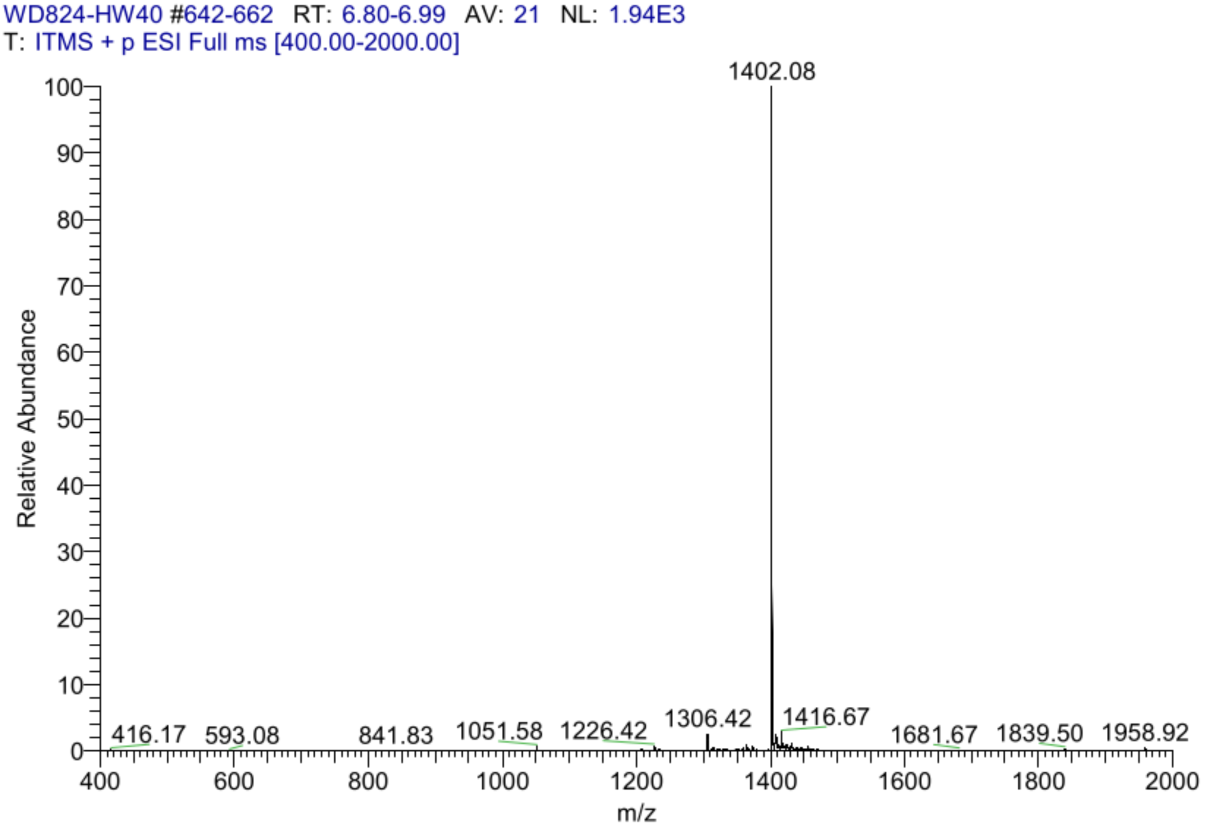

Ac-Lys(triMan)-Gly-Lys(triMan)-TEG-Asp-Glu-Val-Ser-Gly-Leu-Glu-Gln-Leu-Glu-Ser-Ile-Ile-Asn-Phe-Glu-Lys-Leu-Ala-Ala-Ala-Ala-Ala-Lys(sCy5)-NH_2 (5)

RT: 0.00 - 10.50

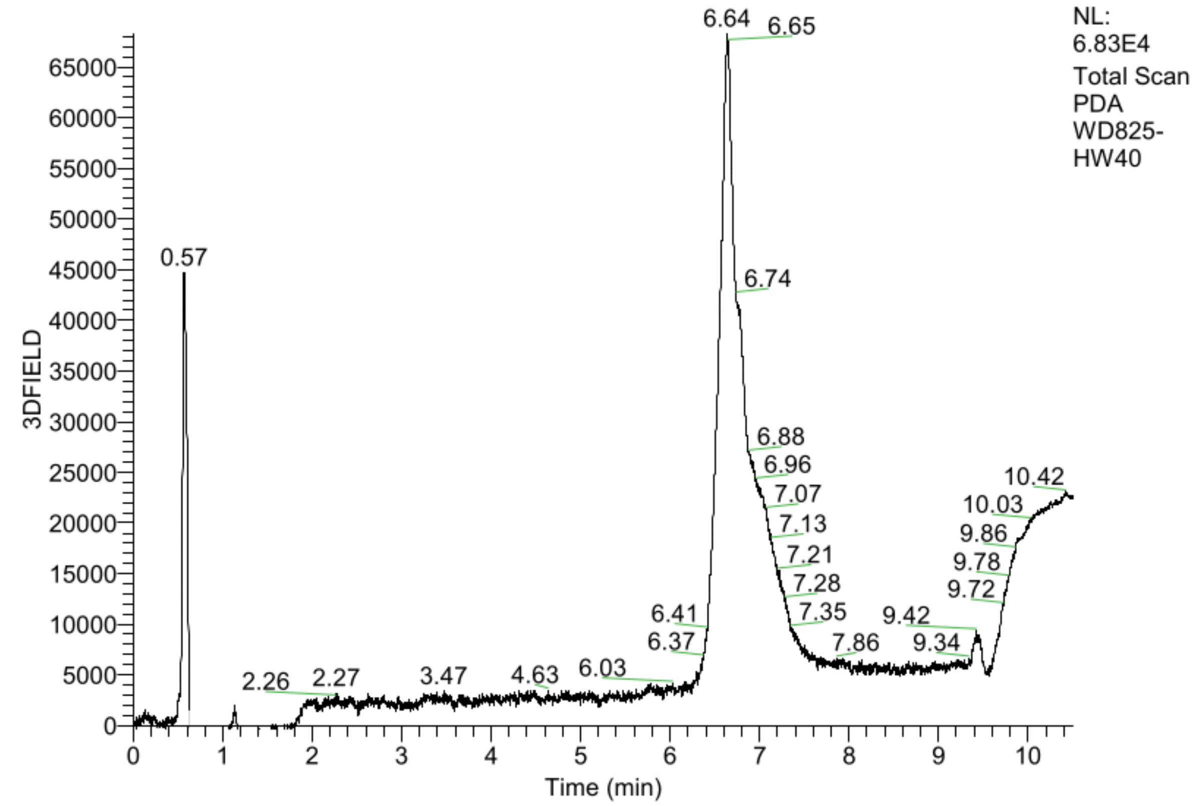

WD825-HW40 #633 RT: 6.70 AV: 1 NL: 1.96E2

T: ITMS + p ESI Full ms [400.00-2000.00]

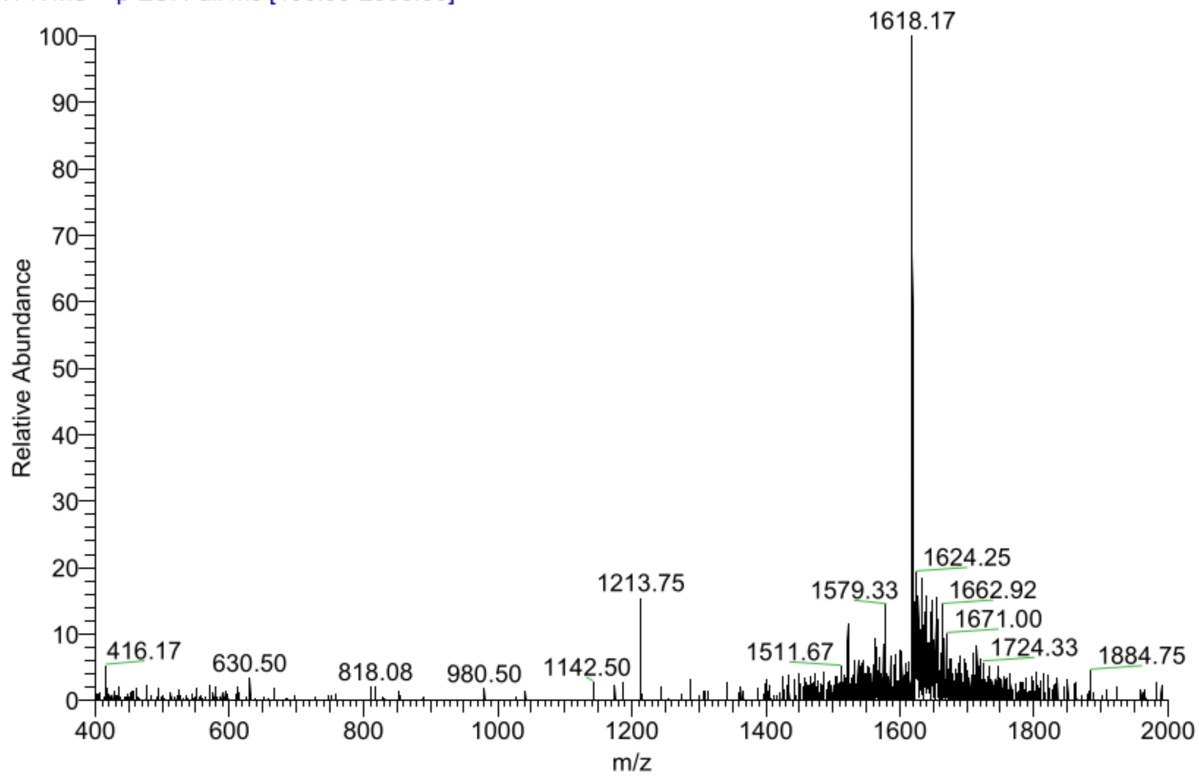

Ac-Lys(Man)-TEG-Asp-Glu-Val-Ser-Gly-Leu-Glu-Gln-Leu-Glu-Ser-Ile-Ile-Asn-Phe-Glu-Lys-Leu-Ala-Ala-Ala-Ala-Ala-Lys(sCy5)-NH_2 (1)

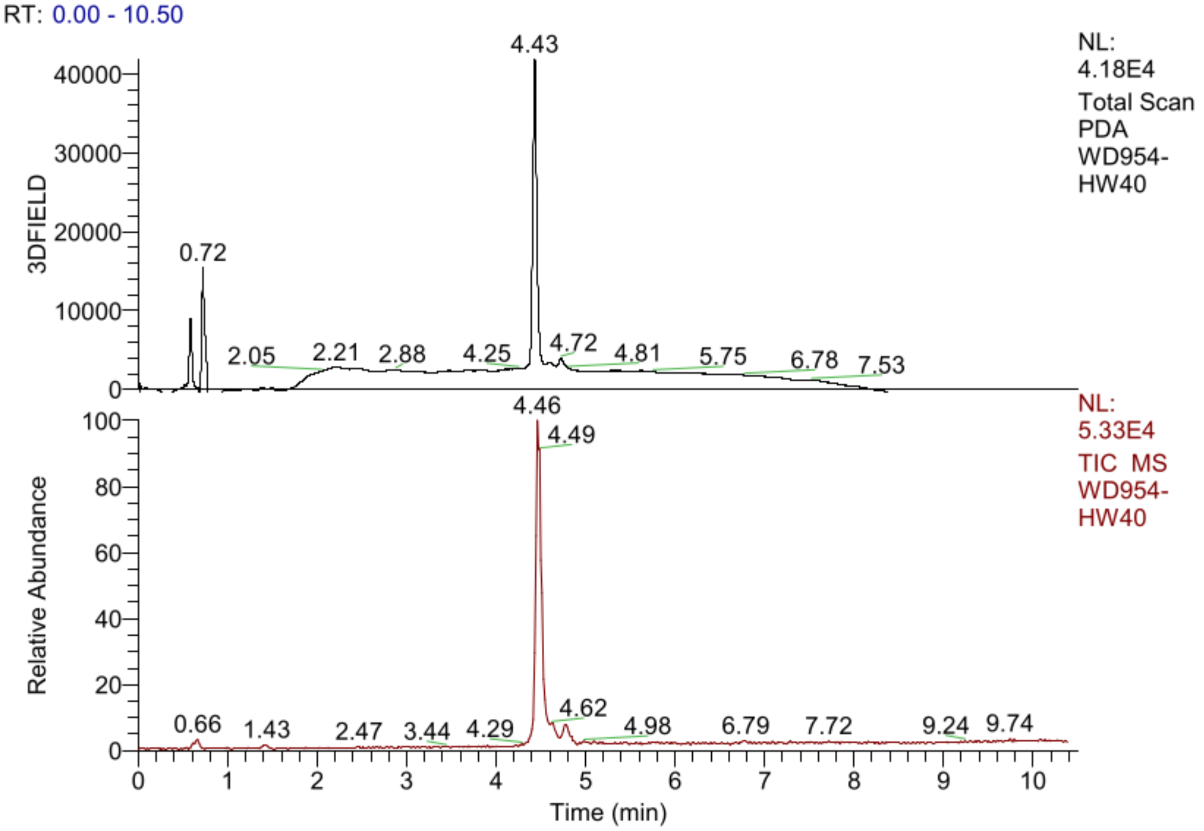

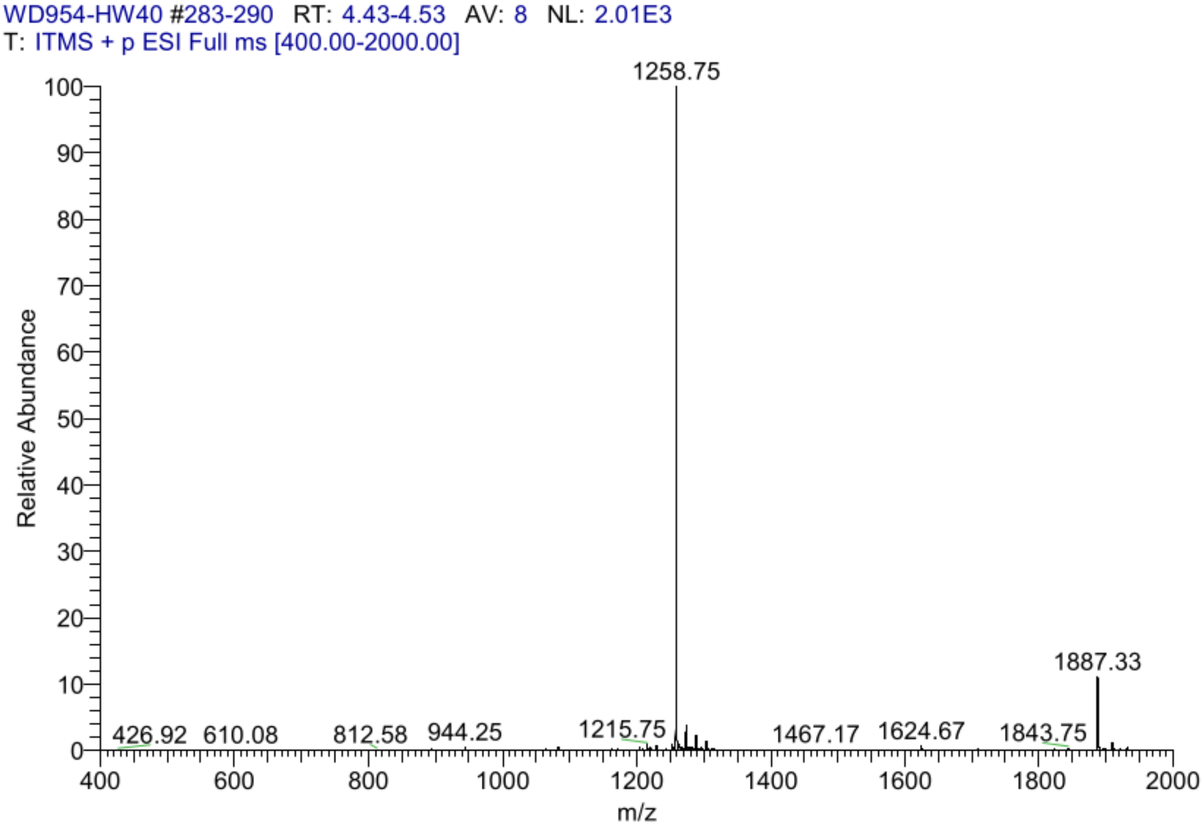

Ac-Lys(triMan)-TEG-Asp-Glu-Val-Ser-Gly-Leu-Glu-Gln-Leu-Glu-Ser-Ile-Ile-Asn-Phe-Glu-Lys-Leu-Ala-Ala-Ala-Ala-Ala-Lys(sCy5)-NH_2 (4)

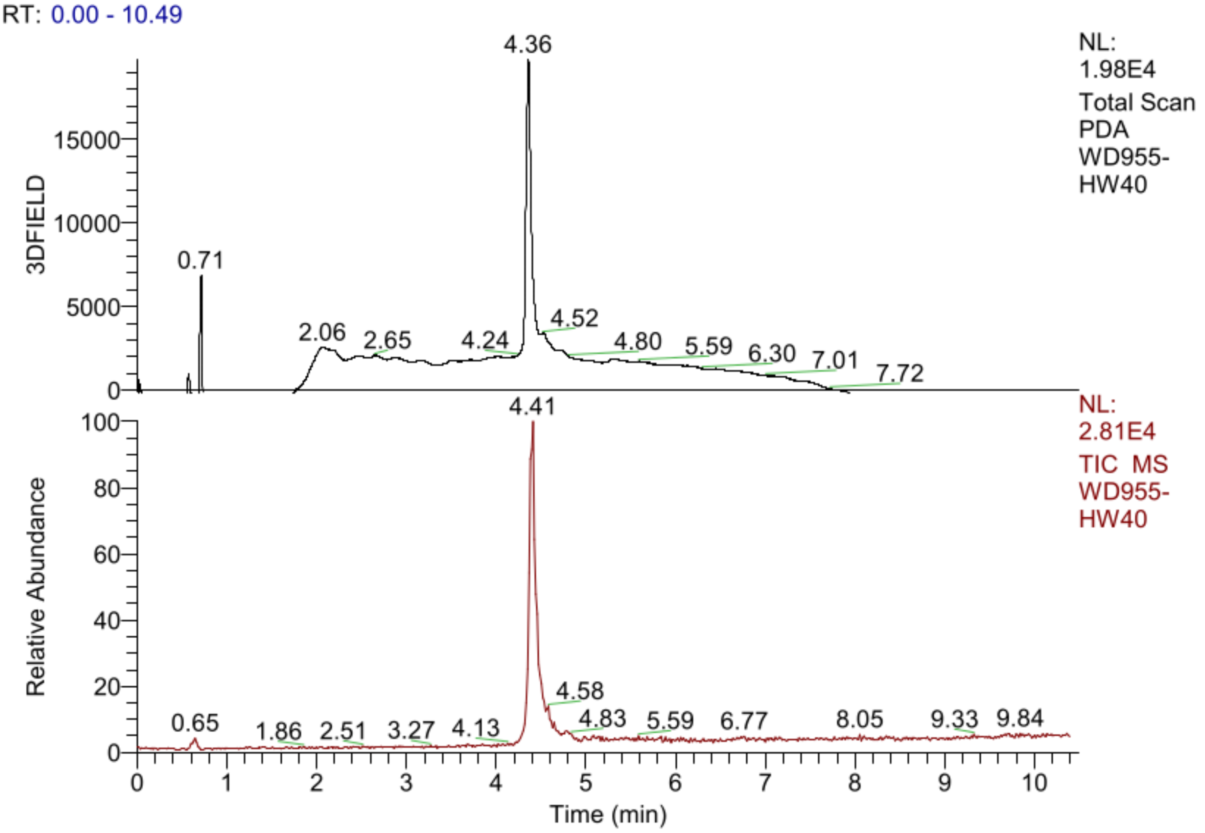

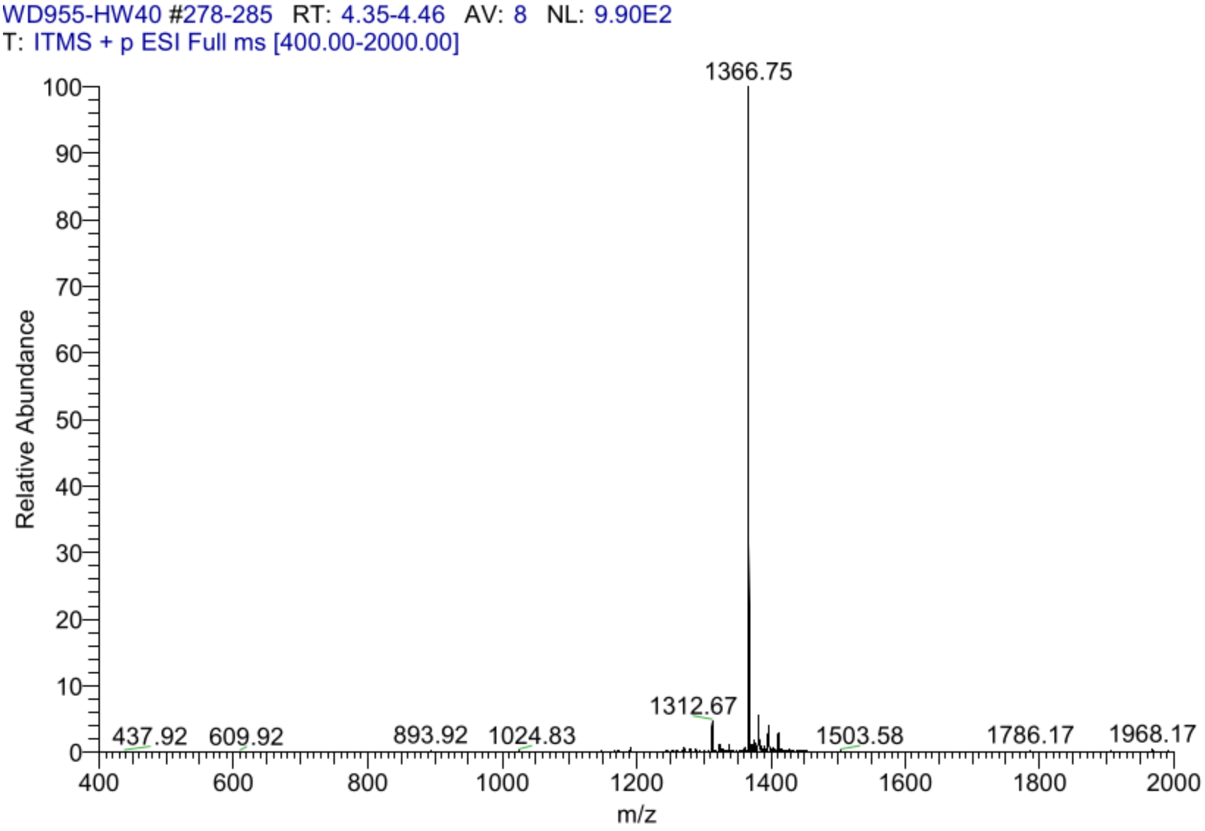

SUPPLEMENTARY REFERENCES

(1) Riera, R.; Hogervorst, T. P.; Doelman, W.; Ni, Y.; Pujals, S.; Bolli, E.; Codée, J. D. C.; van Kasteren, S. I.; Albertazzi, L. Single-Molecule Imaging of Glycan–Lectin Interactions on Cells with Glyco-PAINT. *Nat Chem Biol* **2021**, *17* (12), 1281–1288. https://doi.org/10.1038/s41589-021-00896-2.

(2) Li, R.-J. E.; Hogervorst, T. P.; Achilli, S.; Bruijns, S. C.; Arnoldus, T.; Vivès, C.; Wong, C. C.; Thépaut, M.; Meeuwenoord, N. J.; van den Elst, H.; Overkleeft, H. S.; van der Marel, G. A.; Filippov, D. V.; van Vliet, S. J.; Fieschi, F.; Codée, J. D. C.; van Kooyk, Y. Systematic Dual Targeting of Dendritic Cell C-Type Lectin Receptor DC-SIGN and TLR7 Using a Trifunctional Mannosylated Antigen. *Front. Chem.* **2019**, *7*, 650. https://doi.org/10.3389/fchem.2019.00650.

(3) Aletras, A.; Barlos, K.; Gatos, D.; Koutsogianni, S.; MAMOS, P. Preparation of the Very Acid-sensitive Fmoc-Lys(Mtt)-OH Application in the Synthesis of Side-chain to Side-chain Cyclic Peptides and Oligolysine Cores Suitable for the Solid-phase Assembly of MAPs and TASPs. *International Journal of Peptide and Protein Research* **1995**, *45* (5), 488–496. https://doi.org/10.1111/j.1399-3011.1995.tb01065.x.

For the latest version of this document refer to GitHub (https://github.com/Leiden-chemical-immunology/Glyco-PAINT/tree/main/doc)

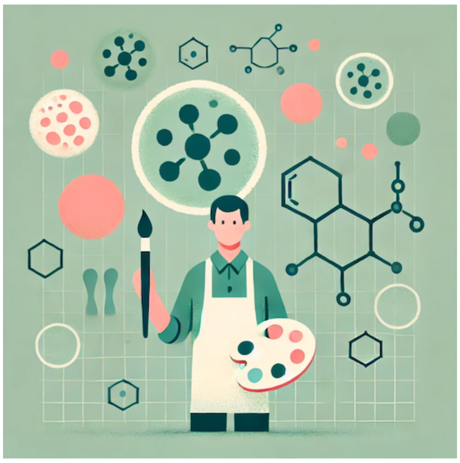

Glyco-PAINT Automated Processing Pipeline

Supplementary Software Manual

Nov 2025

Introduction

This manual serves two distinct purposes. Firstly, it provides a comprehensive background on the Glyco-PAINT-Automated Processing Pipeline. Secondly, it introduces a demonstration case to help users familiarise themselves with the pipeline's functionality. Detailed instructions for running the example are provided in Running the Example.

In the Glyco-PAINT method, cells incubated with a fluorescent ligand are imaged using a specialised microscope for 100 seconds, during which 2000 frames are captured. Bright spots - corresponding to stationary (i.e., bound) fluorophores - appear in these frames. These spots represent ligands that have remained immobilised long enough to be detected by the camera and are interpreted as glycans (or other ligands) binding to receptors on the cell surface. Fluorophores may move or disappear shortly after appearing, reflecting unbinding or photobleaching events. A key challenge in this method is distinguishing true biologically relevant binding events from non-specific sticking of fluorophores to the cell surface or coverslip.

The Glyco-PAINT pipeline addresses this challenge in two main steps:

Spot Detection and Tracking

The Fiji plugin TrackMate detects spots in each frame and records their coordinates. Tracks are created by linking spots across consecutive frames. Each track is characterised by its duration, average x/y position, and distance travelled. If a receptor-ligand pair were completely stationary, it would appear as a series of overlapping spots at the same location. In reality, ligand-receptor complexes typically exhibit slight lateral motion within the membrane, resulting in short, “wriggling” tracks (see illustration). These tracks are typically concentrated on the cell surface and display heterogeneous spatial distributions within individual cells.

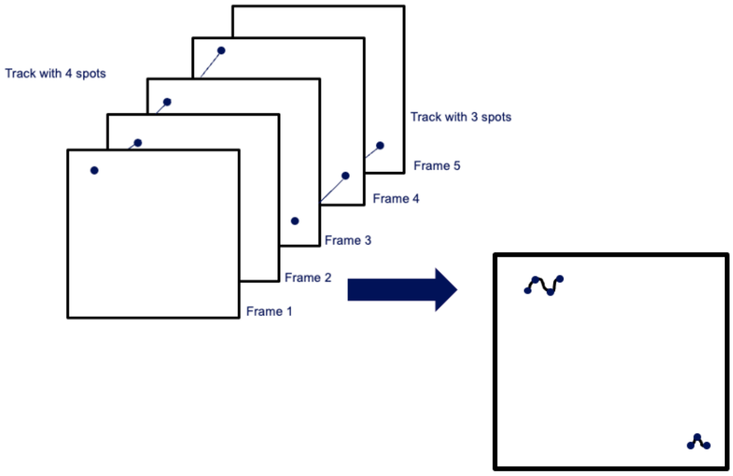

Grid-Based Spatial Analysis

To account for spatial segregation and background signal filtering, a grid, typically 20x20 or 30x30, is overlaid on each recording. This divides the image into 400 or 900 equally sized squares, enabling analysis of spatial differences across regions within a cell or between

different cells. Summary statistics, such as event density, dwell time, and diffusion, are computed per grid square.

Data Structure

In Glyco-PAINT, the primary unit of analysis is the Experiment, rather than an individual recording. An Experiment is a group of recordings acquired under identical conditions, capturing a specific biological phenotype. Data processing of these recordings generates two main output files:

- All Squares.csv: This file contains summary statistics for each grid square across all recordings in the experiment.
- All Tracks.csv: This file provides detailed tracking information for every individual track in the dataset.

In a typical analysis, as demonstrated in one of the demo experiments, large volumes of data are generated. For instance, each recording produces approximately 800,000 detected spots, resulting in around 50,000 reconstructed tracks. A 20×20 grid across 14 recordings generates 5,600 rows in All Squares.csv, while a 30×30 grid yields 12,600 rows. The corresponding All Tracks.csv file contains approximately 180,000 tracks.

Design Philosophy

Glyco-PAINT is designed to:

- Extract the **maximum amount of biologically meaningful information** from large microscopy datasets.
- Ensure **full reproducibility** of results.
- Require **minimal user intervention.**
- Be **efficient and scalable** in terms of processing time and computational resources.

Pipeline Concepts

The Paint Pipeline was developed to analyse (bulk) data from Glyco-PAINT experiments. The interaction of weak-binding ligands to receptors on live cells is studied on various cell types, ligands and adjuvants. Binding events show up as bright spots under a fluorescent microscope. Analysis of the location and duration of spots reveals information about single binding events. Below are listed some crucial concepts and their definition.

Recording

Glyco-PAINT's central concept revolves around recording binding events using a fluorescence excitation laser. Each recording is a multi-frame image that captures spots indicative of binding. In our experiments, we observe cells for 100 seconds, capturing 2,000 frames at 50-millisecond intervals.

Brightfield image

Each recording is accompanied by a brightfield image that captures the same field-of-view using transmitted white light illumination. This image helps localise cells from the coverslip background. The brightfield image must be named identically to its corresponding recording, with the suffix '-BF'.

Replicate

A recording of a specific experimental condition is repeated within the same experiment (date) and usually in the same well of a microscopy slide, but in a different field-of-view.

Experiment

A set of recordings and brightfield images is referred to as an experiment. Typically, an experiment is conducted in one day, with up to 100 individual recordings. Generally, several conditions are studied in an experiment, and several replicates are recorded for each condition.

Project

A project is a collection of experiments that are analysed together. These experiments may be replicates of earlier ones or introduce new combinations of cell types, probes, or adjuvants.

Metadata

Metadata must be provided for recordings for the Glyco-PAINT pipeline to work. Additionally, a simple convention for naming the recordings is used. For instance, from the name '240116-Exp-1-A1-1', the following information can be derived:

- 240116-Exp-1-A1-1: Part of experiment 240116
- 240116-Exp-1-A1-1: Operating condition 1
- 240116-Exp-1-A1-1: Microscopic plate well A1
- 240116-Exp-1-A1-1: Replicate 1 with the same experimental conditions
- 240116-Exp-1-A1-1-BF: Filename of the brightfield image corresponding to this recording

Process Flow

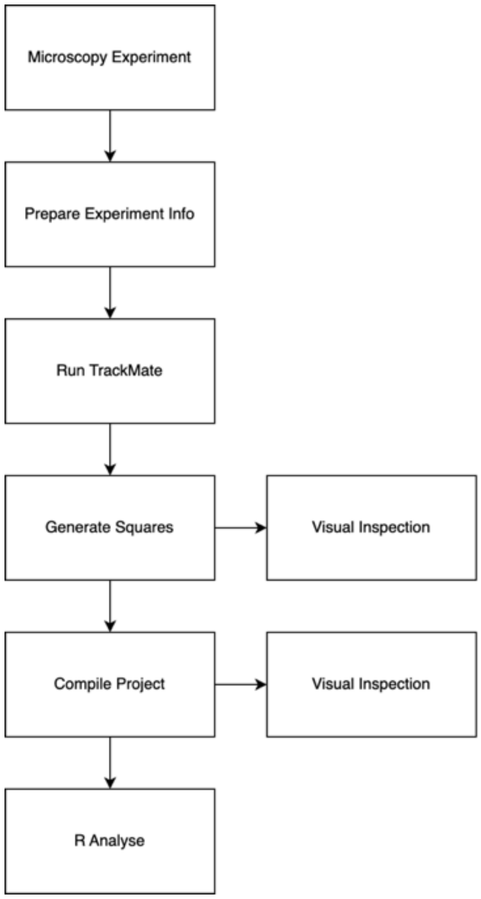

The Glyco-PAINT process begins with a **microscopy experiment** using a specialised fluorescent microscope to observe binding events. This experiment generates recordings and brightfield images for each experimental condition.

The user then prepares the **Experiment Info** by providing metadata for the recordings. This metadata includes properties such as Cell Type, Probe Type, Concentration, Adjuvant, and necessary processing parameters.

Next, the experiment data is analysed to detect spots in each frame of the multi-frame set and connect these spots into tracks where appropriate. A track represents a binding event from which kinetic parameters can be deduced, but background signal filtering is required.

With the tracks available, the **Generate Squares** step overlays a fine grid of squares over each recording, specifying the properties of each square, such as whether it covers an area of interest or background.

Finally, the **Compile Project** combines all data from multiple Experiments into large data tables containing all relevant information, ready for final analysis.

These tables are then used in the **R Analysis** phase to select, process, and present the data in line with research objectives.

The **Recording Viewer** allows users to visually inspect the tracking results, providing feedback on the selection criteria for squares per individual recording. The tracking results are overlayed with the grid and displayed next to the corresponding brightfield image.

All steps will be described in detail in the coming sections, using demo data that can be downloaded from: [Zenodo]

Prepare Experiment Info

The metadata of an experiment is information about the conditions under which each recording was made. This information is stored in an 'Experiment Info' file. The pipeline contains a 'Prepare Experiment Info' utility that prepares this file. The user provides two parameters: the directory containing the raw data (recordings and brightfield images) and the location of the Paint Experiment directory where the 'Experiment Info.csv' file (and all subsequent data) will be written.

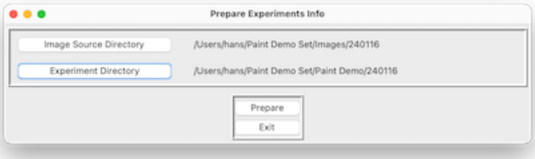

After this has been done for both demo experiments, two directories have been created, each with an 'Experiment Info.csv' file.

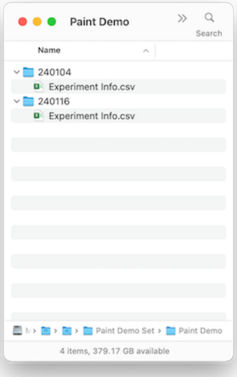

If the file naming convention (as specified above) is used, columns such as Experiment Date, Experiment Name, Condition Nr and Replicate Nr will be filled in automatically. If not, the user must provide this information manually.

Rec. Name	Exp. Date	Exp. Name	Cond. Nr	Rep. Nr	Probe	Probe Type	Cell Type	Adj	Conc.	Thesh.	Process
240116-Exp-1-A1-2	240116	240116	1	2							Yes
240116-Exp-1-A1-3	240116	240116	1	3							Yes
240116-Exp-1-A1-5	240116	240116	1	5							Yes
240116-Exp-2-A2-2	240116	240116	2	2							Yes
240116-Exp-3-A3-2	240116	240116	3	2							Yes
240116-Exp-7-A1-2	240116	240116	7	2							Yes
240116-Exp-7-A1-4	240116	240116	7	4							Yes
240116-Exp-8-A2-2	240116	240116	8	2							Yes
240116-Exp-10-B1-1	240116	240116	10	1							Yes
240116-Exp-10-B1-2	240116	240116	10	2							Yes
240116-Exp-10-B1-3	240116	240116	10	3							Yes
240116-Exp-11-B2-2	240116	240116	11	2							Yes
240116-Exp-12-B3-2	240116	240116	12	2							Yes
240116-Exp-13-A4-2	240116	240116	13	2							Yes

Below is an example of a fully specified Experiment Info file, containing values for Probe, Probe Type, Cell Type, Adjuvant, and Concentration. The Threshold parameter is required for TrackMate processing (refer to the next section). The ‘Process’ flag indicates whether a recording is processed or ignored. You can download copies of these files, 240104 Experiment Info and 240116 Experiment Info, and save them in their respective directories as ‘Experiment Info.csv’.

Seq. Nr	Rec. Name	Exp. Date	Exp. Name	Cond. Nr	Rep. Nr	Probe	Probe Type	Cell Type	Adj	Conc.	Thesh.	Process
1	240116-Exp-1-A1-2	240116	240116	1	2	6 Mono	Simple	BMDC	No	10	5	Yes
2	240116-Exp-1-A1-3	240116	240116	1	3	6 Mono	Simple	BMDC	No	10	10	Yes
3	240116-Exp-1-A1-5	240116	240116	1	5	6 Mono	Simple	BMDC	No	10	10	Yes
4	240116-Exp-2-A2-2	240116	240116	2	2	1 Tri	Simple	BMDC	No	10	10	Yes
5	240116-Exp-3-A3-2	240116	240116	3	2	6 Tri	Simple	BMDC	No	10	10	Yes
6	240116-Exp-7-A1-2	240116	240116	7	2	6 Mono	Simple	BMDC	MPLA	10	20	Yes
7	240116-Exp-7-A1-4	240116	240116	7	4	6 Mono	Simple	BMDC	MPLA	10	10	Yes
8	240116-Exp-8-A2-2	240116	240116	8	2	1 Tri	Simple	BMDC	MPLA	10	10	Yes
9	240116-Exp-10-B1-1	240116	240116	10	1	6 Mono	Simple	iCD103	MPLA	10	20	Yes
10	240116-Exp-10-B1-2	240116	240116	10	2	6 Mono	Simple	iCD103	MPLA	10	10	Yes
11	240116-Exp-10-B1-3	240116	240116	10	3	6 Mono	Simple	iCD103	MPLA	10	10	Yes
12	240116-Exp-11-B2-2	240116	240116	11	2	1 Tri	Simple	iCD103	MPLA	10	10	Yes
13	240116-Exp-12-B3-2	240116	240116	12	2	6 Tri	Simple	iCD103	MPLA	10	20	Yes
14	240116-Exp-13-A4-2	240116	240116	13	2	6 Tri	Simple	BMDC	LPS	10	20	Yes

Run TrackMate

The TrackMate plugin in Fiji analyses recordings, detects spots, and connects them to form tracks where possible. The Experiment Info ‘Threshold’ parameter determines spot detection sensitivity and is inherited from TrackMate (see the TrackMate manual for a detailed description). A low threshold value detects even poorly defined spots, while a high threshold value ignores poorly defined spots. However, processing takes a long time with more than 1,000,000 spots and doesn’t yield usable results for our data and hardware. Therefore, we typically use a value of 5, 10, or 15. For new applications, it’s recommended to test spot detection quality using the original TrackMate plugin. To start the ‘Run TrackMate’ procedure, select the Glyco-PAINT section from the top-level menu and click ‘Run TrackMate’.

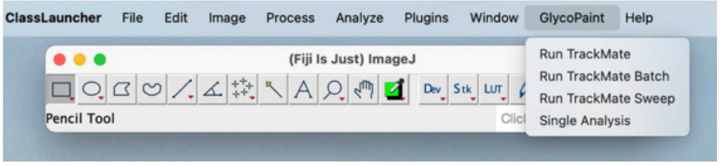

A dialogue box to select the Recordings Directory and Experiment Directory (previously created) is displayed.

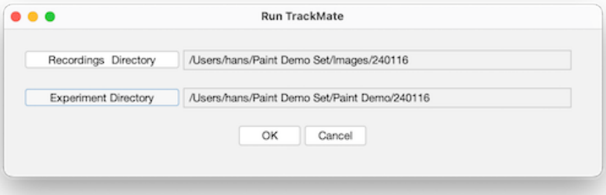

Upon successful processing (for the demo case, expect roughly 15 minutes runtime), the system has overlaid each recording with tracks as shown below.

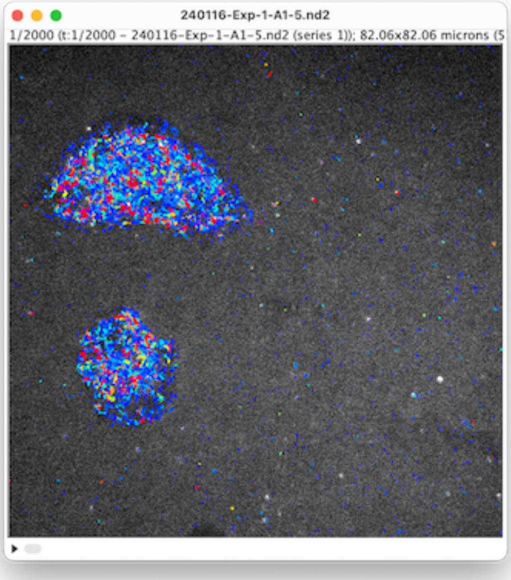

For the set of recordings in the experiment, the system generates one 'All Tracks' and one 'All Recordings' file with attributes such as Nr Spots, Nr Tracks, Run Time and a Time Stamp. The 'All Tracks' file contains, for every track, the average x and y position, the number of spots and track duration. In addition, the diffusion coefficient for a track is calculated (refer to Algorithms). An example of the first few columns of an All Tracks file is shown below.

Unique Key	Ext Recording Name	Track Label	Nr Spots	Track Duration	Track X Location	Track Y Location	Diffusion Coefficien	Square Nr	Label Nr
240116-Exp-1-A1-2-threshold-5 - 0	240116-Exp-1-A1-2-threshold-5	Track_0	233	11.75	31.36	25.48	909	127	9
240116-Exp-1-A1-2-threshold-5 - 1	240116-Exp-1-A1-2-threshold-5	Track_1	816	41.11	29.21	25.76	508	127	9
240116-Exp-1-A1-2-threshold-5 - 2	240116-Exp-1-A1-2-threshold-5	Track_2	7	0.3	78.7	25	0	139	
240116-Exp-1-A1-2-threshold-5 - 3	240116-Exp-1-A1-2-threshold-5	Track_3	3	0.1	58.18	25.11	64	134	
240116-Exp-1-A1-2-threshold-5 - 4	240116-Exp-1-A1-2-threshold-5	Track_4	2000	99.96	26.72	26.54	849	126	
240116-Exp-1-A1-2-threshold-5 - 5	240116-Exp-1-A1-2-threshold-5	Track_5	5	0.2	72.16	25.52	353	137	
240116-Exp-1-A1-2-threshold-5 - 6	240116-Exp-1-A1-2-threshold-5	Track_6	86	4.25	26.4	23.95	541	106	
240116-Exp-1-A1-2-threshold-5 - 7	240116-Exp-1-A1-2-threshold-5	Track_7	950	47.61	24.67	23.55	35	106	
240116-Exp-1-A1-2-threshold-5 - 8	240116-Exp-1-A1-2-threshold-5	Track_8	13	0.65	32.06	24.89	64	127	9
240116-Exp-1-A1-2-threshold-5 - 9	240116-Exp-1-A1-2-threshold-5	Track_9	11	0.5	55.97	24.55	77	113	
240116-Exp-1-A1-2-threshold-5 - 10	240116-Exp-1-A1-2-threshold-5	Track_10	120	6.15	24.06	25.01	2338	125	
240116-Exp-1-A1-2-threshold-5 - 11	240116-Exp-1-A1-2-threshold-5	Track_11	24	1.15	22.63	24.24	50	105	
240116-Exp-1-A1-2-threshold-5 - 12	240116-Exp-1-A1-2-threshold-5	Track_12	29	1.5	22.12	22.56	1564	105	
240116-Exp-1-A1-2-threshold-5 - 13	240116-Exp-1-A1-2-threshold-5	Track_13	145	7.35	25.67	22.44	66	106	
240116-Exp-1-A1-2-threshold-5 - 14	240116-Exp-1-A1-2-threshold-5	Track_14	10	0.45	52.03	23.11	86	112	
240116-Exp-1-A1-2-threshold-5 - 15	240116-Exp-1-A1-2-threshold-5	Track_15	1053	52.76	27.26	22.37	264	106	
240116-Exp-1-A1-2-threshold-5 - 16	240116-Exp-1-A1-2-threshold-5	Track_16	228	11.55	29.88	22.88	593	107	14
240116-Exp-1-A1-2-threshold-5 - 17	240116-Exp-1-A1-2-threshold-5	Track_17	22	1.05	27.25	22.44	6	106	
240116-Exp-1-A1-2-threshold-5 - 18	240116-Exp-1-A1-2-threshold-5	Track_18	716	36.01	21.71	20.81	174	105	
240116-Exp-1-A1-2-threshold-5 - 19	240116-Exp-1-A1-2-threshold-5	Track_19	21	1.05	42.32	20.52	13	90	40
240116-Exp-1-A1-2-threshold-5 - 20	240116-Exp-1-A1-2-threshold-5	Track_20	85	4.55	27.81	20.49	729	86	23
240116-Exp-1-A1-2-threshold-5 - 21	240116-Exp-1-A1-2-threshold-5	Track_21	22	1.15	23.49	21.87	526	105	
240116-Exp-1-A1-2-threshold-5 - 22	240116-Exp-1-A1-2-threshold-5	Track_22	21	1.1	11.03	21.16	122	102	
240116-Exp-1-A1-2-threshold-5 - 23	240116-Exp-1-A1-2-threshold-5	Track_23	1361	68.16	21.78	19.4	2018	85	7

The Project directory contents just after TrackMate has been run for both the 240104 and 240116 experiments is shown below.

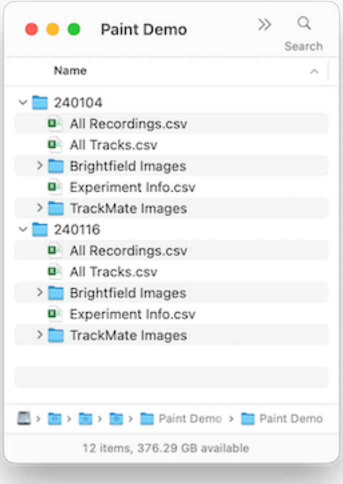

In the Glyco-PAINT menu, a second option is listed: ‘Run TrackMate Batch’. This option allows multiple projects to be processed without user intervention. For this option, a batch file needs to be provided that should contain exactly 4 columns with the following names:

1. ‘Project’, specifying the pathname where project data will be saved (analysed data)
2. ‘Image Source’, specifying the pathname where the raw recordings are saved (raw data)
3. ‘Experiment’, specifying the subfolder name of the individual experiment
4. ‘Process’, a Boolean that indicates whether the image is processed or not

Project	Image Source	Experiment	Process
/Users/hans/Paint Demo Set/Paint Demo	/Users/hans/Paint Demo Set/Images	240104	Yes
/Users/hans/Paint Demo Set/Paint Demo	/Users/hans/Paint Demo Set/Images	240116	Yes

Generate Squares

The tracks for the experiment are available in the ‘All Tracks’ file. The recordings in the Experiment folder (or for all experiments in a Project folder) are analysed by superimposing a grid over the image and determining the characteristics of each square. The user interface for this function is shown below.

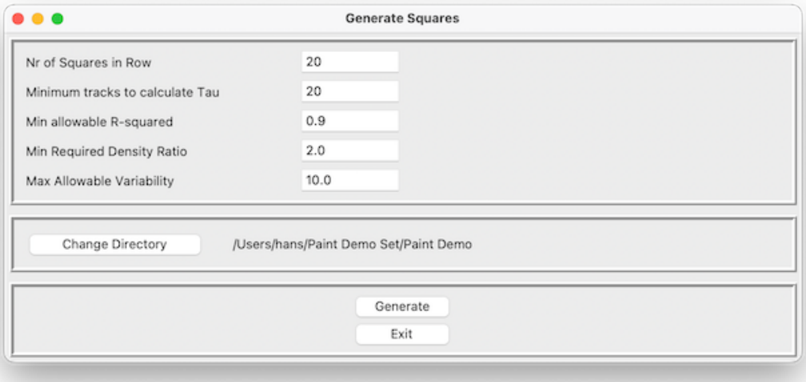

The number of squares in a row and column determines the size of the squares in the grid (typical values are 20 or 30). The number of tracks for each square is then determined. The 10% least dense squares form a 'background' density, and all square densities are compared to this. If the density ratio exceeds the 'Minimum Required Density Ratio', the square is considered sufficiently distinct from the background to be of interest, otherwise it is ignored. The homogeneity of the squares is also determined, and if requested, the lack of homogeneity (variability) can be limited, excluding squares with too much variability.

For each square that meets the specified density and variability criteria, a histogram of track durations is generated. Tau is then calculated through curve fitting. To ensure accurate curve fitting, a minimum number of data points (tracks) is required. The user can define this threshold with the 'Minimum Tracks to Calculate Tau' parameter.

The quality of curve fitting is expressed in an R2 parameter. An R^2 value of 1 indicates a perfect fit, while values lower than 0.5 indicate a low-quality fit. The user-specified 'Min allowable R-squared' parameter sets a limit to the acceptable quality of fit.

For all squares in each recording, several attributes are calculated:

- Tau and R^2 (provided that the squares meet the selection criteria)
- Density
- Diffusion Coefficient
- Number of tracks
- Duration of the tracks (median, max and total of all, median of 10% longest, median of 10% shortest)
- Speed (Median and Max of max track speed, Median and Max of mean track speed,

With the 'Generate Squares' function run, the directory structure is shown below (with now additional the All Squares files).

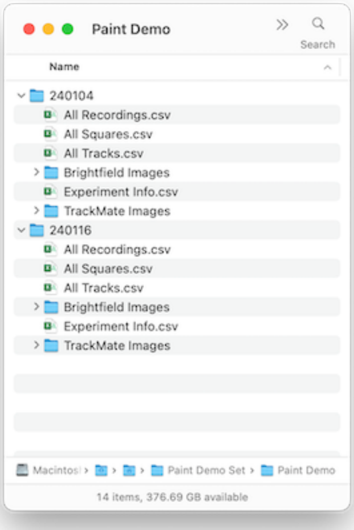

Compile Project

Typically, data to be analysed comes from more than one experiment. With the Compile project option, the data from the experiments in the project are compiled and an All Recordings, All Squares and All Tracks file, which then comprises all data of the project. Any experiment that starts with a '-' is not included, e.g. '-240116'.

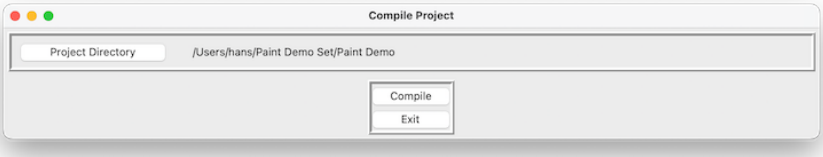

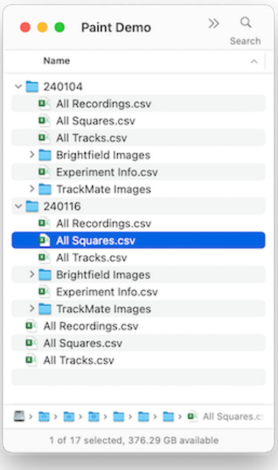

Visual Inspection

Once the squares are generated, the results can be reviewed by running the Recording Viewer utility. A straightforward dialogue enables the selection of the Project or Experiment directory.

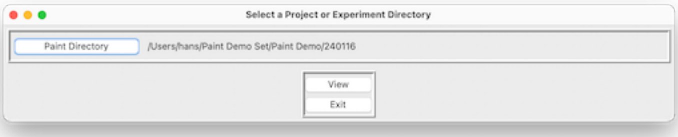

The Viewer dialogue appears, displaying the track reconstruction image on the left with overlaid squares and the corresponding brightfield image on the right. Scroll buttons at the bottom (or the combo box immediately under the 'Squares' window) allow you to select different recordings.

Squares are only shown when they meet specific criteria: density ratio exceeding a minimum, variability less than a maximum, track duration between a minimum and maximum, and the presence of a valid Tau for the square. Pressing the 'v' key also displays squares that meet these criteria but lack a valid Tau.

Below the Squares image, the metadata of the recording in view is displayed. To better view the underlying cells and tracks, keyboard options allow you to toggle between showing squares (key 's') or numbers (key 'n').

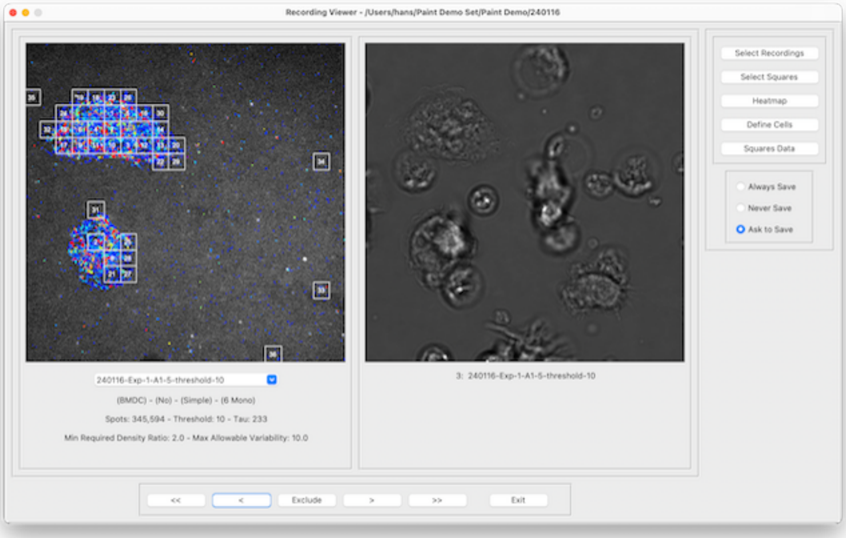

Select Recordings

The 'Select Recordings' dialogue enables users to narrow down the recordings they're viewing. For instance, in the provided example, recordings are available for two Cell Types and three Probes. If a user is only interested in BMDC recordings, they can select 'BMDC', click 'Filter', and then 'Apply All Filters' to display only the relevant recordings. The 'Reset'

buttons undo a previously made selection, while ‘Reset All’ undoes all selections previously made.

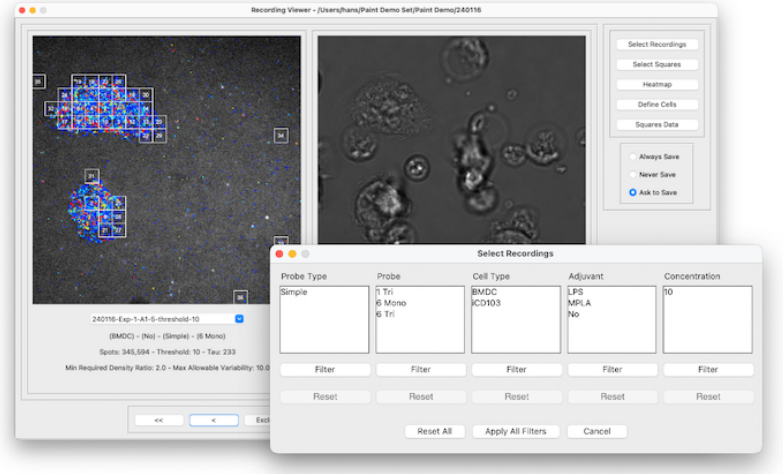

Select Squares

The Select Squares dialogue allows you to adjust the squares of interest for each recording. Sliders enable you to set the values for Max Allowable Variability, Min Required Density, and Min and Max Longest Track Duration. Squares that no longer meet these criteria will disappear, while those that do will remain visible.

The ‘Neighbour’ mode limits the spatial distribution of squares. In ‘Free’ mode, there are no restrictions. In ‘Relaxed’ mode, squares must touch at least at the corners. In ‘Restricted’ mode, they must be adjacent along the edges. The ‘Set All’ button applies the current selection criteria to all open recordings in the project.

The Recording Viewer dialogue offers three options: ‘Always Save’, ‘Never Save’, and ‘Ask to Save’. These options determine whether changes made in the viewer are saved or not when the user closes the viewer.

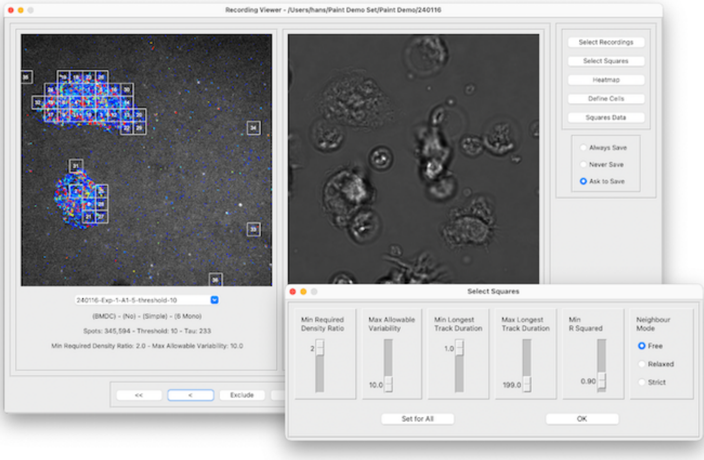

Define Cells

In the Define Cells dialogue, you can assign squares to cells. To do this, select a square by drawing a rectangle with the mouse, choose a cell (colour), and press ‘Assign’. Any changes you make will be saved when you exit the Recording Viewer, depending on your chosen save options.

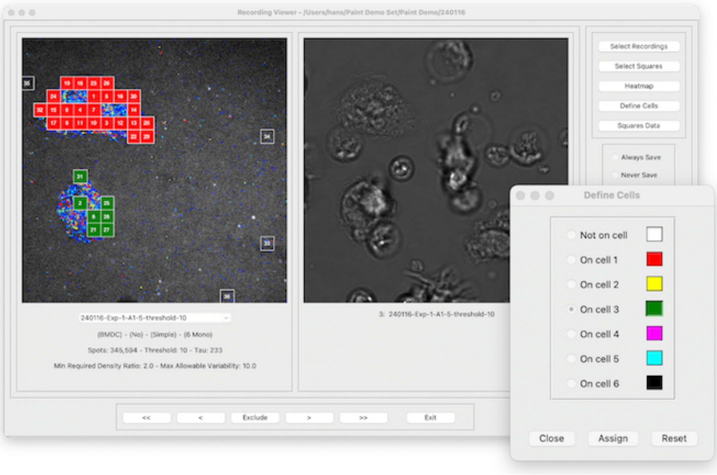

Show Heatmap

The Heatmap dialogue allows you to view the kinetic parameters of squares relative to each other. Five properties are available: Tau, Density, Average Diffusion Coefficient, Longest Track Duration, and Cumulative Track Duration. A colour range of 20 steps is used to display these values. The minimum and maximum values are limited to the current recording unless the ‘global’ option is checked, in which case they are determined for the current set of recordings. The ‘Toggle’ function, which can also be invoked by pressing ’t’, switches between the heatmap and regular display.

Note that for the heatmap values are shown for all squares, i.e., without regard to the selection status.

Whilst the Heatmap dialogue is displayed, the user can scroll through recordings.

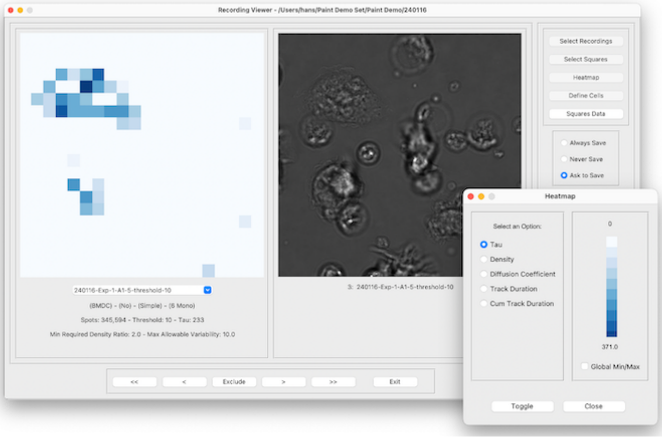

Special key bindings

In addition to the interactions that are presented through user interface elements, there are several interactions that are bound to keys.

- Key 's' toggles the display of squares.
- Key 'n' toggles the display of label numbers.
- Key 'v' toggles the display of squares that meet the filtering criteria, but for which no Tau was calculated because the number of tracks is insufficient.
- Key 'o' writes the currently displayed images to disc (with and without squares). Images are placed in the Output/Squares directory under the Experiment.
- Key 'p' saves the recordings of the current experiment to disc, with and without squares, in 'png', 'ps' and 'tiff' format. Images are placed in the Output/Squares directory under the Project or Experiment directory.

Data formats

All Recordings Format

Holding information on an experiment, two versions of All Recordings are shown. Version 1 displays the contents of All Recordings directly after TrackMate was run, including additional fields beyond the original Experiment Info file. Version 2 shows the contents after Generate Squares has been run. The Tau, Density, and R Squared values relate to those calculated for the entire field of view of the Recording. The kinetic and statistical parameters for individual squares are found in the All Squares file.

	Experiment Info	All Recordings-1	All Recordings-2	Comment
1	Recording Seq. Nr	Recording Seq. Nr	Recording Sequence Nr	Factor for selection
2	Recording Name	Recording Name	Recording Name	Factor for selection
3	Experiment Date	Experiment Date	Experiment Date	Factor for selection
4	Experiment Name	Experiment Name	Experiment Name	Factor for selection
5	Condition Nr	Condition Nr	Condition Nr	Factor for selection
6	Replicate Nr	Replicate Nr	Replicate Nr	Factor for selection
7	Probe	Probe	Probe	Meta Data
8	Probe Type	Probe Type	Probe Type	Meta Data
9	Cell Type	Cell Type	Cell Type	Meta Data
10	Adjuvant	Adjuvant	Adjuvant	Meta Data
11	Concentration	Concentration	Concentration	Meta Data
12	Threshold	Threshold	Threshold	TrackMate
13	Process	Process	Process	TrackMate
14		Nr Spots	Nr Spots	TrackMate
15		Nr Tracks	Nr Tracks	TrackMate
16		Run Time	Run Time	TrackMate
17		Ext Rec. Name	Ext Recording Name	Factor for selection
18		Recording Size	Recording Size	TrackMate
19		Time Stamp	Time Stamp	TrackMate
20			Max Frame Gap	TrackMate
21			Gap Closing Max Distance	TrackMate
22			Linking Max Distance	TrackMate
23			Median Filtering	Trackmate
24			Nr Spots in All Tracks	TrackMate

	Experiment Info	All Recordings-1	All Recordings-2	Comment
25			Min Tracks for Tau	User specified
26			Min Required R Squared	User specified
27			Nr of Squares in Row	User specified
28			Max Allowable Variability	User specified
29			Min Req. Density Ratio	User specified
30			Exclude	User specified
31			Neighbour Mode	User specified
32			Tau	Calculated
33			Density	Calculated
34			R Squared	Calculated

All Squares format

	All Squares	
1	Unique Key	
2	Recording Sequence Nr	
3	Ext Recording Name	
4	Experiment Name	
5	Experiment Date	
6	Condition Nr	
7	Replicate Nr	
8	Square Nr	
9	Probe	
10	Probe Type	
11	Cell Type	
12	Adjuvant	
13	Concentration	
14	Threshold	
15	Row Nr	
16	Col Nr	
17	Label Nr	
18	Cell Id	
19	Nr Spots	
20	Nr Tracks	
21	X0	
22	Y0	
23	X1	
24	Y1	
25	Selected	
26	Variability	Refer to ‘Variability’ in Algorithms
27	Density	Refer to ‘Density’ in Algorithms
28	Density Ratio	Refer to ‘Density’’ in Algorithms
29	Tau	Refer to ‘Tau’ in Algorithms
30	R Squared	Refer to ‘Tau’ in Algorithms
31	Median Diffusion Coefficient	Refer to ‘Diffusion Coefficient’ in Algorithms
32	Mean Diffusion Coefficient	Refer to ‘Diffusion Coefficient’ in Algorithms
33	Median Diffusion Coefficient Ext	Refer to ‘Diffusion Coefficient Ext’ in Algorithms
34	Mean Diffusion Coefficient Ext	Refer to ‘Diffusion Coefficient Ext’in Algorithms
35	Median Long Track Duration	Refer to ‘Track Duration’ in Algorithms
36	Median Short Track Duration	Refer to ‘Track Duration’in Algorithms

	All Squares	
37	Median Displacement	Refer to ‘Displacement’ in Algorithms
38	Max Displacement	Refer to ‘Displacement’ in Algorithms
39	Total Displacement	Refer to ‘Displacement’ in Algorithms
40	Median Max Speed	Refer to ‘Speed’ in Algorithms
41	Max Max Speed	Refer to ‘Speed’ in Algorithms
42	Median Mean Speed	Refer to ‘Speed’in Algorithms
43	Max Mean Speed	Refer to ‘Speed’ in Algorithms
44	Max Track Duration	Refer to ‘Track Duration’ in Algorithms
45	Total Track Duration	Refer to ‘Track Duration’ in Algorithms
46	Median Track Duration	Refer to ‘Track Duration’ in Algorithms
47	Square Manually Excluded	
48	Image Excluded	

All Tracks

Holding information on all tracks in a recording

	All Tracks
1	Unique Key
2	Ext Recording Name
3	Track Id
4	Track Label
5	Nr Spots
6	Nr Gaps
7	Longest Gap
8	Track Duration
9	Track X Location
10	Track Y Location
11	Track Displacement
12	Track Max Speed
13	Track Median Speed
14	Track Mean Speed
15	Track Max Speed Calc
16	Track Median Speed Calc
17	Track Mean Speed Calc
18	Diffusion Coefficient
19	Diffusion Coefficient Ext
20	Total Distance
21	Confinement Ratio
22	Square Nr
23	Label Nr

Algorithms

Tau

Tau is a measure used to characterise the distribution of track durations. To calculate Tau, a frequency distribution is created from the track durations. These durations are then ordered and fitted with a one-phase exponential decay curve to obtain the Tau value.

All tracks within a square are included in the Tau calculation, but it is only performed if a minimum number of tracks is present.

The quality of curve fitting is expressed in an R^2 parameter. An R^2 value of 1 indicates a perfect fit, while values lower than 0.5 indicate a low-quality fit. The user-specified 'Min allowable R-squared' parameter sets a limit to the acceptable quality of fit.

To calculate one Tau for the entire recording, all tracks within squares that meet the specified selection criteria are considered. These criteria include the minimum required density ratio, maximum allowable variability, minimum and maximum track durations, and neighbour state. These squares are identified by the 'Selected' flag, which is a separate column in all squares.csv.

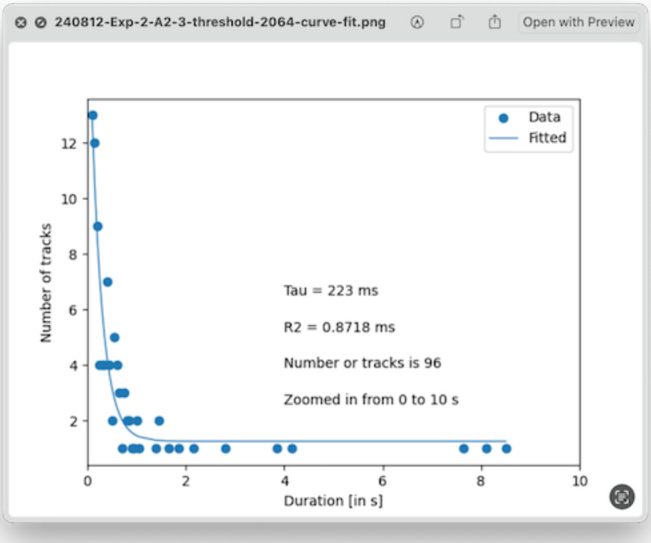

Variability

The variability of a square calculation begins with overlaying a finer grid over the existing grid and determining the number of tracks in each grid element. The variability is then calculated as the quotient of the standard deviation and the mean of the grid track numbers. The figure below illustrates the variability for four fictional squares.

1	1	1	1	1	10	10	10	10	10
1	1	1	1	1	10	10	10	10	10
1	1	1	1	1	10	10	10	10	10
1	1	1	1	1	10	10	10	10	10
1	1	1	1	1	10	10	10	10	10
1	1	1	1	1	10	10	10	10	10
1	1	1	1	1	10	10	10	10	10
1	1	1	1	1	10	10	10	10	10
1	1	1	1	1	10	10	10	10	10
1	1	1	1	1	10	10	10	10	10

Average	5.500
Standard Deviation	4.523
Variability	0.822

10	10	10	10	10	10	10	10	10	10
10	10	10	10	10	10	10	10	10	10
10	10	10	10	10	10	10	10	10	10
10	10	10	10	10	10	10	10	10	10
10	10	10	10	10	10	10	10	10	10
1	1	1	1	1	10	10	10	10	10
1	1	1	1	1	10	10	10	10	10
1	1	1	1	1	10	10	10	10	10
1	1	1	1	1	10	10	10	10	10
1	1	1	1	1	10	10	10	10	10

Average	8.000
Standard Deviation	3.763
Variability	0.470

10	10	10	10	10	10	10	10	10	10
10	10	10	10	10	10	10	10	10	10
10	10	10	10	10	10	10	10	10	10
10	10	10	10	10	10	10	10	10	10
10	10	10	10	10	10	10	10	10	10
10	10	10	10	10	10	10	10	10	10
10	10	10	10	10	10	10	10	10	10
10	10	10	10	10	10	10	10	10	10
1	1	1	1	1	10	10	10	10	10
1	1	1	1	1	10	10	10	10	10

Average	9.400
Standard Deviation	2.258
Variability	0.240

10	10	10	10	10	10	10	10	10	10
10	10	10	10	10	10	10	10	10	10
10	10	10	10	10	10	10	10	10	10
10	10	10	10	10	10	10	10	10	10
10	10	10	10	10	10	10	10	10	10
10	10	10	10	10	10	10	10	10	10
10	10	10	10	10	10	10	10	10	10
10	10	10	10	10	10	10	10	10	10
10	10	10	10	10	10	10	10	10	10
10	10	10	10	10	10	10	10	10	10

Average	10.000
Standard Deviation	0.000
Variability	0.000

Density

The Square Density is normalised to duration and concentration and is calculated with the following formula:

$$Square\ Density = \frac{Track\ Cont}{Area * Duration * Concentration}$$

The area of a square is calculated with the following data:

$$Square\ Area = \left(\frac{0.1602804 * 512}{Nr\ of\ Squares\ in\ Row}\right)^2$$

Background Density

The background 'density' represents the average track count of the lowest 10% of squares with a track count greater than zero. This measure helps distinguish squares with high track counts, likely on the cell surface, from those on the glass or background.

Density Ratio

The 'Density Ratio' is calculated by dividing the track count in a square by the background track count per square.

Squares that have a 'Density Ratio' higher than the specified 'Min Required Density Ratio' are considered of interest.

Diffusion Coefficient

The 'Diffusion Coefficient' is calculated for each track in the recording that contains three or more spots, using the following formula. Here, *n* represents the dimensionality (2), and *t* is the time interval over which displacement is measured (0.05 s).

$$MSD = \frac{1}{nr\ spots} \sum_{i=1}^{nr\ spots} (x_i - x_0)^2 + (y_i - y_0)^2$$

$$Diffusion\ Coefficient = \frac{MSD}{(2 * n * t)}$$

The 'Median Diffusion Coefficient' and 'Mean Diffusion Coefficient' values are square attributes and describe, respectively, the median and mean of the 'Diffusion Coefficient Ext' values of all tracks in the square.

Diffusion Coefficient Ext

The 'Diffusion Coefficient Ext' is a variation on the 'Diffusion Coefficient'. Here, the x and y coordinates of spot (i) are not compared to the first spot (0), but to the previous spot (i-1).

$$MSD = \frac{1}{nr\ spots} \sum_{i=1}^{nr\ spots} (x_i - x_{i-1})^2 + (y_i - y_{i-1})^2$$

$$Diffusion\ Coefficient\ Ext = \frac{MSD}{(2 * n * t)}$$

The 'Median Diffusion Coefficient Ext' and 'Mean Diffusion Coefficient Ext' values are square attributes and describe, respectively, the median and mean of the 'Diffusion Coefficient' values of all tracks in the square.

Displacement

The 'Displacement' is the distance between the start and end points of a track. The 'Median, Max and Total Displacement' are, respectively, the median, maximum and sum of the displacements of all tracks within the square.

Track Duration

The 'Track Duration' is determined by the number of spots in the track and the time between two frames (50 milliseconds in our experiments).

The median is determined of all the tracks in a square 'Median Track Duration', of the 10% longest tracks in the square 'Median Long Track Duration' and the 10% shortest tracks in the square 'Median Short Track Duration'.

The 'Max Track Duration' is the longest duration of the track durations in a square, and the 'Total Track Duration' is the sum of all track durations.

Speed

The speed of a track varies with the distance between consecutive spots, while the time between these spots remains constant. The 'max speed' of a track is the highest recorded speed, and the 'mean speed' is the average speed across all spots.

The 'Median Max Speed' and 'Max Max Speed' are square attributes that represent the median of the maximum speeds of all tracks within a square and the maximum speed of all tracks.

Similarly, the 'Median Mean Speed' and 'Max Mean Speed' are square attributes that capture the median of the maximum speeds of all tracks within a square and the maximum speed of all tracks.

Parameters

The operation of the Paint Pipeline can be tuned with parameters that are kept in 'Paint.json' file.

```
{
    "Paint": {
        "Version": "1.0",
        "Image File Extension": ".nd2",
        "Fiji Path": "/Applications/Fiji.app"
    },
    "Generate Squares": {
        "Plot to File": false,
        "Min Tracks to Calculate Tau": 20,
        "Process Recording Tau": true,
        "Min Allowable R Squared": 0.1,
        "Max Track Duration": 1000000,
        "Min Required R Squared": 0.9,
        "Min Track Duration": 0,
        "Process Square Tau": true,
        "Fraction of Squares to Determine Background": 0.1,
        "Nr of Squares in Row": 20,
        "logging": {
            "level": "INFO",
            "file": "Generate Squares.log"
        },
        "Exclude zero DC tracks from Tau Calculation": false,
        "Max Allowable Variability": 10.0,
        "Min Required Density Ratio": 2.0,
        "Plot Max": 5,
        "Neighbour Mode": "Free"
    },
     "TrackMate": {
        "MAX_FRAME_GAP": 3,
        "ALTERNATIVE_LINKING_COST_FACTOR": 1.05,
        "DO_SUBPIXEL_LOCALIZATION": false,
        "MIN_NR_SPOTS_IN_TRACK": 3,
        "LINKING_MAX_DISTANCE": 0.6,
        "MAX_NR_SPOTS_IN_IMAGE": 2000000,
        "GAP_CLOSING_MAX_DISTANCE": 1.2,
        "TARGET_CHANNEL": 1,
        "SPLITTING_MAX_DISTANCE": 15.0,
        "TRACK_COLOURING": "TRACK_DURATION",
        "RADIUS": 0.5,
        "logging": {
            "level": "INFO",
            "file": "Run Trackmate Batch.log"
        },
        "ALLOW_GAP_CLOSING": true,
        "DO_MEDIAN_FILTERING": false,
        "ALLOW_TRACK_SPLITTING": false,
        "ALLOW_TRACK_MERGING": false,
        "MERGING_MAX_DISTANCE": 15.0
    },
    "User Directories": {
        "Project Directory": "/Users/hans/Downloads/New Project",
        "Experiment Directory": "/Users/hans/Downloads/New Project",
        "Level": "Experiment",
        "Images Directory": "/Volumes/Extreme SSD/Omero/250205 BMDM FAx dish 1"
    }
}
```


The default parameters are suitable for many situations and don't require changing. However, changing parameters requires a deeper understanding of the pipeline and components like TrackMate. In our manuscript, we provide a parameter sensitivity analysis for a selection of tracking and spot detection parameters for our specific use case.

Paint

Two parameters are of interest:

- -Image File Extension: Specifies the extension of the images generated by the microscope. For example, for Nikon it is '.nb2'. Generally speaking, any tiff-compatible format is suitable.
- Fiji Path: Under normal circumstances, this does not have to be specified, as the software will detect the location of Fiji itself.

User Directories

In this section, directories previously specified by the user are stored. so they can be offered as defaults in the next component of the pipeline.

Generate Squares

In this section, parameters are stored that are used by the Generate Squares utility.

- Five parameters, Nr of Squares in Row, Min Tracks to Calculate Tau, Min Required R Squared, Min Required Density Ratio and Max Allowable Variability, have been entered previously by the user in the Generate Squares dialogue and stored so they can be presented in the next invocation of Generate Squares.

- Three other parameters, Neighbour Mode, Min Track Duration and Max Track Duration, provide values that cannot be specified in the user interfaces. The defaults are chosen so that no squares are eliminated.

- Fraction of Squares to Determine Background: Specifies the fraction of squares that are used to average the background.

TrackMate

In this section, parameters are stored that are used by TrackMate. The TrackMate documentation pages contain much useful information on TrackMate. Not parameterised, but important choices for TrackMate are the 'LoG detector' model for spot detection and the 'Simple LAP tracker' model for tracking.

Spot Detection

The **RADIUS** parameter, used during spot detection, specifies the size of tracked spots. It determines the radius of the detection filter applied to identify objects in the dataset. The value depends on factors like the microscope, recording parameters, and fluorophore used. For Glyco-PAINT experiments, a **RADIUS** of 0.5 micrometres yielded good results.

If positional accuracy is crucial, the **DO_SUBPIXEL_LOCALISATION** parameter enables or disables subpixel spot position refinement. This feature improves object tracking precision, especially with high-resolution data or small objects that don't align perfectly with pixel boundaries. However, Glyco-PAINT doesn't require high accuracy, so the parameter is set to False to avoid unnecessary processing time.

The **DO_MEDIAN_FILTERING** parameter determines whether a median filter should be applied during spot detection. This preprocessing step reduces noise and enhances object detection, especially in noisy datasets. The proposed value is True.

The **TARGET_CHANNEL** parameter specifies the image channel for spot detection in multichannel datasets. This is vital when tracking in only one channel of a dataset (e.g., fluorescence microscopy). For Glyco-PAINT, the parameter defaults to channel 1.

Building Tracks

TrackMate constructs tracks from spots in subsequent frames. The **LINKING_MAX_DISTANCE** parameter influences the linking phase of tracking. It sets the maximum spatial distance within which two spots in consecutive frames can be linked as part of the same trajectory. If the distance exceeds this limit, TrackMate assumes two separate tracks.

For example, if we expect a lectin to move a limited distance within the cell membrane in subsequent frames (50 milliseconds), a high value for **LINKING_MAX_DISTANCE** is necessary. A suitable value is 0.5 micrometre, which is equal to the expected radius of the spots.

In cases where there are multiple spot candidates for linking to a spot, the **ALTERNATIVE_LINKING_COST_FACTOR** parameter modifies TrackMate's penalty for alternative connections that are less ideal, such as those that are farther away. The recommended value for this parameter is 1.05.

Gap Closing

The **MAX_FRAME_GAP** parameter is part of the gap-closing step in the tracking process. It determines the maximum number of consecutive frames an object can disappear for and still be considered part of the same trajectory if it reappears.

Imagine a clearly defined track with spots on proximate positions defined in subsequent frames. However, in some frames, a spot is missing. Do we interpret these as distinct tracks (binding events), or do we assume that the camera simply missed a spot, and this is one long track?

If **ALLOW_GAP_CLOSING** is set to True, TrackMate will consider whether gaps in what may be one track should be closed. If the number of consecutive frames where a spot is missing is smaller than **MAX_FRAME_GAP**, and the distance between the last known and new spot location is smaller than **GAP_CLOSING_MAX_DISTANCE**, the two tracks are interpreted as one track with some spots missing. Otherwise, they are considered separate tracks.

For example, if we expect that a lectin can release a glycan and immediately bind another, a low value of **MAX_FRAME_GAP** is required.

The suggested values for **MAX_FRAME_GAP** and **GAP_CLOSING_MAX_DISTANCE** are 3 frame and 0.5 micrometre, respectively. This is because the expected radius of the spots is 0.5 micrometre.

Track Splitting

Gap splitting concerns the ability of a track to split into two or more tracks during tracking. This is commonly used in scenarios such as cell division, where a single cell divides into two daughter cells, and branching motion, where a particle splits into multiple trajectories. This is not relevant to the Glyco-PAINT application, and **ALLOW_TRACK_SPLITTING** is set to False.

Track Merging

Track merging allows two or more tracks to merge into one during tracking. This happens in scenarios like cell aggregation (two cells move together and become indistinguishable) or particle collision (two particles collide and continue as one). However, this is not relevant to the Glyco-PAINT application, so **ALLOW_TRACK_MERGING** is set to False.

Glyco-PAINT

The **MIN_NR_SPOTS_IN_TRACK** parameter is specific to Glyco-PAINT and, with its default setting of 3, ignores the shortest possible tracks, consisting of only two spots, as they are considered imaging noise.

The **TRACK_COLOURING** parameter determines the track characteristic used for colouring. Valid choices are specified in the TrackMate documentation, and options currently supported are TRACK_ID and TRACK_DURATION.

The **MAX_NR_SPOTS_IN_IMAGE** parameter prevents TrackMate from generating tracks when there are more than the specified number of spots detected. This is because, when there are many spots, tracking becomes prohibitively long, and useful results are unlikely to be achieved. If this situation occurs, processing is aborted, the number of tracks recorded is set to -1, and that recording is excluded from further processing. However, the option remains to process the recording with a higher 'Threshold' parameter (specified in the Experiment Info.csv file).

Paint directories

The Paint pipeline creates a 'Paint' directory in the user's home directory, with two subdirectories: 'Defaults' and 'Logger'.

- In the 'Defaults' directory, a 'Paint.json' holds the parameters that are used by the various components of the Paint pipeline. For regular use, parameters do not need to be changed, but the option to change is provided (but requires detailed insight into the pipeline's operation). The parameters are explained in the next section.

- In the ‘Logger’ directory, the system writes log files that provide information about the progress of operations in the pipeline and if any difficulties are encountered. The log files can be statically viewed with a regular text editor or dynamically with the macOS Console application.

Structure of Generate Squares

The core of the data processing takes place in Generate Squares. To facilitate studying the code, some high-level pseudocode is presented here.

Generate Squares main structure

```
For all the Experiments in the Project (Process Project)
    For all the Recordings in the Experiment (Process Experiment)
        For all the Squares in the Recording (Process Recording)
            Calculate Square properties (Process Square)
```

Process Project

```
1. Start project processing and log the project path.
2. List and sort all experiment directories.
3. For each directory:
  - Skip if it is not an experiment directory (e.g. if it is an 'Output' directory).
  - Skip if already processed, unless forced by a paint_force flag.
  - Call process_experiment for each unprocessed experiment.
4. Return the number of experiments processed.
```

Process Experiment

```
1. Initialise variables for tracking and logging.
2. Load track and recording data into DataFrames.
3. Validate consistency between tracks and recording data
4. Log the number of recordings to process.
5. For each recording
   - Retrieve recording details.
   - Call process_recording to process data.
   - Update experiment-level metrics with results from recording.
6. Save updated tracks, recordings, and squares data to files.
7. Log total processing time for the experiment.
```

Process Recording

```
1. Initialise processing variables.
2. Loop through all squares in the grid:
   - Calculate square coordinates.
   - Filter tracks within square boundaries.
   - Compute metrics (Tau, Density, Variability, etc.).
   - Append square data to the main DataFrame.
3. Compute the density ratio for the squares.
4. Label selected squares and propagate labels to tracks.
5. Calculate the recording level Tau and Density
6. Return processed squares, tracks, and recording-level metrics.
```

Process Square

```
1. Calculate square boundaries based on grid coordinates.
2. Filter tracks within the square.
3. If no tracks:
   - Assign default values for metrics (e.g., Tau, Density = 0).
4. If tracks exist:
   - Compute metrics (Tau, R-squared, Density, Variability, etc.).
5. Return a dictionary containing square-level data.
```
