# Supplementary material for: Subcellular glycan-mannose receptor binding kinetics correlate with myeloid cell function
Source: Nat Commun. 2025 Dec 26;17:886. doi: 10.1038/s41467-025-67602-x (PMC12830989; doi:10.1038/s41467-025-67602-x)
Supplement: Supplementary file 2 — Description of Additional Supplementary Files [file 41467_2025_67602_MOESM2_ESM.pdf]

Description of Additional Supplementary Files

File Name: Supplementary Video 1
Description: Walkthrough video with screen recording and voice instructions for performing tracking analysis using Glyco-PAINT-APP

File Name: Supplementary Video 2
Description: Walkthrough video with screen recording and voice instructions for using the Recording Viewer utility from Glyco-PAINT-APP
