# Supplementary material for: Subcellular glycan-mannose receptor binding kinetics correlate with myeloid cell function
Source: Nat Commun. 2025 Dec 26;17:886. doi: 10.1038/s41467-025-67602-x (PMC12830989; doi:10.1038/s41467-025-67602-x)
Supplement: Supplementary file 5 — Reporting Summary [file 41467_2025_67602_MOESM5_ESM.pdf]

nature portfolio

Corresponding author(s): Sander van Kasteren
Last updated by author(s): 13/8/25

Reporting Summary

Nature Portfolio wishes to improve the reproducibility of the work that we publish. This form provides structure for consistency and transparency in reporting. For further information on Nature Portfolio policies, see our Editorial Policies and the Editorial Policy Checklist.

Statistics

For all statistical analyses, confirm that the following items are present in the figure legend, table legend, main text, or Methods section.

n/a	Confirmed	
☐	☒	The exact sample size (*n*) for each experimental group/condition, given as a discrete number and unit of measurement
☐	☒	A statement on whether measurements were taken from distinct samples or whether the same sample was measured repeatedly
☐	☒	The statistical test(s) used AND whether they are one- or two-sided<br>*Only common tests should be described solely by name; describe more complex techniques in the Methods section.*
☒	☐	A description of all covariates tested
☒	☐	A description of any assumptions or corrections, such as tests of normality and adjustment for multiple comparisons
☐	☒	A full description of the statistical parameters including central tendency (e.g. means) or other basic estimates (e.g. regression coefficient) AND variation (e.g. standard deviation) or associated estimates of uncertainty (e.g. confidence intervals)
☐	☒	For null hypothesis testing, the test statistic (e.g. *F*, *t*, *r*) with confidence intervals, effect sizes, degrees of freedom and *P* value noted<br>*Give P values as exact values whenever suitable.*
☒	☐	For Bayesian analysis, information on the choice of priors and Markov chain Monte Carlo settings
☒	☐	For hierarchical and complex designs, identification of the appropriate level for tests and full reporting of outcomes
☐	☒	Estimates of effect sizes (e.g. Cohen's *d*, Pearson's *r*), indicating how they were calculated

Our web collection on statistics for biologists contains articles on many of the points above.

Software and code

Policy information about availability of computer code

Data collection	NIS Elements is the commercial software package that is used to acquire microscope images on Nikon Ti-2 N-STORM system. BD FACS Diva and InCyte were used to acquire flow data.
Data analysis	Flow cytometry data was analysed using FlowJo v10.8. Statistical analysis was performed using R Studio.<br><br>Single Particle Tracking of microscopy videos is performed using TrackMate (TrackMate 7: integrating state-of-the-art segmentation algorithms into tracking pipelines | Nature Methods. https://www.nature.com/articles/s41592-022-01507-1)<br><br>The Glyco-PAINT-APP software package is available through GitHub (github.com/jjabakker/GlycoPaint-Pipeline) including a installation manual and software manual with accompanying demo dataset which is available via Zenodo (https://zenodo.org/records/14196381).

For manuscripts utilizing custom algorithms or software that are central to the research but not yet described in published literature, software must be made available to editors and reviewers. We strongly encourage code deposition in a community repository (e.g. GitHub). See the Nature Portfolio guidelines for submitting code & software for further information.

Data

Policy information about availability of data

All manuscripts must include a data availability statement. This statement should provide the following information, where applicable:

- Accession codes, unique identifiers, or web links for publicly available datasets
- A description of any restrictions on data availability
- For clinical datasets or third party data, please ensure that the statement adheres to our policy

Complete processed microscopy datasets generated during the current study will be deposited on Zenodo. Raw miscopy recordings are available upon request due to file size limitations. Demo datasets to evaluate the software are available via Zenodo and linked to the GitHub page (both are listed above).

Research involving human participants, their data, or biological material

Policy information about studies with human participants or human data. See also policy information about sex, gender (identity/presentation), and sexual orientation and race, ethnicity and racism.

Reporting on sex and gender	n.a.
Reporting on race, ethnicity, or other socially relevant groupings	n.a.
Population characteristics	n.a.
Recruitment	n.a.
Ethics oversight	n.a.

Note that full information on the approval of the study protocol must also be provided in the manuscript.

Field-specific reporting

Please select the one below that is the best fit for your research. If you are not sure, read the appropriate sections before making your selection.

☒ Life sciences ☐ Behavioural & social sciences ☐ Ecological, evolutionary & environmental sciences

For a reference copy of the document with all sections, see nature.com/documents/nr-reporting-summary-flat.pdf

Life sciences study design

All studies must disclose on these points even when the disclosure is negative.

Sample size	For all Glyco-PAINT experiments at least 3 biological replicates (independent experiments with fresh mouse material or new passage number for cell lines) with 3 technical replicates (fields of view per condition) were recorded. For flow cytometry assays at least 3 biological replicates with 2 technical replicates per condition were conducted.
Data exclusions	Cells that lose viability or show signs of apoptosis (blebbing) were removed from the microscopy studies.
Replication	Reproducibility was confirmed by repeat experiments. All repeated experiments yielded similar results. The number of experiments performed can be found in the figure legends.
Randomization	Samples were prepared from the same batch of cells on different wells, and we assigned each sample randomly to each well of cells. Cells for microscopy recordings were randomly selected, however, a visual viability check of the cell was preformed before and after measurement.
Blinding	No blinding in this study was needed as the conditions for image acquisition and analysis were kept constant through all samples

Reporting for specific materials, systems and methods

We require information from authors about some types of materials, experimental systems and methods used in many studies. Here, indicate whether each material, system or method listed is relevant to your study. If you are not sure if a list item applies to your research, read the appropriate section before selecting a response.

Materials & experimental systems

n/a	Involved in the study
	[x] Antibodies
	[x] Eukaryotic cell lines
[x]	Palaeontology and archaeology
	[x] Animals and other organisms
[x]	Clinical data
[x]	Dual use research of concern
[x]	Plants

Methods

n/a	Involved in the study
[x]	ChIP-seq
	[x] Flow cytometry
[x]	MRI-based neuroimaging

Antibodies

Antibodies used	TruStain FcX (Biolegend, 101319, 1:100), CD11c -eFluor450 (clone: N418, eBioscience, 48-0114-82, 1:200), CD206-AF488 (clone: MR5D3, Biorad, MCA2235A488T, 1:20), CD8a - APC (clone: 53-7.6, Biolegend, 100711, 1:200), TCR V beta 5.1/5.2 - eFluor450 (clone: MR9-4, eBioscience, 48-5796-82), CD86-PerCP (Biolegend, cat no 105025, clone GL-1, 1:200), F4/80-APC-Cy7 (Biolegend, cat no 123117, clone BM8, dilution 1:400), MHC-II-AF488 (Biolegend, cat no 107615, clone M5/114.15.2, dilution 1:1000), LAMP1-AF488 (BioLegend, cat no 121607, clone 1D4B, dilution 1:100) and EEA1-AF594 (MBL Life Sciences, cat no M176-A59, clone 3C10, dilution 1:100)
Validation	Antibody reactivity was verified by the manufacturers.

Eukaryotic cell lines

Policy information about cell lines and Sex and Gender in Research

Cell line source(s)	CHO-MR cell line was gifted by Luisa Martinez-Pomares
Authentication	Cell line was not authenticated via sequencing but expression of the MR was verified using flow cytometry and confocal microscopy
Mycoplasma contamination	Cell line was regularly tested for mycoplasma contamination and the result was negative
Commonly misidentified lines (See ICLAC register)	n.a.

Animals and other research organisms

Policy information about studies involving animals; ARRIVE guidelines recommended for reporting animal research, and Sex and Gender in Research

Laboratory animals	Organs from male C57Bl/6J and OT-I (C57BL/6-Tg(TcraTcrb)1100Mjb/J) mice were used in this study after cervical dislocation. Bone marrow from MR-/- mice on C57Bl/6 background were gifted by Christian Kurts (University of Bonn, Germany).
Wild animals	n.a.
Reporting on sex	Male mice were used for harvesting and differentiation of immune cells in this study to minimize variability which was considered of extra importance during development of a new technique. The study of sex differences is beyond the scope of the current study.
Field-collected samples	n.a.
Ethics oversight	All animal experiments received approval from the Dutch Central Authority for Scientific Procedures on Animals (CCD) on license number AVD1060020198832 and were conducted in accordance with the European Union Directive 2010/63/EU, recommendation 2007/526/EC

Note that full information on the approval of the study protocol must also be provided in the manuscript.

Plants

Seed stocks	*Report on the source of all seed stocks or other plant material used. If applicable, state the seed stock centre and catalogue number. If plant specimens were collected from the field, describe the collection location, date and sampling procedures.*
Novel plant genotypes	*Describe the methods by which all novel plant genotypes were produced. This includes those generated by transgenic approaches, gene editing, chemical/radiation-based mutagenesis and hybridization. For transgenic lines, describe the transformation method, the number of independent lines analyzed and the generation upon which experiments were performed. For gene-edited lines, describe the editor used, the endogenous sequence targeted for editing, the targeting guide RNA sequence (if applicable) and how the editor was applied.*
Authentication	*Describe any authentication procedures for each seed stock used or novel genotype generated. Describe any experiments used to assess the effect of a mutation and, where applicable, how potential secondary effects (e.g. second site T-DNA insertions, mosiacism, off-target gene editing) were examined.*

Flow Cytometry

Plots

Confirm that:

- [x] The axis labels state the marker and fluorochrome used (e.g. CD4-FITC).
- [x] The axis scales are clearly visible. Include numbers along axes only for bottom left plot of group (a 'group' is an analysis of identical markers).
- [x] All plots are contour plots with outliers or pseudocolor plots.
- [x] A numerical value for number of cells or percentage (with statistics) is provided.

Methodology

Sample preparation	CFSE dilution in OT-I T cells<br>Cells were washed with FACS buffer (PBS with 2 mM EDTA, 2% FCS and 7.4 mM NaN3) and stained with Aqua Live/DEAD (Invitrogen, L34957, 1:500), TruStain FcX (Biolegend, 101319, 1:100), CD8a - APC (clone: 53-7.6, Biolegend, 100711, 1:200), and TCR V beta 5.1/5.2 - eFluor450 (clone: MR9-4, eBioscience, 48-5796-82) for 30 min on ice, washed two times (by centrifugation for 5 min, 300 rcf at r.t.) and acquired on a BD Fortessa I<br><br>Glycan uptake<br>Zombie Yellow (Biolegend, 423103, 1:500), TruStain FcX (Biolegend, cat no 101319, 1:100), CD11c - eFluor450 (clone: N418, eBioscience, cat no 48-0114-82, dilution 1:200), CD206-AF488 or AF647 (clone: MR5D3, Biorad, MCA2235A488T, 1:20), CD86-PerCP (Biolegend, cat no 105025, clone GL-1, 1:200), F4/80-APC-Cy7 (Biolegend, cat no 123117, clone BM8, dilution 1:400), MHC-II-AF488 (Biolegend, cat no 107615, clone M5/114.15.2, dilution 1:1000) for 30 min. on ice. Then cells were washed twice with PBS + 2% FCS + 2 mM EDTA and acquired on Guava EasyCyte 12HT. For BMDM uptake and characterization, an identical procedure was followed but acquisition was performed on Sony ID7000 spectral flow cytometer
Instrument	BD Fortessa I, Guava EasyCyte 12HT and Sony ID7000
Software	FlowJo v10.8.1
Cell population abundance	Indicated in plots
Gating strategy	Cells, Singlets, Live cells, (for OT-I:CD8a+ & TCRvB5.1/5.1+, for BMDC: CD11c+, for BMDM, CD11b+F4/80+

- [x] Tick this box to confirm that a figure exemplifying the gating strategy is provided in the Supplementary Information.
